# N-Sulfenylation of β-Lactams: Radical Reaction of *N*-Bromo-azetidinones by TEMPO Catalysis

**DOI:** 10.1021/acs.joc.3c01759

**Published:** 2023-09-28

**Authors:** Valentina Giraldi, Francesco Giunchino, Maria Edith Casacchia, Andrea Cantelli, Marco Lucarini, Daria Giacomini

**Affiliations:** † Department of Chemistry a “Giacomo Ciamician”, University of Bologna, Via Piero Gobetti, 87, Bologna 40129, Italy; ‡ Department of Physical and Chemical Sciences, University of Aquila, Via Vetoio, Coppito, L’Aquila 67100, Italy

## Abstract

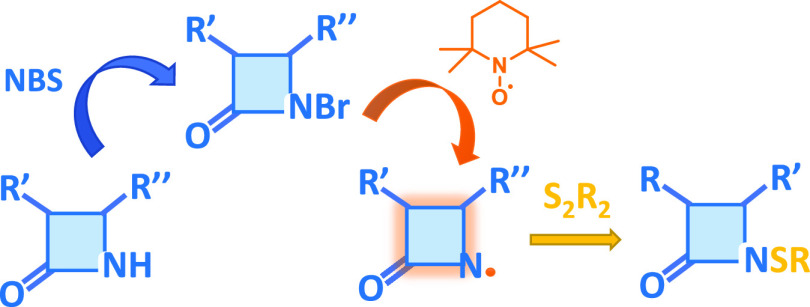

Azetidinones with a sulfenyl group on the β-lactam nitrogen atom show interesting biological activities as antimicrobial agents and enzyme inhibitors. We report in the present study a versatile synthesis of *N*-sulfenylated azetidinones starting from the corresponding *N*-bromo derivatives by means of the (2,2,6,6-tetramethylpiperidin-1-yl)oxyl (TEMPO) radical as the catalyst and disulfides. Preparation of *N*-halo-azetidinones was studied and optimized. The reactivity of *N*-bromo-azetidinone **2a** as a model compound in the presence of TEMPO radical was investigated by NMR and electron paramagnetic resonance (EPR) spectroscopy studies. Optimization of the reaction conditions allowed the access of *N*-alkylthio- or *N*-arylthio-azetidinones from 55 to 92% yields, and the method exhibited a good substrate scope.

## Introduction

**Figure 1:**
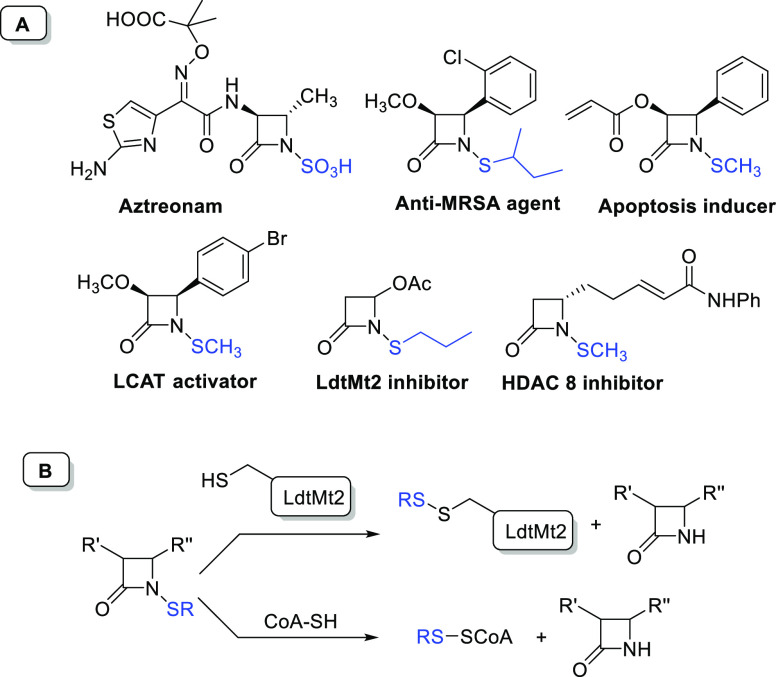
(A) Selected bioactive *N*-thiolated azetidinones. (B) Sulfenyl group transfer in LdtMt2 inhibition and in reaction with coenzyme A for antibacterial activity.

*N*-Sulfenyl-azetidinones have emerged some years ago as new members of the class of bioactive β-lactam molecules.^1^ Just after the discovery of the monocyclic β-lactam aztreonam (Figure 1) which has a *N*-sulfonic group,^2^ Miller reported a study on *N*-sulfenyl-β-lactam derivatives^3^ that were further deeply investigated by Turos and co-workers for their antimicrobial,^4^ anticancer,^5^ and antifungal activities.^1^ Later, some more bioactivities were discovered, such as the ability of *N*-sulfenyl-azetidinones to inhibit β-lactamases of resistant bacterial strains,^1^ to activate the lecithin-cholesterol acyltransferase enzyme, whose deficiency is implicated in several cholesterol-dependent diseases (Figure 1A),^6^ and to selectively inhibit the histone deacetylase protein HDAC8 significantly overexpressed in many cancer cells.^7^

When considering the mechanism of bioactivity for *N*-alkylthio-azetidinones, since the beginning it has shown a mechanism different from the ring-opening mechanism of classical β-lactam compounds,^8^ and recently it was elucidated for antitubercular activity.^9^

It was demonstrated that the inhibition of transpeptidase Ldt_Mt2_ of *Mycobacterium tuberculosis* occurs on transfer of the thio residue from the nitrogen atom of the β-lactam to the cysteine residue of the active site of the transpeptidase, thus forming a covalent disulfide adduct with the protein, as revealed by mass spectrometry (Figure 1B).^9^

The facility to transfer the *N*-thio group from *N*-sulfenylated azetidinones was also demonstrated for the antibacterial activity against *Staphylococcus aureus*. In that case, *N*-alkylthio-β-lactams transfer the thio group to coenzyme A to form mixed disulfide species (Figure 1B).^8^ The effect of different *N*-thio residues was investigated for linear and branched *N*-sulfenyl derivatives for anticancer^10^ and antibacterial activities,^1,11^ and it was ascertained that it strictly depends on the lipophilicity of residues. So, with the aim of discovering new and potent compounds, it would be meaningful to have a versatile and robust methodology to insert sulfenyl residues to get differently substituted *N*-thiolated azetidinones.

**Figure 2:**
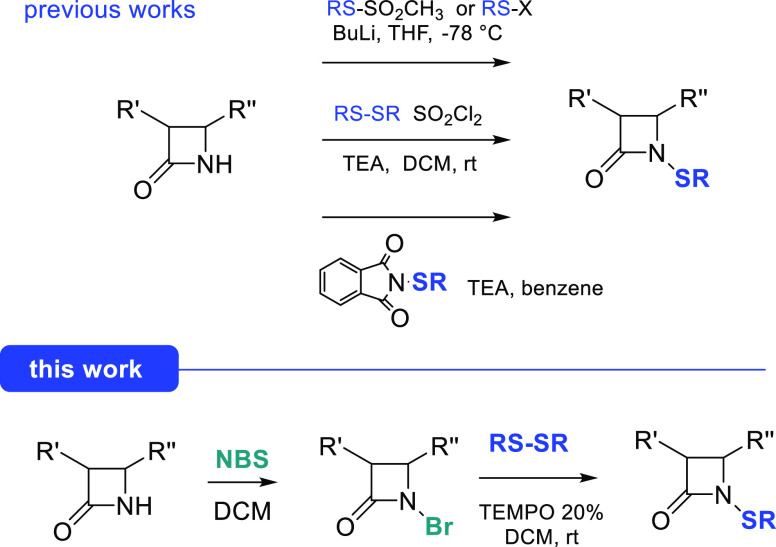
Previously developed syntheses of *N*-alkylthio-azetidinones in comparison with the present work.

Few methods are known for the insertion of an alkylthio residue on the nitrogen atom of azetidinones (Figure 2). Starting from *S*-methyl thiomethanesulfonate, the corresponding *N*-methylthio-azetidinones can be obtained but with the use of *n*BuLi at low temperature under an inert atmosphere.^12^ The harsh reaction conditions of this procedure could, however, limit its application. Our group developed a procedure with dialkyl- or diaryl-disulfides and sulfuryl chloride which, however, has severe hazards for acute toxicity.^13^ N-Sulfenylation could finally be obtained with alkyl- or arylthio-phthalimides which, in turn, are prepared from a disulfide and sulfuryl chloride, but with the same concerns described above.^8^^a,^^14^

The aim of the present work is to establish a new route to obtain *N*-sulfenyl-β-lactam derivatives. We envisaged the possibility to get N-sulfenylation by means of a radical-based strategy to transfer a sulfenyl group starting from *N*-halo-azetidinones and disulfides in the presence of (2,2,6,6-tetramethylpiperidin-1-yl)oxyl (TEMPO) as a promoter (Figure 2). At first, we investigated the synthesis of *N*-halo-azetidinones and their characterization and finally their application in the synthesis of *N*-alkyl- or *N*-arylthio-β-lactam derivatives.

## Results and Discussion

Among methods already reported in the literature, N-sulfenylation of amides could be achieved starting from disulfides under oxidative conditions with *N*-halo-succinimide and TEMPO as a promoter.^15^ Then, we postulated to apply the same strategy via the corresponding *N*-halo-azetidinones, disulfides, and TEMPO. *N*-Halo-β-lactam compounds have already been reported in the literature but poorly investigated.^12,16^ We then began exploring the synthesis of *N*-bromo-azetidinones by means of *N*-bromo-succinimide (NBS) in dichloromethane (DCM) with two commercially available 4-acetoxy-azetidinones **1a** and **1b** and azetidinones **1c–h** obtained with known procedures (see Supporting Information) (Table 1).

**Table 1:**
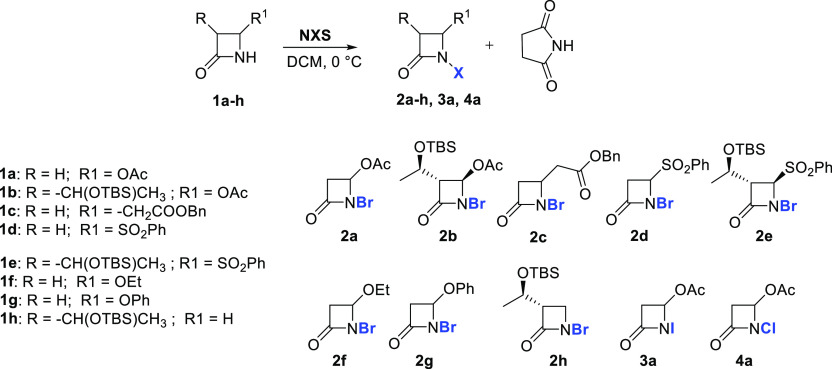
Synthesis of *N*-Halo-azetidinones **2a–h**, **3a**, and **4a** and Optimization of Reaction Conditions^a^

entry	**1a–h** (mmol)	[**1a–h**] (M)	NXS (equiv)	time (h)	yield %^b^ (product)
**1**	**1a** (0.4)	0.1	NBS (3)	3	67 (**2a**)
**2**	**1a** (0.4)	0.1	NBS (2)	1.5	69 (**2a**)
**3**	**1a** (0.4)	0.1	NBS (1)	3	74 (**2a**)
**4**	**1a** (2.4)	0.2	NBS (1)	3	81 (**2a**)
**5**	**1a** (2.4)	0.4	NBS (1 + 0.25)	5	96 (**2a**)
**6**	**1b** (0.6)	0.4	NBS (1 + 0.25)	5	28 (**2b**)
**7**	**1b** (0.6)	0.1	NBS (2)	3	96 (**2b**)
**8**	**1c** (0.5)	0.4	NBS (1 + 0.25)	5	74 (**2c**)
**9**	**1c** (0.5)	0.1	NBS (2)	5	97 (**2c**)
**10**	**1d** (0.5)	0.1	NBS (2)	4	58 (**2d**)
**11**	**1e** (0.1)	0.1	NBS (2)	3	86 (**2e**)
**12**	**1f** (0.9)	0.4	NBS (2)	1	15 (**2f**)
**13**	**1g** (0.3)	0.4	NBS (1)	1	81 (**2g**)
**14**	**1h** (0.44)	0.4	NBS (1)	2	89 (**2h**)
**15**	**1a** (0.9)	0.3	NIS (2)	4	71 (**3a**)
**16**	**1a** (1.2)	0.3	NCS (4)	7	42 (**4a**)

a Reaction conditions: Experimental Section general procedure GP1, inert atmosphere (N_2_), 0 °C, TLC monitoring.

b Isolated yields after flash chromatography.

The reaction conditions were preliminarily evaluated on azetidinone **1a** as a model compound. The reaction of **1a** was conducted with 1 equiv of NBS in anhydrous DCM at 0 °C and in an inert atmosphere. After consumption of the starting azetidinone (thin-layer chromatography (TLC) monitoring), the expected *N*-bromo-azetidinone **2a** was isolated by flash chromatography in 74% yields (Table 1, entry 3).

It was observed that the amount of NBS did not affect the yields, which instead depended on the concentration (Table 1, entries 1–5), and the best conditions obtained with a 0.4 M solution gave excellent isolated yields, 96%, of **2a** after flash chromatography (Table 1, entry 5). However, the same reaction conditions on azetidinones **1b** and **1c**, gave lower yields, 28 and 74%, respectively (Table 1, entries 6 and 8). Instead, 0.1 M concentration and 2 equiv of NBS gave excellent yields of **2b** and **2c** (Table 1, entries 7 and 9). Compounds **2e**, **2g**, and **2h** were obtained with the optimized conditions in good yields (Table 1, entries 11, 13, and 14), whereas azetidinone **2f** was obtained in very poor yields (Table 1, entry 12) probably because of its poor stability to flash chromatography, and, moreover, it was observed that the pure compound **2f** fully decomposed in 72 h on storage at 4 °C. The synthesis of *N*-chloro and *N*-iodo analogues were tentatively investigated over azetidinone **1a**, with *N*-chloro- and *N*-iodo-succinimide (NCS and NIS), respectively. Under the optimized conditions obtained for *N*-bromination, with NIS, the conversion was still incomplete after 4 h, and the *N*-iodo-β-lactam **3a** was obtained in poor yields (28%). On increasing the molar concentration to 0.4 M, the conversion was complete, and **3a** was obtained with 71% isolated yields (Table 1, entry 15). However, it should be noted that the isolated product was quite unstable, and it released I_2_ and azetidinone **1a**.^16b^ Regarding *N*-chloro-azetidinone **4a**, the conversion was not complete after 7 h even with 4 equiv of NCS, obtaining only 42% yield after flash chromatography (Table 1, entry 16). The sulfenylation reaction of *N*-bromo-azetidinones has been previously reported by an electrooxidation reaction but scantly investigated.^17^ We decided to try the procedure reported by Sun et al., who treated NCS with disulfides and TEMPO to obtain *N*-thio-substituted succinimides.^15^ The reaction between *N*-bromo-azetidinone **2a** and diphenyl disulfide was thus investigated as a model reaction to optimize the reaction conditions. Different parameters were considered: solvents, radical initiators, concentration of **2a**, ratio between the reagents, and temperature (Table 2). Reactions were performed under a nitrogen atmosphere and anhydrous conditions. After the work up, a simple solvent evaporation, crude reaction mixtures were analyzed by ^1^H NMR in order to establish the ratio between *N*-phenylthio-azetidinone **5**, the unreacted starting material **2a**, and the byproduct 4-acetoxy-azetidinone **1a**; the isolated yields of **5** were determined after flash chromatography (Table 2, general procedure GP2). In the absence of a radical initiator, the reaction did not proceed (Table 2, entry 1), and the crude reaction mixture showed the starting *N*-bromo-azetidinone **2a** and traces of the corresponding NH-derivative **1a**. A preliminary solvent screening confirmed DCM as the best solvent among acetonitrile (ACN), tetrahydrofuran (THF), and *N*,*N*-dimethylformamide (DMF) (Table 2, entries 2–5), and hydrocarbons such as hexane or cyclohexane were not suitable because of the insolubility of the starting **2a**. In THF or ACN, despite the complete conversion of **2a**, an insoluble mixture of byproducts was obtained (Table 2, entries 2, and 3); in particular, the starting compound **2a** was unstable and completely decomposed into a complex mixture of byproducts, and neither the desired product **5** nor **1a** was observed in the crude mixture. In DMF (Table 2, entry 4), only traces of product **5** were obtained, whereas in DCM at room temperature the product **5** was isolated in 44% yield (Table 2, entry 5). Reactions at 0 °C or reflux did not show any improvement of the yields (Table 2, entries 5, 7, and 8); we observed a positive effect with 0.4 M concentration of **2a** , with a 49% isolated yield of **5** (Table 2, entry 9). On increasing the amount of TEMPO to 20 mol %, 82% yield of **5** was successfully obtained; however, higher amounts or stepwise additions of 30 mol % were detrimental (Table 2, entries 10, 11 and 12). Other radical initiators such as benzoyl peroxide and benzophenone, which need UV activation at 254 nm, or AIBN (Table 2, entries 13–15), were tried, but only traces of **5** were detected in the crude mixtures. Only 4-OH TEMPO and ABTS gave the expected product in 23 and 32% yields, respectively, but with a great amount of byproduct **1a** (Table 2, entries 16 and 17). Finally, on testing how the equivalents of diphenyl disulfide could influence the yields, on doubling the amount, good yields of **5** (84%) were obtained but without a significant improvement with respect to the use of 1 equiv (82%) (Table 2, entries 10 and 19). Moreover, with 0.5 equiv, we obtained **5** in only 74% yields (Table 2, entry 18). The final reaction mixtures were deep brown solutions that showed positive results with cyclohexene in a control test for the presence of molecular bromine.

**Table 2:**
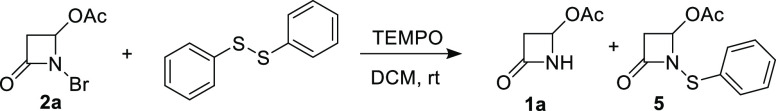
*N*-Phenyl Sulfenylation of Azetidinone **2a** and Optimization of Reaction Conditions^a^

entry	radical promoter (equiv)	Ph_2_S_2_ (equiv)	solvent	[**2a**] (M)	time	temperature	**2a**:**1a**:**5**^b^	**5** (yield %)^c^
1		1	DCM	0.4	2 h	Rt	1/0.15/0	
2	TEMPO (0.1)	1	ACN	0.15	overnight	Rt	0/0/0^d^	
3	TEMPO (0.1)	1	THF	0.15	overnight	Rt	0/0/0^d^	
4	TEMPO (0.1)	1	DMF	0.15	overnight	Rt	0/0/tr^d^	
5	TEMPO (0.1)	1	DCM	0.15	overnight	Rt	0/0.34/1	44
6	TEMPO (0.1)	1	DCM	0.08	overnight	Rt	0/0.27/1	24
7	TEMPO (0.1)	1	DCM	0.08	overnight	0 °C	0/0.31/1	19
8	TEMPO (0.1)	1	DCM	0.15	5 h	reflux	0/tr/tr^d^	
9	TEMPO (0.1)	1	DCM	0.4	overnight	Rt	0/0.09/1^d^	49
10	TEMPO (0.2)	1	DCM	0.4	5 h	Rt	0/0.10/1	82
11	TEMPO (0.3)	1	DCM	0.4	5 h	Rt	0/0.33/1	65
12^e^	TEMPO (0.1 × 3)	1	DCM	0.4	5 h	Rt	0/0.18/1	78
13^f^	benzophenone (0.2)	1	DCM	0.4	5 h	Rt	0/0/0^d^	
14^f^	benzoyl peroxide (0.2)	1	DCM	0.4	5 h	Rt	0/tr/0^d^	
15	AIBN (0.2)	1	DCM	0.4	5 h	reflux	0/tr/0^d^	
16	4-OH TEMPO (0.2)	1	DCM	0.4	5 h	Rt	0/0.9/1	23
17	ABTS (0.2)	1	DCM	0.4	5 h	Rt	0/0.6/1	32
18	TEMPO (0.2)	0.5	DCM	0.4	overnight	Rt	0/0.15/1	74
19	TEMPO (0.2)	2	DCM	0.4	5 h	Rt	0/0.07/1	84
20	TEMPO (0.2)	2	DCM	0.15	overnight	Rt	0/0.03/1	63

a Reaction conditions: **2a** (1 equiv, 0.2 mmol, 42 mg), anhydrous DCM, diphenyl disulfide, TEMPO, nitrogen atmosphere in Schlenk tube, rt; work up by solvent evaporation.

b Ratio determined by ^1^H NMR analysis on the reaction crude.

c Isolated yields after purification by flash chromatography.

d Presence of byproducts.

e TEMPO portions every 1h 40 min.

f Activation of the radical promoter by irradiation at 254 nm.

Then, the reaction scope was explored. First, various disulfides were tested with *N*-bromo-4-acetoxy-azetidinone **2a** under optimized conditions (Table 3). Only for obtaining compounds **10** and **12** (Table 3, entries 5 and 7), the concentration was reduced due to the poor solubility of the starting disulfides. The conversion was always complete, and moderate to good isolated yields were obtained for all products **6–13**, showing great tolerance to the methodology for disulfides.

**Table 3:**
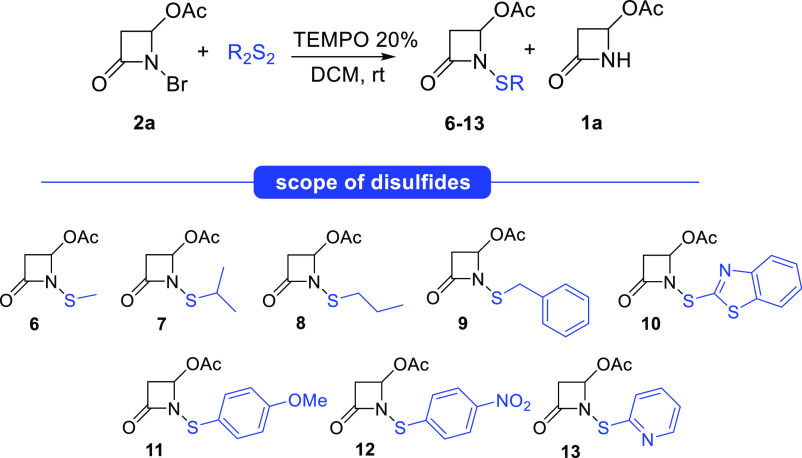
Substrate Scope for Disulfides^a^

entry	time (h)	[**2a**] (M)	**2a**/**1a**/**6–13**^b^	product (yield %)^c^
1	5	0.4	0/0.15/1	**6** (75)
2	16	0.4	0/0.50/1	**7** (64)
3	16	0.4	0/0.27/1	**8** (74)
4	5	0.4	0/0.17/1	**9** (72)
5^d^	16	0.2	0/0.20/1	**10** (70)
6	5	0.4	0/0.03/1	**11** (82)
7^d^	16	0.2	0/0.15/1	**12** (55)
8	5	0.4	0/0.05/1	**13** (78)

a Reaction conditions: **2a** (1 equiv, 0.2 mmol), anhydrous DCM (0.5 mL), disulfide (1 equiv, 0.2 mmol), TEMPO (0.2 equiv, 0.04 mmol), N_2_ atmosphere, rt; work up by solvent evaporation.

b Ratio determined by ^1^H NMR analysis on the crude reaction mixture.

c Isolated yields after flash chromatography.

d Reaction conditions: **2a** (1 equiv, 0.2 mmol) and anhydrous DCM (1 mL).

The byproduct **1a** was obtained in large amounts with *i*Pr_2_S_2_ (Table 3, entry 2), thus raising a likely issue of steric hindrance. In the case of compound **12**, the lower yield (55%) was due to difficult purification by flash chromatography. Next, with the optimized conditions, diphenyl-, diisopropyl- and dibenzyl-disulfides were selected to react with *N*-bromo-β-lactams **2b**, **2c**, **2g**, and **2h** (Table 4).

**Table 4:**
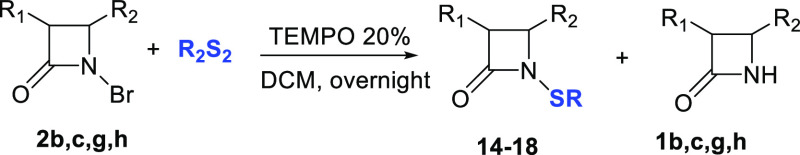

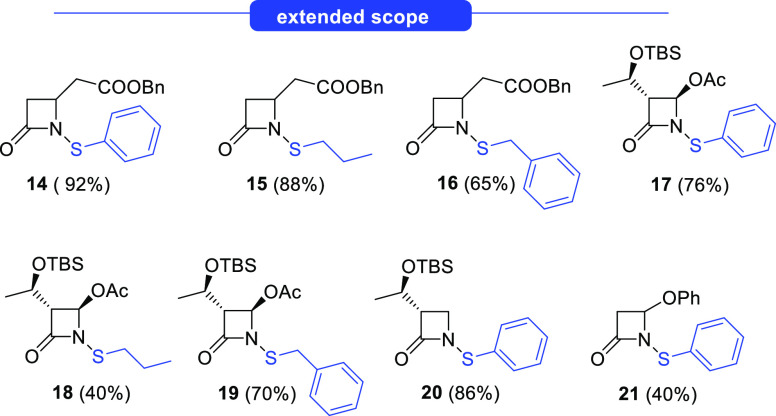
Extension of the Substrate Scope^a^

a Reaction conditions: **2b**, **2c**, **2g**, and **2h** (1 equiv, 0.2 mmol), anhydrous DCM (0.5 mL), disulfide (1 equiv, 0.2 mmol), and TEMPO (0.2 equiv, 0.04 mmol).

Excellent yields were obtained in the case of **14** and **15**, with no formation of the corresponding NH byproduct **1c**. With azetidinone **2b**, the results were comparable to those obtained with **2a**, with a lower yield in the case of the *S*-propyl derivative **18.**

To investigate the reaction mechanism of the sulfenylation reaction and the formation of NH-azetidinone, we conducted extended experiments of ^1^H NMR monitoring and electron-paramagnetic resonance spectroscopy (EPR).

**Figure 3:**
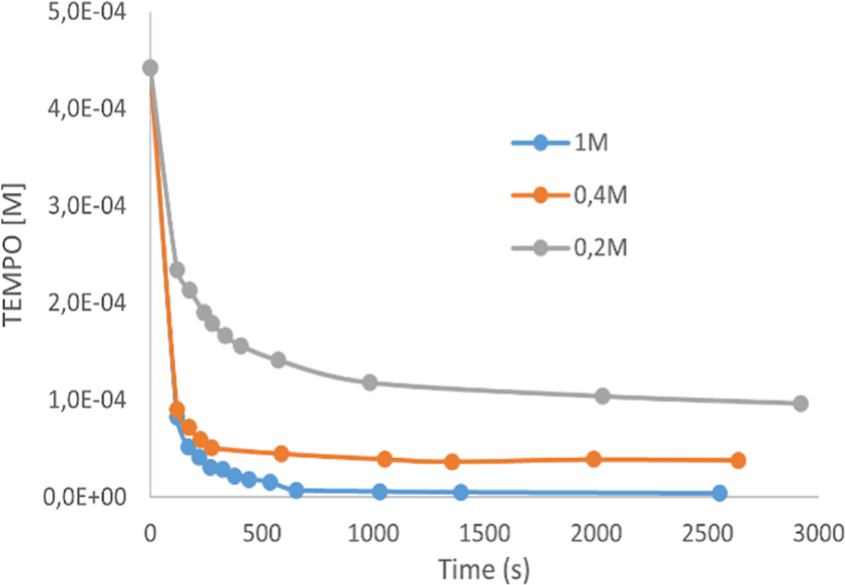
EPR analysis of time decay of the TEMPO radical in the presence of **2a** at 0.2, 0.4, and 1 M concentrations.

In the EPR experiment, the time-dependent behavior of the signal of the aminoxyl radical TEMPO in DCM in the presence of *N*-bromo-azetidinone **2a** at three different concentrations was monitored (Figure 3). The TEMPO radical disappeared rapidly in the presence of *N*-bromo-azetidinone **2a**, its EPR signal decayed exponentially in a **2a** concentration-dependent manner, and under these conditions, no regeneration of the aminoxyl radical was observed.

A tentative EPR experiment to detect an azetidinyl radical was conducted on **2a** in DCM with triethylsilane (TES) in the presence or absence of di-*t*-butyl peroxide, but the mixture resulted in great instability with sudden decomposition.

The reaction of *N*-bromo-azetidinone **2a** under optimized conditions with diphenyldisulfide was performed in DCM-*d*_2_ in an NMR tube, monitoring the ratio of **2a**:**1a**:**5** over time in a ^1^H NMR 400 MHz spectrum. *N*-Bromo-azetidinone **2a** was completely consumed in 5 h. The *N*-sulfenylated product **5** appeared immediately together with the NH derivative **1a**, and at complete conversion the composition of the crude mixture of **2a**:**1a**:**5** as 0:0.26:0.76 (Figure S1, Supporting Information). There was no evidence of deuterium exchange from the solvent, the signal of the NH appeared clearly in the spectra, and no deuterated species were detected in the mixture, so the reduced species **1a** could presumably derived from a hydrogen atom transfer (HAT) process by the highly reactive *N*-azetidinyl radical.

Thus, a further NMR investigation was realized to monitor the behavior of *N*-bromo-azetidinone **2a** in the presence of TEMPO at 20 mol % in DCM-*d*_2_ (Figure S2, Supporting Information).

The only product observed was the corresponding NH-azetidinone **1a** which, after the work up and within the experimental error in the integration, was recovered at around a 20% as the mol amount of TEMPO. It was also observed by ^1^H NMR analysis that the disulfide **3a** was stable over time in the presence of TEMPO (Figure S3, Supporting Information).

**Figure 4:**
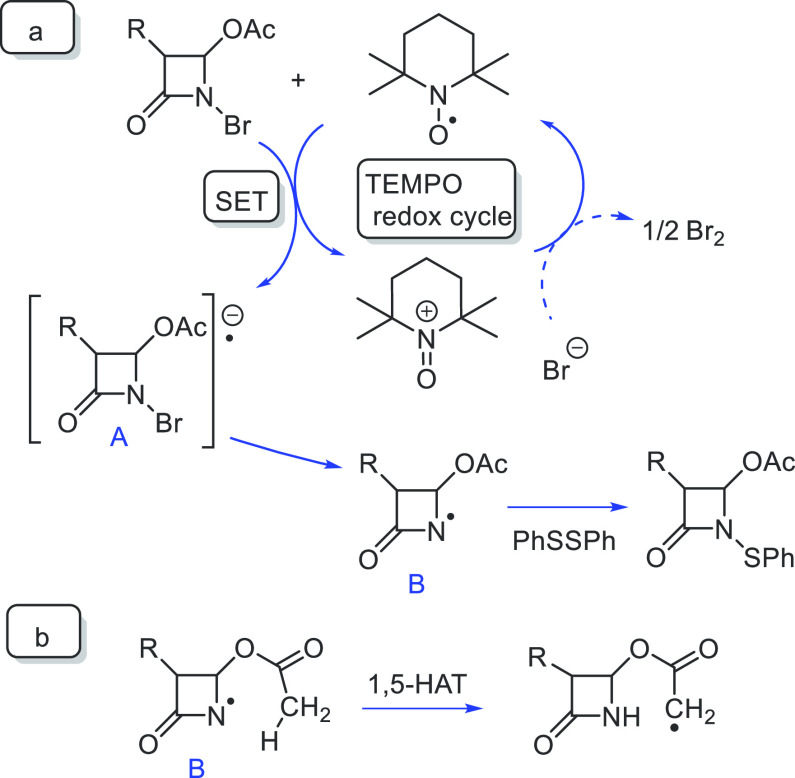
(a) Tentative reaction mechanism of the N-sulfenylation reaction of *N*-bromo-azetidinones; (b) 1,5-HAT process of azetidinyl radicals.

On considering the redox behavior of TEMPO that could give a reversible one-electron oxidation to the corresponding oxoammonium cation, a relative strong oxidant,^18–20^ a tentative hypothesis of the mechanism of the sulfenylation reaction could be formulated (Figure 4).

*N*-Bromo-azetidinones would be able to oxidize TEMPO, as evidenced by the EPR experiment, to give the oxammonium cation and the radical anion **A** by single-electron-transfer (SET). The highly reactive species **A** decomposes to azetidinyl radical **B** and the bromide anion. Amidyl radicals are highly reactive intermediates which can undergo some reactions as remote functionalization δ to nitrogen similar to a Hofmann–Löffler–Freytag reaction, cyclizations, or intermolecular additions.^21^ In our case, the amidyl radical **B** is quenched by the disulfide to give the desired sulfenylated product *N*-phenylthio-azetidinone. The formation of the byproduct NH azetidinone **1a** could be from a HAT process on the azetidinyl radical **B**. The hydrogen transfer could occur from the acetyl residue on the C-4 of **2a**, thus resulting in 1,5-HAT.^22^ The absence of NH-azetidinone as the byproduct in the sulfenylation reactions of *N*-bromo-azetidinones **14**, **15**, **16**, and **20** (Table 4), which have no 5 H atom, supports this hypothesis (Figure S4, Supporting Information). A restoration cycle for the TEMPO radical would be necessary, since only 20 mol % TEMPO is sufficient to give complete conversions and good yields (Table 2). The reaction conditions limit some possibilities; in particular, anaerobic conditions by inert atmosphere, aprotic reaction solvent, and the absence of H-donating species exclude the formation of *N*-hydroxy-TEMPO species.

The redox equilibrium between the oxoammonium salt and the nitroxyl radical in an electron self-exchange between the two species could then sustain the catalysis. This equilibrium, which is responsible for the paramagnetic character of oxoammonium salt in solutions, has been investigated in detail in the past by NMR and EPR.^23^ Traces of molecular bromine observed in the final reaction mixtures could have been derived from bromide oxidation by the oxoammonium cation, which was favored by the low concentrations of the species.

## Conclusions

In summary, a new N-sulfenylation reaction of azetidinones for the preparation of *N*-aryl-, or -alkylthio-β-lactam derivatives was established by an efficient redox catalysis by TEMPO. *N*-Bromo-azetidinones were able to oxidize the TEMPO radical for the generation of reactive azetidinyl radicals, which were further trapped by aryl- or alkyldisulfides to give the final *N*-sulfenylated azetidinones. The formation of *N*-halo-azetidinones was preliminary optimized as well as the next radical sulfenylation. The method exhibited a good substrate scope for either the starting *N*-bromo-azetidinones or the disulfides. This transformation presents not only a new radical reactivity of azetidinones but also a robust approach for the synthesis of bioactive *N*-sulfenyl-β-lactams. Moreover, the results reported here open the gate for further investigation on the chemistry of azetidinyl radicals.

## Experimental Section

### General Procedure for N-Halogenation (GP1) (Table 1): Synthesis of **2a** as an Example

In a round-bottom flask under a nitrogen atmosphere, the halogenating agent NBS (430 mg, 2.4 mmol, 1 equiv) was added at 0 °C to a solution of the starting β-lactam **1a** (310 mg, 2.4 mmol, 1 equiv) in anhydrous DCM (6 mL). The reaction was left under stirring at 0 °C and monitored by TLC. A second addition of NBS (110 mg, 0.6 mmol, 0.25 equiv) after 4 h allowed a complete conversion. The reaction was then quenched with water and extracted with DCM (3 × 20 mL). The collected organic phase was dried over Na_2_SO_4_, filtered, and the solvent removed under reduced pressure. The crude product was then purified by flash chromatography on silica gel (Cy/EtOAc = 70:30), and the product **2a** was isolated in 96% yield (478 mg).

### General Procedure for the Synthesis of **2a** on a 5.0 mmol Scale

In a round-bottom flask under a nitrogen atmosphere, NBS (890 mg, 5 mmol, 1 equiv) was added at 0 °C to a solution of the starting β-lactam **1a** (645 mg, 5 mmol, 1 equiv) in anhydrous DCM (12.5 mL). The reaction was left under stirring at 0 °C and monitored by TLC. A second addition of NBS (223 mg, 1.25 mmol, 0.25 equiv) after 4 h allowed a complete conversion. The reaction was then quenched with water and extracted with DCM (3 × 40 mL). The collected organic phase was dried over Na_2_SO_4_, filtered, and the solvent removed under reduced pressure. The crude product was then purified by flash chromatography on silica gel (Cy/EtOAc = 70:30), and the product **2a** was isolated in 89% yield (921 mg).

Caution! NBS is an irritating and sensitizing agent for skin and eyes (Category 2) and could cause skin burns and eye damage (H314 and H315, PubMed Source), handled with gloves in a normal fume-hood. It is very toxic to aquatic life, H400 (PubMed Source). The new *N*-bromo derivatives could be considered with hazard concerns similar to NBS and used with the same care.

### General Procedure for Thioalkylation/Thioarylation (GP2) (Tables 2–4): Synthesis of **5** as an Example

In a Schlenk flask under a nitrogen atmosphere, the selected *N*-bromo-azetidinone **2a** (41.5 mg, 0.2 mmol, 1 equiv) was diluted in 0.5 mL of anhydrous DCM; diphenyl disulfide (44 mg, 0.2 mmol, 1 equiv) was then added, followed by TEMPO (0.04 mmol, 0.2 equiv, 6.3 mg). The reaction was stirred at room temperature and monitored by TLC for 5 h. At completion, DCM was evaporated under reduced pressure, and the crude was purified by flash chromatography on silica gel (Cy/EtOAc = 70:30), yielding compound **5** as a colorless oil in 82% yield (39 mg).

### General Procedure for the Synthesis of **5** on a 5.0 mmol Scale

In a round-bottom flask under a nitrogen atmosphere, the selected *N*-bromo-azetidinone **2a** (1.040 g, 5 mmol, 1 equiv) was diluted in 12.5 mL of anhydrous DCM; the diphenyl disulfide (1.091 g, 5 mmol, 1 equiv) was then added, followed by TEMPO (1 mmol, 0.2 equiv, 0.156 g). The reaction was stirred at room temperature and monitored by TLC. After 5 h, DCM was evaporated under reduced pressure, and the crude was purified by flash chromatography on silica gel (*n*-hexane/EtOAc = 75:25), yielding compound **5** (1.033 g) as a colorless oil in 87% yield.

## Data Availability

The data underlying this study are available in the published article and its Supporting Information.

## Supporting Information Available

Detailed experimental procedures and characterization; copies of ^1^H and ^13^C NMR spectra; and HPLC–MS spectra of *N*-thio-β-lactams (compounds **5–21**) (PDF)
