# Supplementary material for: N-Sulfenylation of β-Lactams: Radical Reaction of N-Bromo-azetidinones by TEMPO Catalysis
Source: J Org Chem. 2023 Sep 28;88(20):14728–35. doi: 10.1021/acs.joc.3c01759 (PMC10594653; doi:10.1021/acs.joc.3c01759)
Supplement: Supplementary file 1 — jo3c01759_si_001.pdf [file jo3c01759_si_001.pdf]

N-Sulfenylation of β-Lactams: Radical Reaction of *N*-Bromo-azetidinones by TEMPO Catalysis

Valentina Giraldi,[a] Francesco Giunchino,[a] Maria Edith Casacchia,[a,b] Andrea Cantelli,[a] Marco Lucarini,[a] and Daria Giacomini*,[a]

Supporting Information

Table of Contents

Detailed experimental procedures and characterization……………………………………….S1

Figure S1, S2, S3, and S4……………………………………………………………………..S10

Copies of ^{1}H and ^{13}C NMR spectra …………………………………………………………..S14

HPLC-MS spectra of N-thio β-lactams (Compounds **5-21**)……………… …………………….S37

References……………………………………………………………………………………..S46

Detailed experimental procedures and characterization.

General methods

Commercial reagents and solvents were used further purification. Merck silica gel (240-400 mesh, 60 Å) was used for flash chromatography purifications. ^1H and ^{13}C NMR spectra were recorded with a Varian INOVA 400MHz instrument with a 5 mm probe. All chemical shifts were quoted relative to deuterated solvent signals (δ in ppm and J in Hz). Polarimetric analyses were conducted at 20°C on an Unipol L1000 "Schmidt−Haensch" polarimeter at 589.30 nm. ATR-FTIR spectra of pure compounds were recorded with a Bruker Alpha instrument and with an Agilent Technologies CARY 630 FTIR, in transmittance mode with a 4 cm^{-1} resolution in the 4000-400 cm-^{1} range. Melting points were found with a Buchi B-540 melting point Instrument. UPLC-MS analyses were performed with an Agilent Technologies 1260 Infinity II instrument, coupled with an Agilent Technologies Infinity Lab LC/MSD XT single-quadrupole mass spectrometer in full scan mode from m/z = 50 to 2600, in positive ion mode. The UPLC is equipped with a Phenomenex Gemini® 3 µm C18 (100x3 mm) column; the following method was used: mobile phase= H_2O/ACN (gradient from 30% to 80% of ACN in 8 minutes, then isocratic for 15 minutes), flow= 0.4 mL/min, temperature=40°C. GC-MS analyses, in EI ionization at 70 eV, were acquired on a Agilent Technologies 6950 Network GC System coupled with an Agilent 5975 Inert XL Mass Selective Detector, from 70°C to 230 °C in 20 minutes. High Resolution Mass Spectrometry analyses (HR-MS) were conducted on a Waters Acquity UPLC coupled with a Waters Xevo G2-XS QTof mass detector. ESR spectra were collected using a Bruker ELEXYS spectrometer equipped with an NMR gaussmeter for field calibration.
Compounds **1c**, **1d**, and **1e** were synthetized accordingly to already published procedures. [1,2]
Compounds **1f** was synthetized starting from **1a** in the presence of $Zn(OAc)_2$ and excess ethanol in toluene at 80°C. Compound **1g** was obtained from **1a**, in the presence of $NaHCO_3$ and phenol in a 3:2 acetone/water mixture, according to Ref. [3], whilst **1h** was obtained by reduction of **1b** with $NaBH_4$ at 0°C in ethanol [4].

Synthesis of 1-bromo-4-oxazetidin-2-yl acetate (2a)

Following GP1, starting from the commercially available 4-acetoxy azetidin-2-one **1a** (310 mg, 2.4 mmol, 1 eq) in DCM (6 mL), by using 1 equivalent of NBS (430 mg, 2.4, 1 eq) and a second addition of NBS (110 mg, 0.6 mmol, 0.25 eq) after 4 hours, **2a** was obtained, after flash chromatography on silica gel (Cy:EtOAc = 70:30), in 96% yield (478 mg, pale yellow oil which under high vacuum or by seed crystallization turns into a white solid). ^1H NMR (400 MHz, $CDCl_3$): δ 6.10 (dd, 1H, J = 4.1,

1.5 Hz), 3.44 (dd, 1H, J = 13.8, 4.2 Hz), 3.19 (dd, 1H, J = 13.8, 1.5 Hz), 2.1 (s, 3H). $^{13}C\{^1H\}$ NMR (100 MHz, $CDCl_3$): δ 170.0, 165.2, 80.0, 46.2, 20.9. GC-MS: Rt = 11.150 min, 148 $[M\text{-}CH_3COO]^+$ 90%, 121 $[M\text{-}CH_2CHOOCCH_3]^+$ 22%, 106 $[148\text{-}CH_2CO]^+$ 65%, 86 $[M\text{-}121]^+$ 100%. ATR-FTIR (cm^{-1}) 3024, 2961, 2929, 1752, 1726, 1374, 1348, 1321, 1216, 1198, 1172, 1037, 1017, 987, 888, 831, 505. Mp 64-65 °C.

Synthesis of (2*R*,3*R*)-1-bromo-3-((*R*)-1-((tert-butyldimethylsilyl)oxy)ethyl)-4-oxoazetidin-2-yl acetate (2b)

Following GP1, starting from the commercially available **1b** (172 mg, 0.6 mmol, 1 eq) in the presence of 2 equivalents of NBS (214 mg, 1.2 mmol) in anhydrous DCM (6 mL), compound **2b** was obtained in 3 hours as a pale yellow oil in 96% yield (210 mg), after flash chromatography on silica gel (Cy:EtOAc = 90:10). ^1H NMR (400 MHz, $CDCl_3$): δ 6.20 (d, 1H, J = 1.3 Hz), 4.24 (qd, 1H, J = 6.3, 2.7 Hz), 3.35 (dd, 1H, J = 2.8, 1.3 Hz), 2.16 (s, 3H), 1.25 (d, 3H, J = 6.3 Hz), 0.88 (s, 9H), 0.07 (d, 6H, J = 5.2 Hz). $^{13}C\{^1H\}$ NMR (100 MHz, $CDCl_3$): δ 169.9, 166.9, 82.3, 66.4, 64.2, 25.8, 22.3, 21.0, -4.2, -5.0. GC-MS: Rt = 18.608 min, 297 = $[M]^{+\cdot}$ 1%, 192 = $[M\text{-}C_7H_7O]^+$ 9%, 148 = $[192\text{-}CH_2O]^+$ 7%, 106 = $[148\text{-}CH_2CO]^+$ 41%, 91 = $[C_7H_7]^+$ 100%. ATR-FTIR (cm^{-1}) 2955, 2930, 2886, 2857, 1792, 1762, 1376, 1218, 1173, 1063, 1036, 827, 809, 777. Polarimetry: $[\alpha]_D^{20}$= -14 (*c* 1.6, CH_3OH).

Synthesis of benzyl 2-(1-bromo-4-oxazetidin-2-yl)acetate (2c)

Following GP1, starting from **1c** (110 mg, 0.5 mmol, 1 eq) in the presence of 2 equivalents of NBS (178 mg, 1 mmol) in anhydrous DCM (5 mL), compound **2b** was obtained in 5 hours as a pale yellow oil in 97% yield (144 mg), after flash chromatography on silica gel (Cy:EtOAc = 75:25). ^1H NMR (400 MHz, $CDCl_3$): δ 7.36 (t, 5H, J = 2.5 Hz), 5.19 (d, 1H, J_{AB}= 12.2 Hz), 5.15 (d, 1H, J_{AB}= 12.2 Hz), 4.12 (dtd, 1H, J = 7.9, 5.3, 2.6 Hz), 3.36 (dd, 1H, J = 13.5, 5.3 Hz), 3.02 (dd, 1H, J = 13.5, 2.6 Hz), 2.90 (dd, 1H, J = 16.3, 5.3 Hz), 2.62 (dd, 1H, J = 16.3, 8.0 Hz). $^{13}C\{^1H\}$ NMR (100 MHz, $CDCl_3$): δ 169.4, 167.0, 135.3, 128.8, 128.8, 128.6, 67.3, 55.0, 44.9, 37.9. GC-MS Rt = 18.608 min, 297 = $[M]^{+\cdot}$ 1%, 192 = $[M\text{-}C_7H_7O]^+$ 9%, 148 = $[192\text{-}CH_2O]^+$ 7%, 106 = $[148\text{-}CH_2CO]^+$ 41%, 91 = $[C_7H_7]^+$ 100%. ATR-FTIR (cm^{-1}) 3089, 3065, 3033, 2955, 1764, 1727, 1389, 1259, 1168, 1093, 1015, 738, 697.

Synthesis of 1-bromo-4-(phenylsulfonyl)azetidin-2-one (2d)

Following GP1, starting from **1d** (106 mg, 0.5 mmol) in the presence of 2 equivalents of NBS (178 mg, 1 mmol) in anhydrous DCM (5 mL), compound **2d** was obtained in 4 hours as a waxy white solid in 58% yield (84 mg) after flash chromatography on silica gel (Cy:EtOAc = 85:15). ^1H NMR (400 MHz, $CDCl_3$): δ 7.99 (ddd, 2H, J = 8.5, 1.3, 0.6 Hz), 7.82 – 7.73 (m, 1H), 7.66 (ddd, 2H, J = 8.6, 7.1,

0.6 Hz), 4.74 (dd, 1H, J = 5.3, 2.7 Hz), 3.49 (dd, 1H, J = 14.2, 2.7 Hz), 3.43 (dd, 1H, J = 14.2, 5.3 Hz). $^{13}C\{^1H\}$ NMR (100 MHz, $CDCl_3$): δ 164.7, 135.4, 129.9, 129.9, 129.5, 72.1, 42.2. ATR-FTIR (cm^{-1}) 1770, 1447, 1308, 1144, 1064, 745, 683, 562, 513, 408.

Synthesis of (3*S*,4*R*)-1-bromo-3-((*R*)-1-((tert-butyldimethylsilyl)oxy)ethyl)-4(phenylsulfonyl)azetidin2one (2e)

Following GP1, starting from **1e** (50 mg, 0.135 mmol, 1 eq) in the presence of 2 equivalents of NBS (48 mg, 0.27 mmol) in anhydrous DCM (1.3 mL), compound **2e** was obtained in 3 hours as a waxy colourless solid in 86% yield (70 mg), after flash chromatography on silica gel (Cy:EtOAc = 90:10). ^1H NMR (400 MHz, $CDCl_3$): δ 7.98 (dd, 2H, J = 8.4, 1.2 Hz), 7.79 – 7.71 (m, 1H), 7.68 – 7.60 (m, 2H), 4.81 (d, 1H, J = 2.3 Hz), 4.30 (qd, 1H, J = 6.3, 1.8 Hz), 3.67 (t, 1H, J = 2.0 Hz), 1.20 (d, 3H, J = 6.4 Hz), 0.84 (s, 9H), 0.06 (s, 3H), 0.03 (s, 3H). $^{13}C\{^1H\}$ NMR (100 MHz, $CDCl_3$): δ 167.2, 135.2, 129.8, 129.8, 74.3, 63.9, 63.5, 25.8, 22.4, 17.9, -4.2, -5.0. ATR-FTIR (cm^{-1}) 2955, 2930, 2885, 2857, 1787, 1447, 1325, 1254, 1140, 1057, 957, 838, 826, 778, 583. Polarimetry: $[\alpha]_D^{20}$= -79 (*c* 0.47, CH_3OH).

Synthesis of 1-bromo-4-ethoxyazetidin-2-one (2f)

Following GP1, starting from **1f** (100 mg, 0.87 mmol, 1 eq) in the presence of 2 equivalents of NBS (309 mg, 1.74 mmol) in anhydrous DCM (8.7 mL), compound **2f** was obtained in 1 hour as a yellow oil in 15% yield (24 mg), after flash chromatography on silica gel (Cy:EtOAc = 65:35). ^1H NMR (400 MHz, $CDCl_3$): δ 5.09 (dd, 1H, J = 4.2, 1.8 Hz), 3.86 (dq, 1H, J = 9.3, 7.0 Hz), 3.66 (dq, 1H, J = 9.3, 7.0 Hz), 3.18 (dd, 1H, J = 13.6, 4.1 Hz), 3.10 (dd, 1H, J = 13.6, 1.8 Hz), 1.24 (t, 3H, J = 7.1 Hz). $^{13}C\{^1H\}$ NMR (100 MHz, $CDCl_3$): δ 166.5, 87.4, 64.6, 45.3, 29.6, 28.7, 15.2. ATR-FTIR (cm^{-1}) 3151, 3077, 2792, 1770, 1683, 1362, 1292, 1176, 1100, 1064, 1003, 816, 635.

Synthesis of 1-bromo-4-phenoxyazetidin-2-one (2g)

Following GP1, starting from **1g** (51 mg, 0.31 mmol, 1 eq) in the presence of 1.1 equivalents of NBS (61 mg, 0.34 mmol) in anhydrous DCM (0.75 mL), compound **2g** was obtained in 1 hour as a yellow oil in 81% yield (75 mg), after flash chromatography on silica gel (Cy:EtOAc = 75:25). ^1H NMR (400 MHz, $CDCl_3$): δ 7.39 – 7.30 (m, 2H), 7.16 – 7.08 (m, 1H), 7.08 – 7.01 (m, 2H), 5.68 (dd, 1H, J = 4.0, 1.6 Hz), 3.45 (dd, 1H, J = 13.3, 3.7 Hz), 3.32 (dd, 1H, J = 13.5, 1.6 Hz). $^{13}C\{^1H\}$ NMR (100 MHz, $CDCl_3$): δ 165.6, 155.7, 129.9, 123.8, 117.5, 85.9, 46.6. ATR-FTIR (cm^{-1}) 2939, 2253, 1772, 1590, 1485, 1362, 1286, 1223, 1195, 1128, 1051, 1005, 960, 859, 823, 751, 690.

Synthesis of (*S*)-1-bromo-3-((*R*)-1-((tert-butyldimethylsilyl)oxy)ethyl)azetidin-2-one (2h)

Following GP1, starting from **1h** (100 mg, 0.44 mmol, 1 eq) in the presence of 1 equivalents of NBS (78 mg, 0.44 mmol) in anhydrous DCM (1.1 mL), compound **2h** was obtained in 2 hours as a white solid in 89% yield (120 mg), after flash chromatography on silica gel (Cy:EtOAc = 75:25). ^{1}H NMR (400 MHz, $CDCl_3$): δ 4.23 (qd, 1H, J = 6.2, 3.1 Hz), 3.62 – 3.56 (m, 1H), 3.49 (td, 1H, J = 4.9, 1.1 Hz), 3.44 (td, 1H, J = 4.7, 2.1 Hz), 1.15 (d, 3H, J = 6.3 Hz), 0.86 (s, 9H), 0.05 (s, 6H). $^{13}C\{^{1}H\}$ NMR (100 MHz, $CDCl_3$): δ 169.5, 64.8, 61.1, 49.2, 25.8, 22.6, 17.9, -4.2, -5.0. ATR-FTIR (cm^{-1}) 2954, 2928, 2894, 2857, 1772, 1750, 1716, 1465, 1407, 1375, 1251, 1196, 1068, 1005, 956, 837, 805, 777. Polarimetry: $[\alpha]_D^{20}$= - 40 (*c* 0.14, CH_2Cl_2).

Synthesis of 1-iodo-4-oxazetidin-2-yl acetate (3a)

Following GP1, starting from **1a** (120 mg, 0.9 mmol, 1 eq) in the presence of NIS (405 mg, 1.8 mmol, 2 eq) in anhydrous DCM (3 mL), **3a** was obtained, after 4 hours and flash chromatography on silica gel (Cy:EtOAc = 70:30), as a brown solid in 71% yield (163 mg). ^{1}H NMR (400 MHz, $CDCl_3$): δ 6.04 (dd, 1H, *J* = 4.0, 1.4 Hz), 3.44 (dd, 1H, *J* = 14.0, 4.0 Hz), 3.32 (dd, 1H, *J* = 13.9, 1.5 Hz), 2.16 (d, 3H, *J* = 0.9 Hz). $^{13}C\{^{1}H\}$ NMR (100 MHz, $CDCl_3$): δ 170.2, 169.0, 79.2, 46.1, 20.9. GC-MS: R_t = 9.531 min, 253 = $[M-H_2]^{+\cdot}$ (29%), 196 = $[M-CH_3COO]^{+}$ (27%), 195 = $[M-CH_3COOH]^{+\cdot}$ (47%), 128 = $[M-I]^{+\cdot}$ (100%), 127 = $I^{+\cdot}$ (47%). ATR-FTIR (cm^{-1}) 3012, 2981, 1747, 1720, 1216, 1199, 1182, 1038, 1017, 891, 498.

Synthesis of 1-chloro-4-oxazetidin-2-yl acetate (4a)

Following GP1, starting from **1a** (155 mg, 1.2 mmol, 1 eq) in the presence of NCS (643 mg, 4.8 mmol, 4 eq) in anhydrous DCM (4 mL), **4a** was obtained in 7 hours as a colourless liquid in 42% yield (82 mg), after flash chromatography on silica gel (Cy:EtOAc 70:30). ^{1}H NMR (400 MHz, $CDCl_3$): δ 6.17 (dd, 1H, J = 4.2, 1.4 Hz), 3.50 – 3.36 (m, 1H), 3.16 – 3.07 (m, 1H), 2.18 (s, 3H). $^{13}C\{^{1}H\}$ NMR (100 MHz, $CDCl_3$): δ 170.0 164.0, 80.3, 45.4, 20.8. GC-MS: Rt = 9.657 min, 163 = $[M]^{+\cdot}$ (4%), 121 = $[M-CH_2CO]^{+}\cdot$ (6%), 113 = $[M-CH_3Cl]$ (4%), 104 = $[M-CH_3COO]^{+}$ (100%), 76 = $[104-CO]^{+}$ (66%). ATR-FTIR (cm^{-1}) 3028, 2963, 2922, 2852, 1793, 1751, 1375, 1358, 1197, 1154, 1040, 1020, 890.

Synthesis of 4-oxo-1-(phenylthio)azetidin-2-yl acetate (5)

Following GP2, **2a** (41.5 mg, 0.2 mmol) was reacted with diphenyl disulfide (44 mg, 0.2 mmol) for 5 hours, yielding compound **5** as a colourless oil in 82% yield (39 mg) after flash chromatography on

silica gel (Cy:EtOAc = 70:30). Spectroscopic data is consistent with those found in literature [1]. See below for ^{1}H NMR and HPLC-MS analyses.

Synthesis of 1-(methylthio)-4-oxoazetidin-2-yl acetate (6)

Following GP2, **2a** (41.5 mg, 0.2 mmol) was reacted with dimethyl disulfide (18 µL, 0.2 mmol) for 5 hours. Compound **6** was obtained as a colourless oil in 75% yield (26 mg) after flash chromatography on silica gel (Cy:EtOAc = 70:30). Spectroscopic data is consistent with those found in literature [1]. See below for ^{1}H NMR and HPLC-MS analyses.

Synthesis of 1-(isopropylthio)-4-oxoazetidin-2-yl acetate (7)

Following GP2, **2a** (41.5 mg, 0.2 mmol) was reacted with Diisopropyl disulfide (31 µL, 0.2 mmol), overnight. Compound **7** was obtained as a colourless oil in 64% yield (26 mg) after flash chromatography on silica gel (Cy:EtOAc = 70:30). Spectroscopic data is consistent with those found in literature [5]. See below for ^{1}H NMR and HPLC-MS analyses.

Synthesis of 1-(propylthio)-4-oxoazetidin-2-yl acetate (8)

Following GP2, **2a** (41.5 mg, 0.2 mmol) was reacted with dipropyl disulfide (31 µL, 0.2 mmol), overnight. Compound **8** was obtained as a colourless oil in 74% yield (30 mg) after flash chromatography on silica gel (Cy:EtOAc = 70: 30). Spectroscopic data is consistent with those found in literature [5]. See below for ^{1}H NMR and HPLC-MS analyses.

Synthesis of 1-(benzylthio)-4-oxoazetidin-2-yl acetate (9)

Following GP2, compound **2a** (41.5 mg, 0.2 mmol) was reacted with dibenzyl disulfide (49 mg, 0.2 mmol, 1 eq), for 5 hours. Compound **9** was isolated after flash cromatography on silica gel (Cy:EtOAc = 70:30), in 72% yield (36 mg), as a colourless oil. Spectroscopic data is consistent with data found in literature [5]. See below for ^{1}H NMR and HPLC-MS analyses.

Synthesis of 1-(benzo[d]thiazol-2-ylthio)-4-oxoazetidin-2-yl acetate (10)

Following GP2, compound **2a** (41.5 mg, 0.2 mmol) was reacted with 2,2'-Dibenzothiazolyl Disulfide (66 mg, 0.2 mmol) in 1 mL of DCM, overnight. Compound **10** was isolated after flash chromatography on silica gel (Cy:EtOAc =70:30), in 70% yield (41 mg), as a white solid. Spectroscopic data is consistent with data found in literature [5]. See below for ^{1}H NMR and HPLC-MS analyses.

Synthesis of 1-((4-methoxyphenyl)thio)-4-oxoazetidin-2-yl acetate (11)

Following GP2, compound **2a** (41.5 mg, 0.2 mmol) was reacted with bis(4-methoxyphenyl) disulfide (55.6 mg, 0.2 mmol), for 5 hours. Compound **11** was isolated after flash cromatography on silica gel (Cy:EtOAc = 70:30), in 82% yield (44 mg), as a colourless oil. Spectroscopic data is consistent with data found in literature [5]. See below for ^{1}H NMR and HPLC-MS analyses.

Synthesis of 1-((4-nitrophenyl)thio)-4-oxoazetidin-2-yl acetate (12)

Following GP2, compound **2a** (41.5 mg, 0.2 mmol) was reacted with bis(4-nitrophenyl) disulfide (61.7 mg, 0.2 mmol) in 1 mL of DCM, overnight. Compound **12** was isolated after flash cromatography on silica gel (Cy:EtOAc = 70:30), in 55% yield (29 mg), as a yellow solid. ^{1}H NMR (400 MHz, $CDCl_3$): δ 8.23 – 8.11 (m, 2H), 7.47 – 7.34 (m, 2H), 6.27 (dd, 1H, J = 4.4, 1.8 Hz), 3.58 (dd, 1H, J = 15.7, 4.4 Hz), 3.26 (dd, 1H, J = 15.7, 1.8 Hz), 2.03 (s, 1H). $^{13}C\{^{1}H\}$ NMR (100 MHz, $CDCl_3$): δ 169.9, 167.7, 146.9, 146.1, 125.0, 124.5, 79.3, 46.7, 20.8. HPLC-MS (ESI^+) Rt = 6.478 min, 305.1 = $[M+Na]^+$, 321.0 = $[M+K]^+$, 346.0 = $[M+ACN+Na]^+$. ATR-FTIR (cm^{-1}) 3110, 3100, 3020, 2959, 2921, 2852, 1788, 1747, 1504, 1341, 122-2, 1208, 1046, 1031, 837, 793. Mp: 89.8-90.8 °C.

Synthesis of 4-oxo-1-(pyridin-2-ylthio)azetidin-2-yl acetate (13)

Following GP2, compound **2a** (42 mg, 0.2 mmol) was reacted with 2,2-dipyridyldisulfide (44 mg, 0.2 mmol) for 5 hours. Compound **13** was obtained as a colourless oil in 78% yield (37 mg), after flash chromatography on silica gel (Cy:EtOAc: 75:25). ^{1}H NMR (400 MHz, $CDCl_3$): δ 8.42 (ddd, 1H, J = 4.9, 1.9, 1.0 Hz), 7.62 – 7.51 (m, 1H), 7.15 (dt, 1H, J = 8.1, 1.0 Hz), 7.06 (ddd, 1H, J = 7.5, 4.9, 1.0 Hz), 6.33 (dd, 1H, J = 4.4, 1.7 Hz), 3.65 (dd, 1H, J = 15.5, 4.4 Hz), 3.20 (dd, 1H, J = 15.5, 1.8 Hz), 2.08 (s, 3H). $^{13}C\{^{1}H\}$ NMR (100 MHz, $CDCl_3$): δ 170.2, 168.4, 158.3, 149.9, 137.0, 121.12, 118.4, 79.6, 46.8, 20.9. HPLC-MS (ESI^+): Rt =3.978 min, 239.2= $[M+H]^+$, 261.0= $[M+Na]^+$, 498.9=$[2M+Na]^+$. ATR-FTIR (cm^{-1}) 1784, 1750, 1575, 1450, 1418, 1374, 1280, 1198, 1152, 1128, 1042, 1023, 898, 826, 759, 725. HRMS (ESI^+/Q-TOF) m/z: $[M+H]^+$ Calcd for $C_{10}H_{11}N_2O_3S$ 239.0490; Found 239.0479.

Synthesis of benzyl 2-(4-oxo-1-(phenylthio)azetidin-2-yl)acetate (14)

Following GP2, compound **2c** (60 mg, 0.2 mmol) was reacted with diphenyl disulfide (44 mg, 0.2 mmol), overnight. Compound **14** was obtained as a colourless oil in 92% yield (60 mg), after flash chromatography on silica gel (Cy:EtOAc: 75:25). ^{1}H NMR (400 MHz, $CDCl_3$): δ 7.40 – 7.22 (m, 10H), 5.09 (d, 1H, J_{AB}= 12.3 Hz), 5.07 (d, 1H, J_{AB}= 12.3 Hz), 4.12 (dtd, 1H, J = 8.2, 5.3, 2.8 Hz),

3.32 (dd, 1H, J = 15.2, 5.5 Hz), 2.98 – 2.88 (m, 2H), 2.55 (dd, 1H, J = 16.2, 8.4 Hz). $^{13}C\{^{1}H\}$ NMR (100 MHz, $CDCl_3$): δ 16.9, 136.8, 135.4, 129.4, 128.8, 128.6, 128.5, 128.4, 128.3, 67.0, 52.5, 44.7, 37.9. HPLC-MS (ESI^+): Rt = 8.831 min, 328.0 = $[M+H]^+$, 350.0 = $[M+Na]^+$, 677.0 = $[2M+Na]^+$. ATR-FTIR (cm^{-1}) 3061, 3033, 2954, 2922, 2852, 1764, 1730, 1581, 1262, 1161, 1091, 1020, 738, 690. HRMS (ESI^+/Q-TOF) m/z: $[M+H]^+$ Calcd for $C_{18}H_{18}NO_3S$ 328.1007; Found 328.0993.

Synthesis of benzyl 2-(4-oxo-1-(propylthio)azetidin-2-yl)acetate (15)

Following GP2, compound **2c** (60 mg, 0.2 mmol) was reacted with dipropyl disulfide (31 µL, 0.2 mmol), overnight. Compound **15** was obtained as a colourless oil in 88% yield (52 mg), after flash chromatography on silica gel (Cy:EtOAc: 75:25). ^{1}H NMR (400 MHz, $CDCl_3$): δ 7.42 – 7.29 (m, 5H), 5.18 (d, 1H, J_{AB}= 12.3 Hz), 5.14 (d, 1H, J_{AB}= 12.3 Hz), 4.04 (dtd, 1H, J = 8.1, 5.4, 2.8 Hz), 3.25 (dd, 1H, J = 15.1, 5.4 Hz), 2.95 (dd, 1H, J = 16.0, 5.3 Hz), 2.83 (dd, 1H, J = 15.1, 2.8 Hz), 2.74 – 2.64 (m, 1H), 2.67 – 2.52 (m, 2H), 1.62 (h, 2H, J = 7.3 Hz), 1.00 (t, 3H, J = 7.3 Hz). $^{13}C\{^{1}H\}$ NMR (100 MHz, $CDCl_3$): δ 170.4, 170.1, 135.4, 128.8, 128.6, 128.5, 67.0, 52.3, 44.4, 41.0, 38.1, 22.2, 13.2. HPLC-MS (ESI^+): Rt = 8.231 min 294.1=$[M+H]^+$, 316.0= $[M+Na]^+$, 609.0=$[2M+Na]^+$. ATR-FTIR (cm^{-1}) 2931, 2933, 2873,1760, 1729, 1497, 1455,1398, 1314, 1261, 1163, 1091, 1020, 738, 607, 579. HRMS (ESI^+/Q-TOF) m/z: $[M+H]^+$ Calcd for $C_{15}H_{20}NO_3S$ 294.1164; Found 294.1151.

Synthesis of benzyl 2-(4-oxo-1-(benzylthio)azetidin-2-yl)acetate (16)

Following GP2, compound **2c** (60 mg, 0.2 mmol) was reacted with dibenzyl disulfide (49 mg, 0.2 mmol), overnight. Compound **16** was obtained as a colourless oil in 65% yield (44 mg), after flash chromatography on silica gel (Cy:EtOAc: 75:25). ^{1}H NMR (400 MHz, $CDCl_3$): δ 7.50 – 7.05 (m, 10 H), 5.09 (d, 1H, J_{AB}= 12.3 Hz), 5.05 (d, 1H, J_{AB}= 12.3 Hz), 4.04 (d, 1H, J = 12.6 Hz), 3.89 (d, 1H, J = 12.6 Hz), 3.49 (dtd, 1H, J = 8.2, 5.3, 2.8 Hz), 3.09 (dd, 1H, J = 15.1, 5.4 Hz), 2.80 – 2.58 (m, 2H), 2.30 (dd, 1H, J = 16.0, 8.3 Hz). $^{13}C\{^{1}H\}$ NMR (100 MHz, $CDCl_3$) δ (ppm) 169.9, 135.8, 135.5, 129.5, 128.9, 128.7, 128.6, 128.4, 127.9, 66.8, 52.3, 44.3, 42.9, 37.6, 27.0. HPLC-MS (ESI^+): Rt = 8.855 min, 342.0= $[M+H]^+$, 364.0=$[M+Na]^+$, 380.0= $[M+K]^+$, 705.0=$[2M+Na]^+$. ATR-FTIR (cm^{-1}) 3030, 2953, 2854, 1759, 1728, 1495, 1454, 1388, 1313, 1262, 1162, 1092, 1020, 739, 696, 506. HRMS (ESI^+/Q-TOF) m/z: $[M+H]^+$ Calcd for $C_{19}H_{20}NO_3S$ 342.1164; Found 342.1154.

Synthesis of (2*R*,3*R*)-3-((*R*)-1-((tert-butyldimethylsilyl)oxy)ethyl)-4-oxo-1-(phenylthio)azetidin-2-yl acetate (17)

Following GP2, compound **2b** (73 mg, 0.2 mmol) was reacted with diphenyl disulfide (44 mg, 0.2 mmol), overnight. Compound **17** was isolated as a colourless oil in 76% yield (60 mg), after flash

chromatography on silica gel (Cy:EtOAc = 90:10.) Spectroscopic data is consistent with data found in literature [1]. See below for ^{1}H NMR and HPLC-MS analyses.

Synthesis of (2*R*,3*R*)-3-((*R*)-1-((tert-butyldimethylsilyl)oxy)ethyl)-4-oxo-1-(propylthio)azetidin-2-yl acetate (18) Following GP2, compound **2b** (73 mg, 0.2 mmol) was reacted with dipropyl disulfide (31 μL, 0.2 mmol), overnight. Compound **18** was obtained as a colourless oil in 40% yield (29 mg), after flash chromatography on silica gel (Cy:EtOAc= 90:10). Spectroscopic data is consistent with data found in literature [1]. See below for ^{1}H NMR and HPLC-MS analyses.

Synthesis of (2*R*,3*R*)-1-(benzylthio)-3-((*R*)-1-((tert-butyldimethylsilyl)oxy)ethyl)-4-oxoazetidin-2-yl acetate (19) Following GP2, compound **2b** (73 mg, 0.2 mmol) was reacted with dibenzyl disulfide (49 mg, 0.2 mmol), overnight. Compound **19** was obtained as a colourless oil in 70% yield (57 mg), after flash chromatography on silica gel (Cy:EtOAc = 90:10). Spectroscopic data is consistent with data found in literature [1]. See below for ^{1}H NMR and HPLC-MS analyses.

Synthesis of (*S*)-3-((*R*)-1-((tert-butyldimethylsilyl)oxy)ethyl)-1-(phenylthio)azetidin-2-one (20)
Following GP2, compound **2h** (62 mg, 0.2 mmol) was reacted with diphenyl disulfide (44 mg, 0.2 mmol), overnight. Compound **20** was obtained as a colourless oil in 86% yield (58 mg), after flash chromatography on silica gel (Cy:EtOAc = 90:10). ^{1}H NMR (400 MHz, $CDCl_3$): δ 7.27 (d, 4H, J = 4.3 Hz), 7.22 – 7.14 (m, 1H), 4.20 (qd, 1H, J = 6.2, 4.1 Hz), 3.54 (dd, 1H, J = 5.3, 3.1 Hz), 3.43 (t, 1H, J = 5.5 Hz), 3.32 (ddd, 1H, J = 5.7, 4.2, 3.1 Hz), 1.13 (d, 3H, J = 6.2 Hz), 0.79 (s, 9H), 0.01 (s, 3H), -0.01 (s, 3H). ^{13}C{^{1}H} NMR (100 MHz, $CDCl_3$): δ 171.8, 137.1, 129.3, 127.6, 127.1, 65.2, 59.9, 46.3, 25.8, 22.7, 18.0, -4.4, -4.8. HPLC-MS (ESI$^+$): Rt=10.650 min, 337.2=[M+H]$^+$, 360.2=[M+Na]$^+$, 697.2= [2M+Na]$^+$. ATR-FTIR (cm^{-1}) 3064, 2956, 2930, 2892, 2957, 1767, 1582, 1472, 1441, 1374, 1308, 1290, 1252, 1165, 1139, 1109, 1083, 1060, 1005, 958, 837, 807, 777, 736, 688. Polarimetry: $[\alpha]_D^{20}$= -45 (*c* 0.36, CH_2Cl_2). HRMS (ESI$^+$/Q-TOF) m/z: [M+H]$^+$ Calcd for $C_{17}H_{28}NO_2SSi$ 338.1610; Found 338.1599.

Synthesis of 4-phenoxy-1-(phenylthio)azetidin-2-one (21)
Following GP2, compound **2g** (27mg, 0.11 mmol) was reacted with diphenyl disulfide (24 mg, 0.11 mmol), overnight. Compound **21** was obtained as a yellow oil in 40% yield (12 mg), after flash chromatography on silica gel (Cy:EtOAc = 90:10). ^{1}H NMR (400 MHz, $CDCl_3$): δ 7.46 – 7.21 (m, 7H), 7.05 (t, 1H, J = 7.4 Hz), 6.97 (d, 2H, J = 8.0 Hz), 5.73 (q, 1H, J = 2.0 Hz), 3.48 (dt, 1H, J = 15.0, 2.5 Hz), 3.25 (d, 1H, J = 15.1 Hz). ^{13}C{^{1}H} NMR (100 MHz, $CDCl_3$): δ 168.6, 156.4, 136.7, 129.8,

129.3, 128.4, 128.3, 123.1, 116.7, 84.4, 47.2. HPLC-MS (ESI$^+$): Rt=6.973min, 272.2=[M+H]$^+$, 294.0=[M+Na]$^+$, [2M+Na]$^+$, 335.0=[M+ACN+Na]$^+$, 565.0=[2M+Na]$^+$. ATR-FTIR (cm^{-1}) 3058, 2954, 2371, 1774, 1588, 1489, 1457, 1441, 1362, 1282, 1221, 1155, 1154, 1131, 1077, 1055, 1014, 978, 865, 738, 686. HRMS (ESI$^+$/Q-TOF) m/z: [M+H]$^+$ Calcd for $C_{15}H_{14}NO_2S$ 272.0745; Found 272.0734.

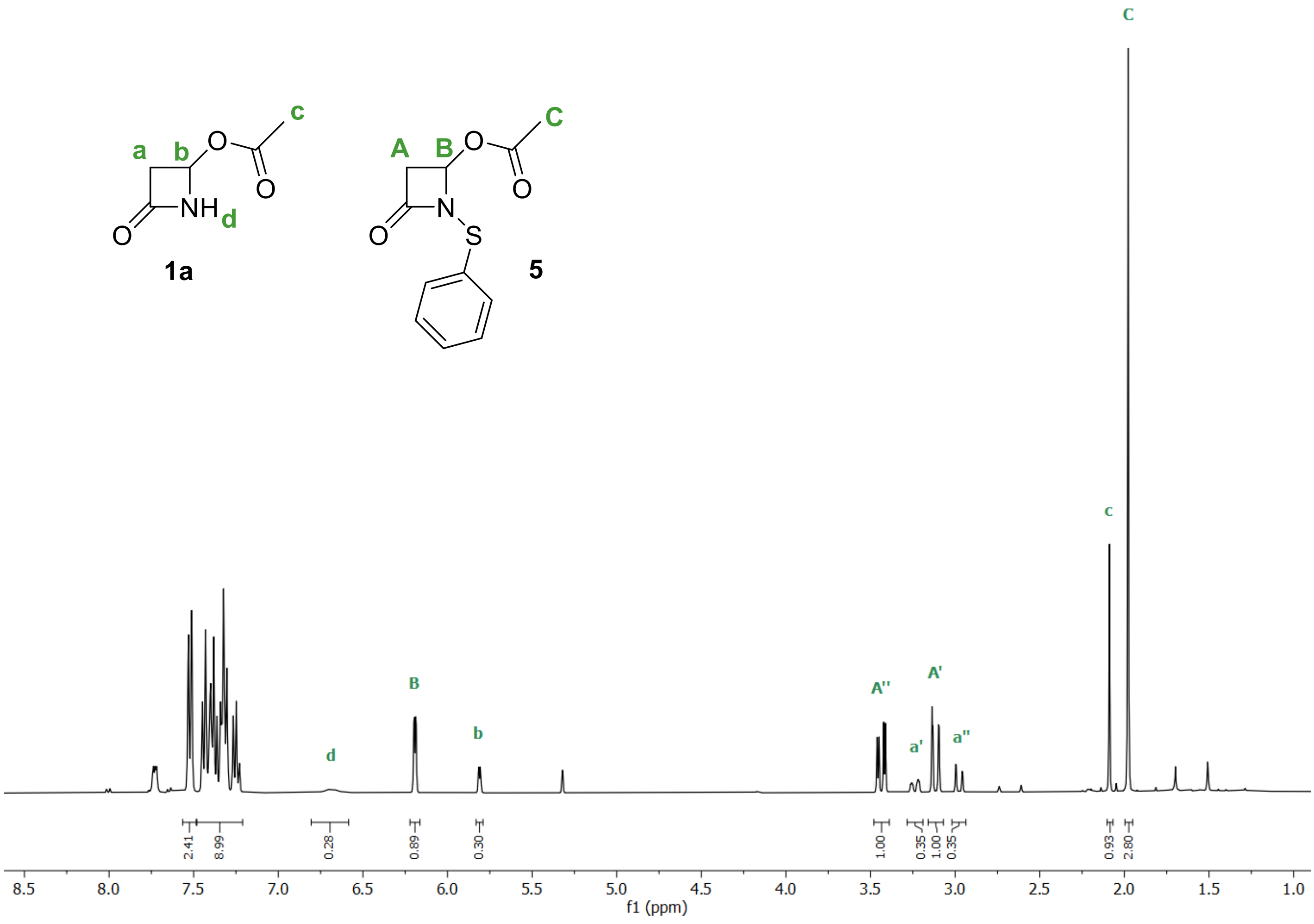

Figure S1: ^{1}H NMR ($CDCl_3$, 400 MHz) analysis of the crude mixture of N-sulfenylation of **2a** after work up by evaporation, showing the presence of the byproduct **1a** and the product **5.**

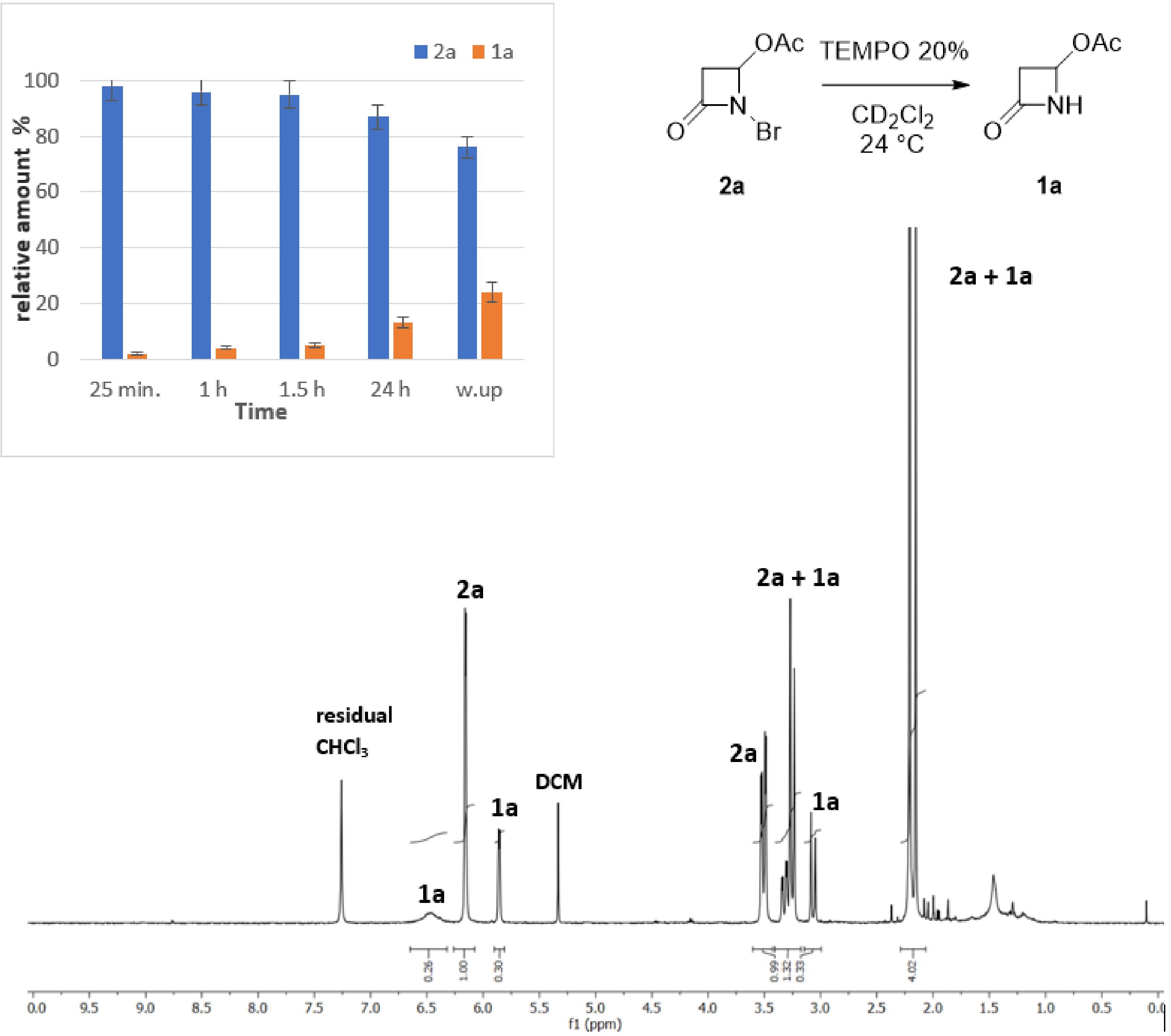

Figure S2. Time course study of ^{1}H NMR of **2a** and TEMPO in DCM-d2. The ^{1}H NMR spectra in $CDCl_3$ refers to the crude mixture after the work-up.

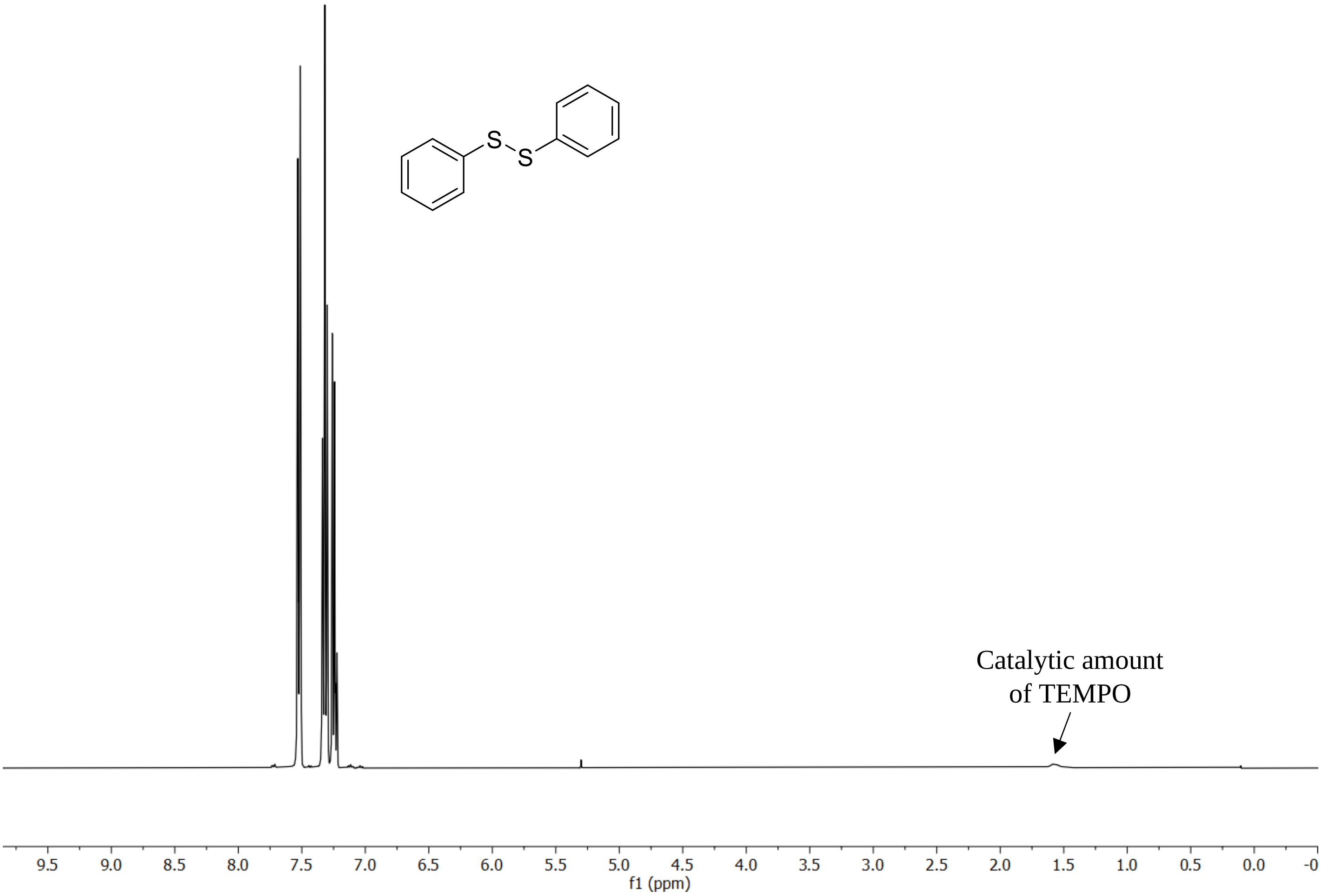

Figure S3. ^{1}H NMR ($CDCl_3$, 400 MHz) of diphenyl disulfide in the presence of TEMPO (20% mol), showing the stability of the disulfide after 24 hours.

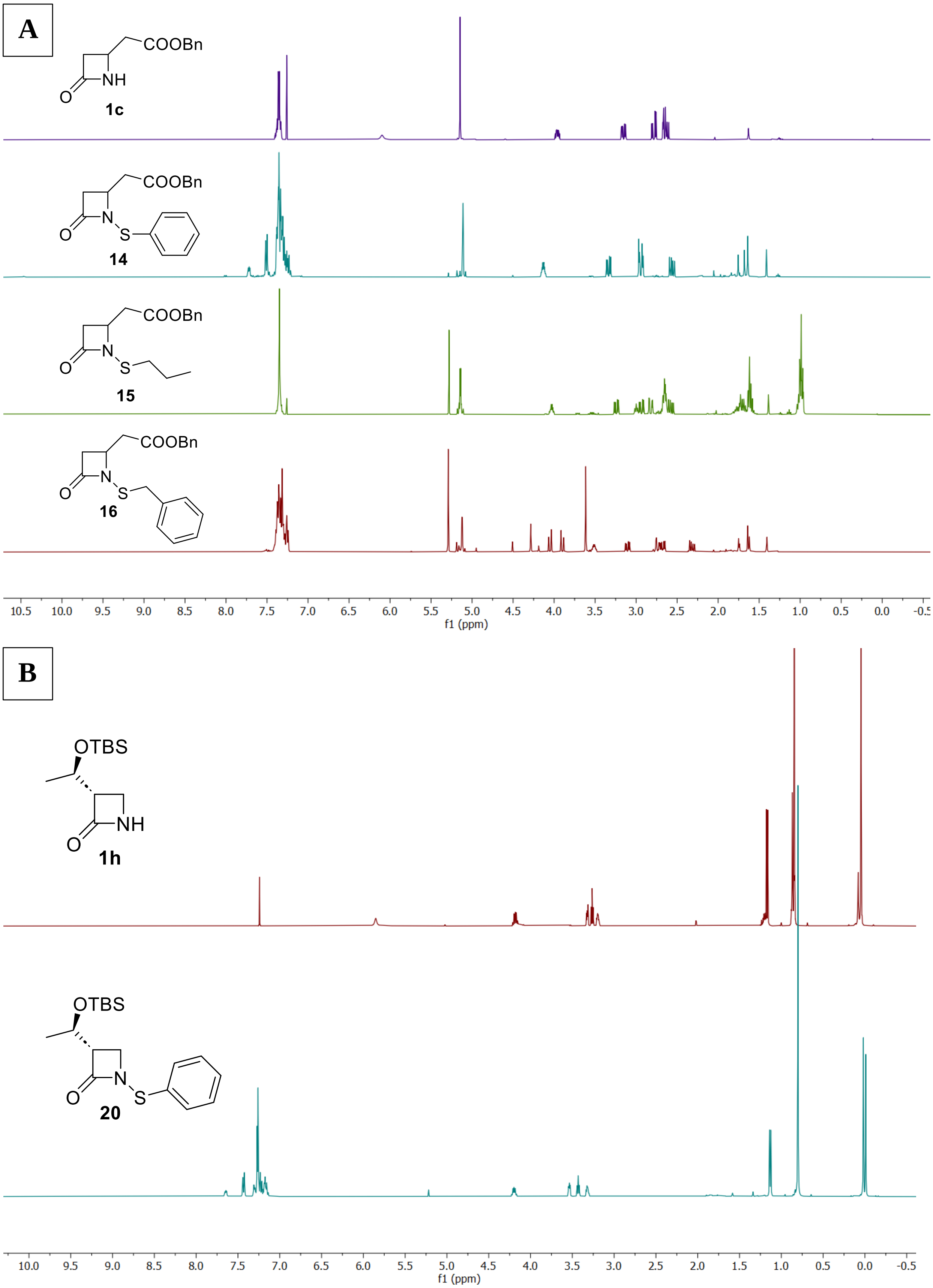

Figure S4. A) Comparison of ^{1}H NMR spectra of **1c** (purple) and the crude mixtures of N-sulfenylation of **2c** to obtain compounds **14** (blue), **15** (green) and **16** (red), showing the absence of **1c** as by-product. B) Comparison of ^{1}H NMR spectra of **1h** (red) and crude mixture of N-sulfenylation related to compound **20** (blue).

Copies of ^{1}H and $^{13}C\{^{1}H\}$ NMR spectra

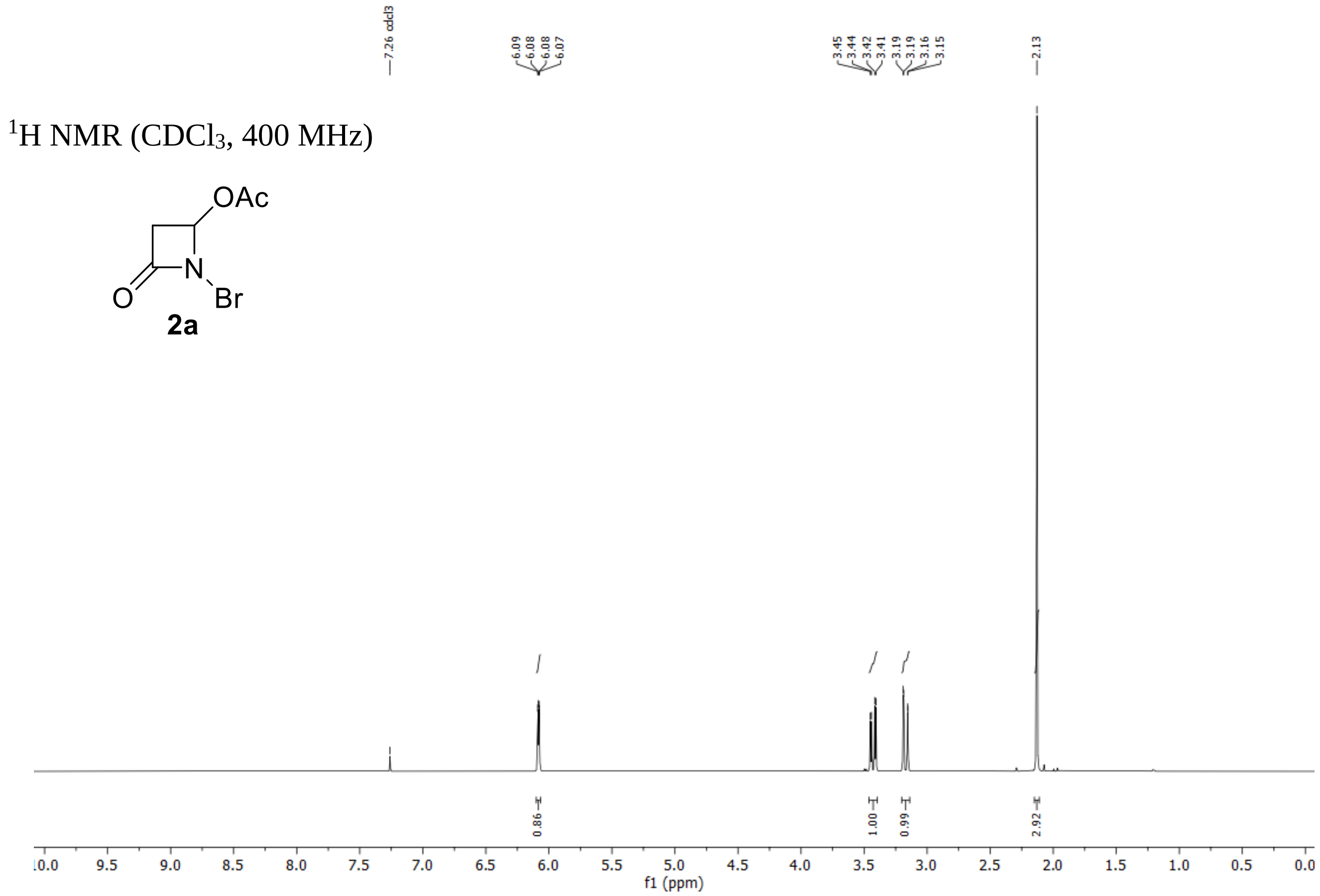

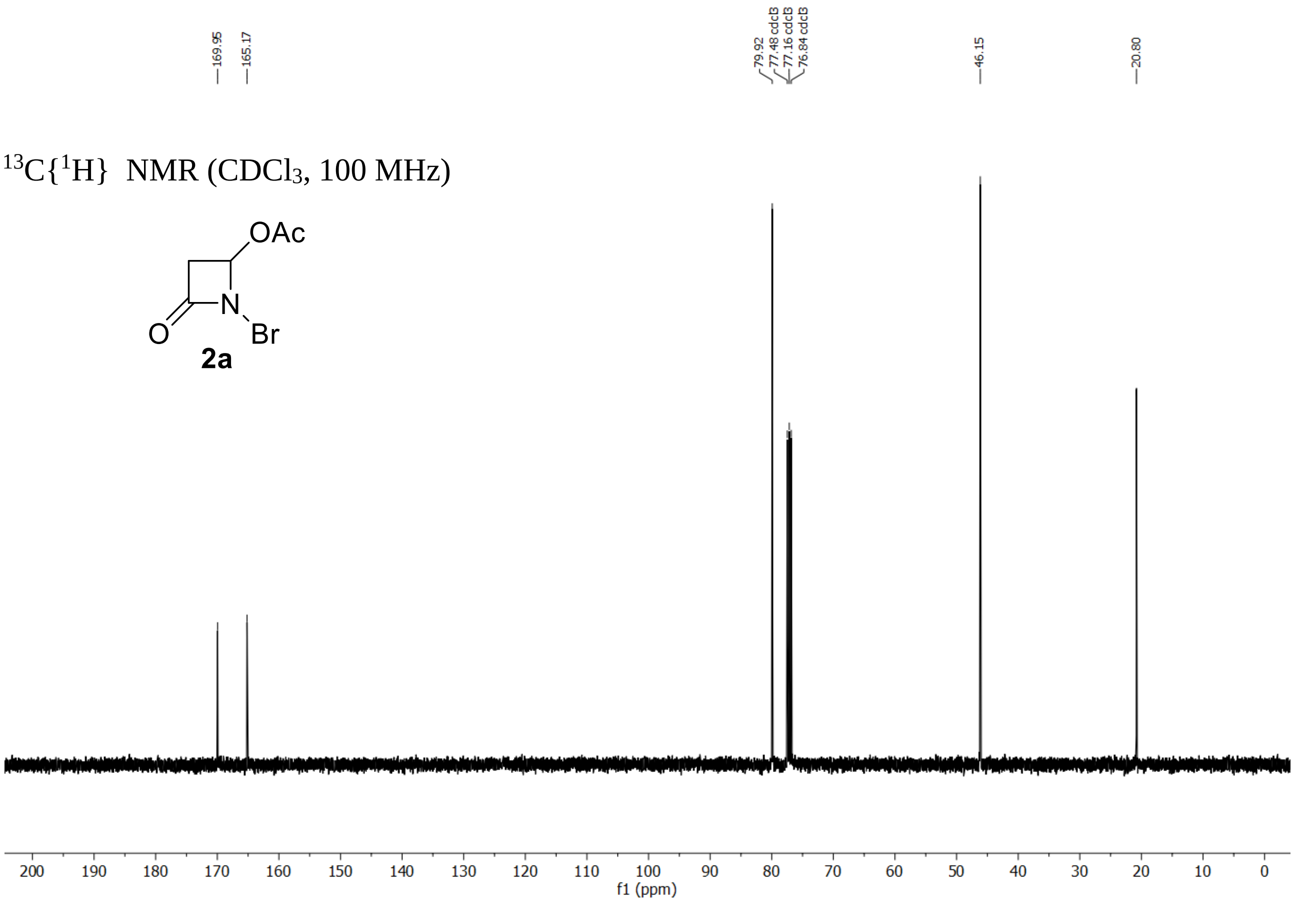

^{1}H NMR ($CDCl_3$, 400 MHz)

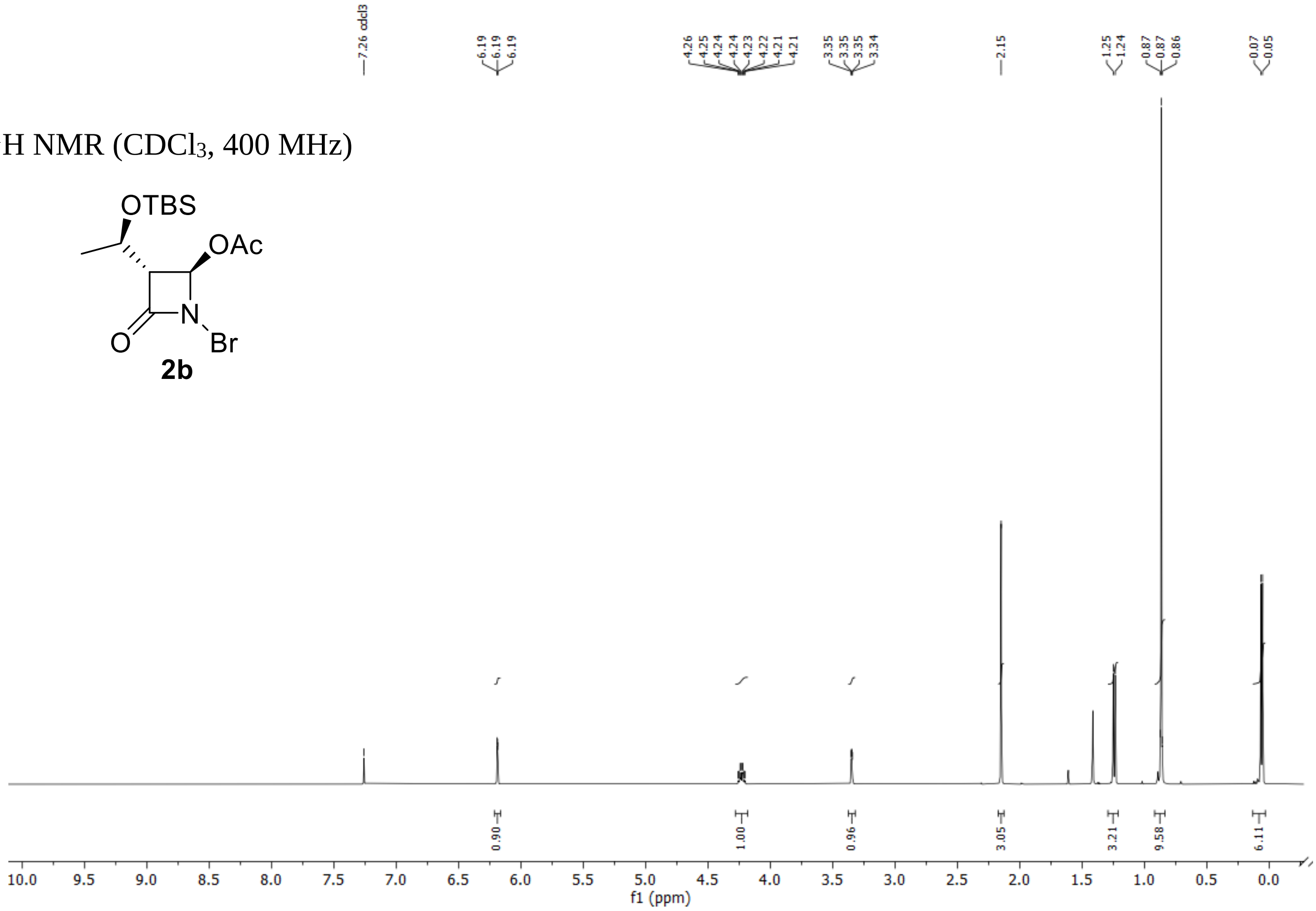

^{13}C{^{1}H} NMR ($CDCl_3$, 100 MHz)

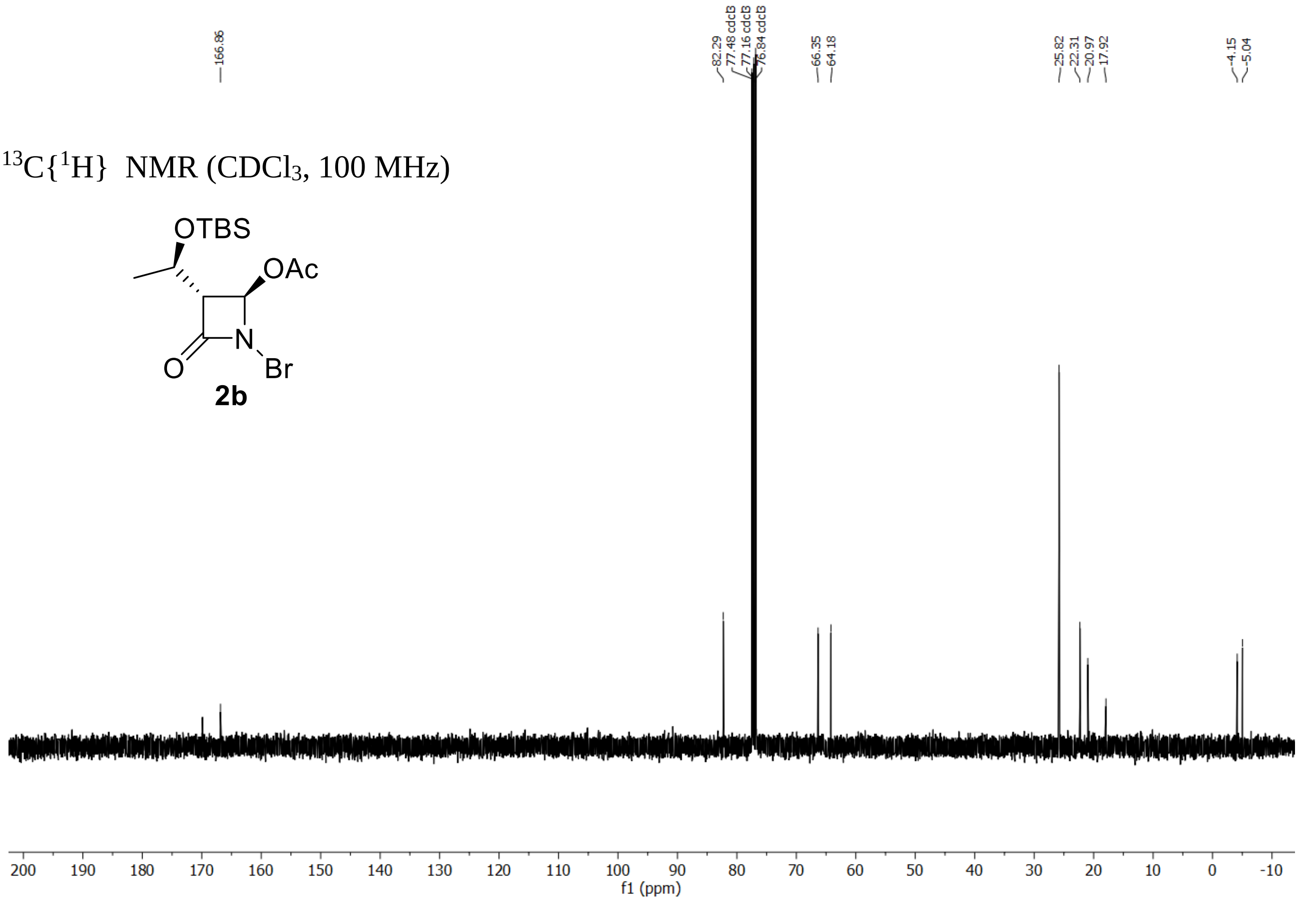

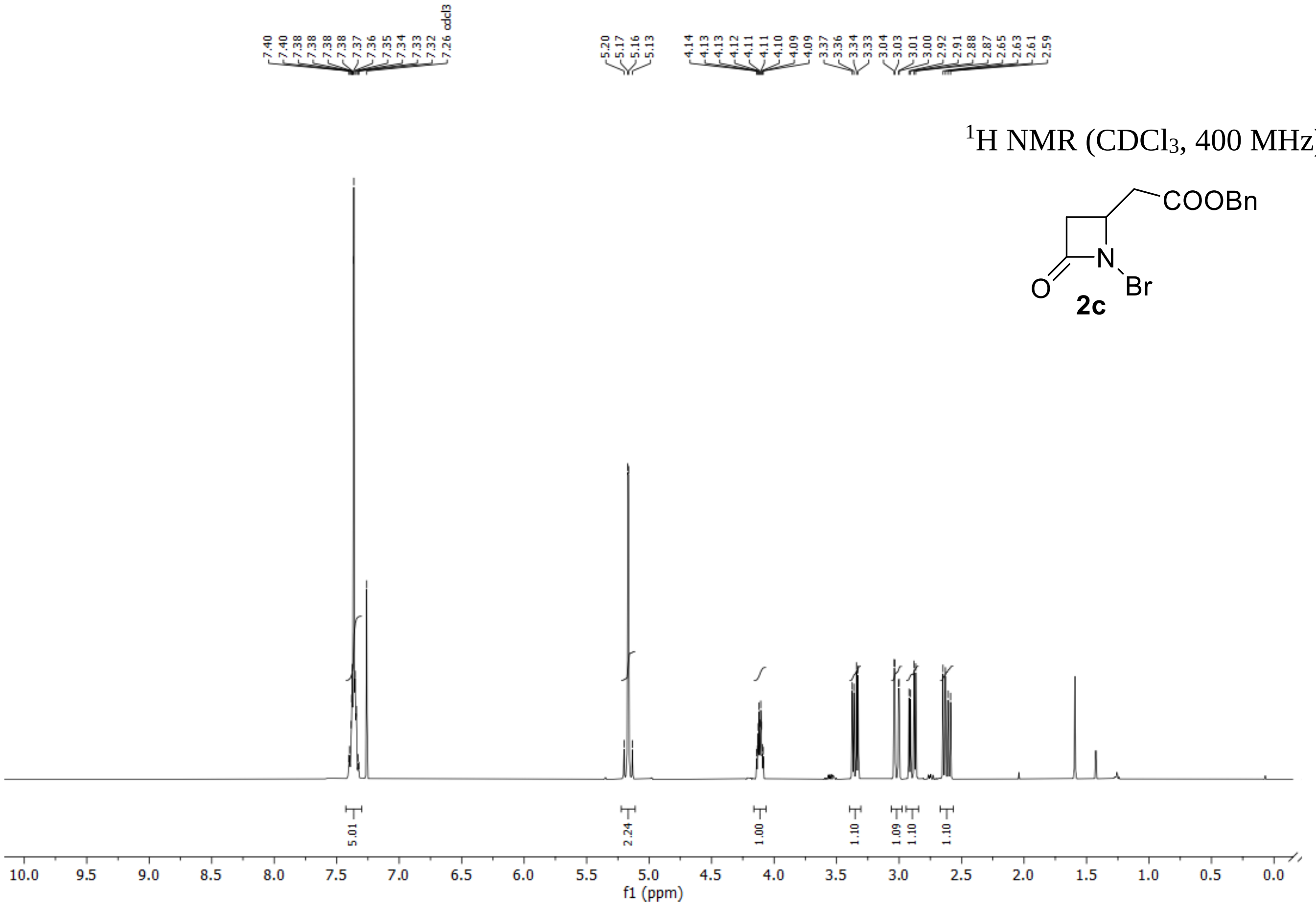

^{1}H NMR ($CDCl_3$, 400 MHz)

2c

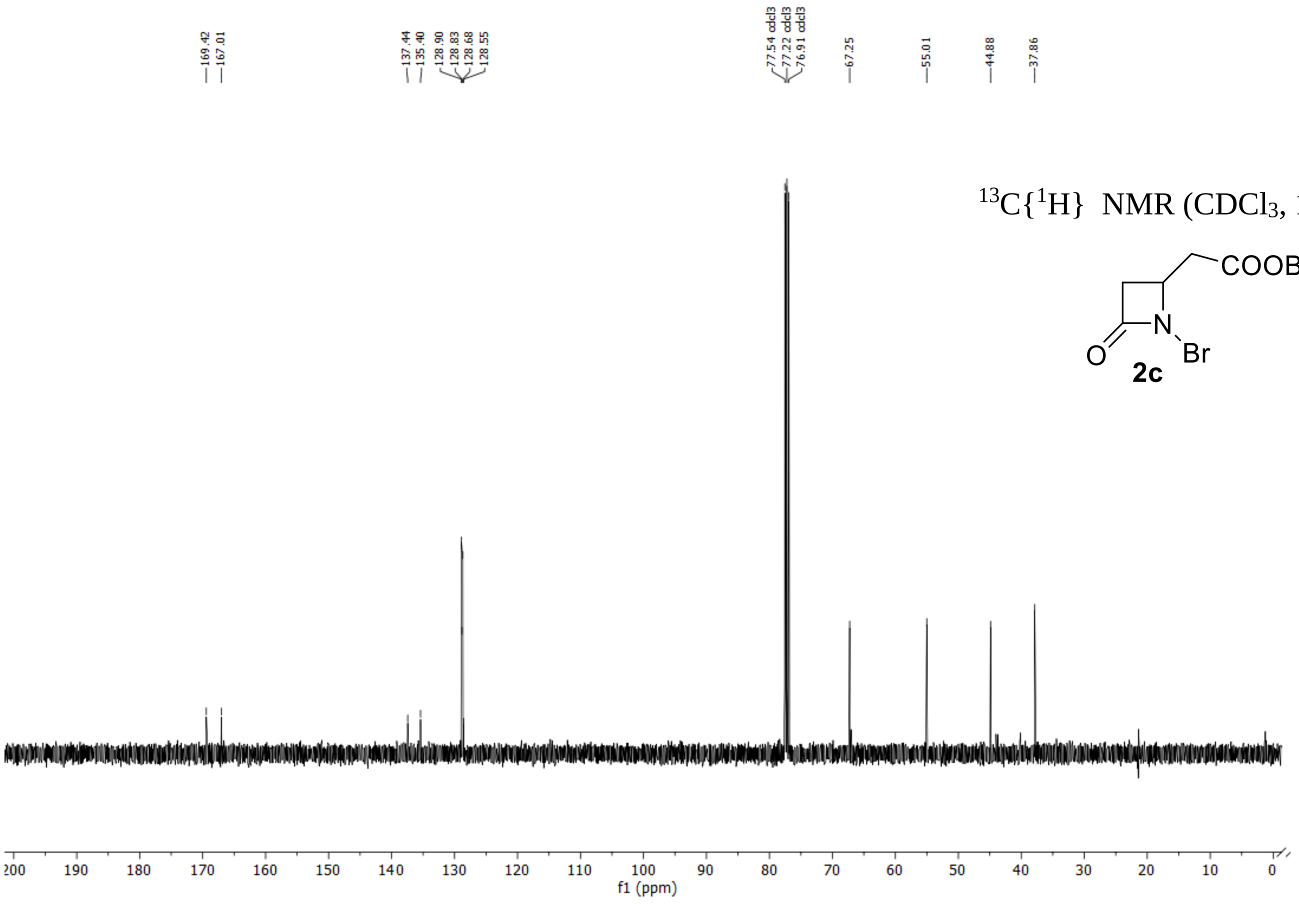

$^{13}C\{^{1}H\}$ NMR ($CDCl_3$, 100 MHz)

2c

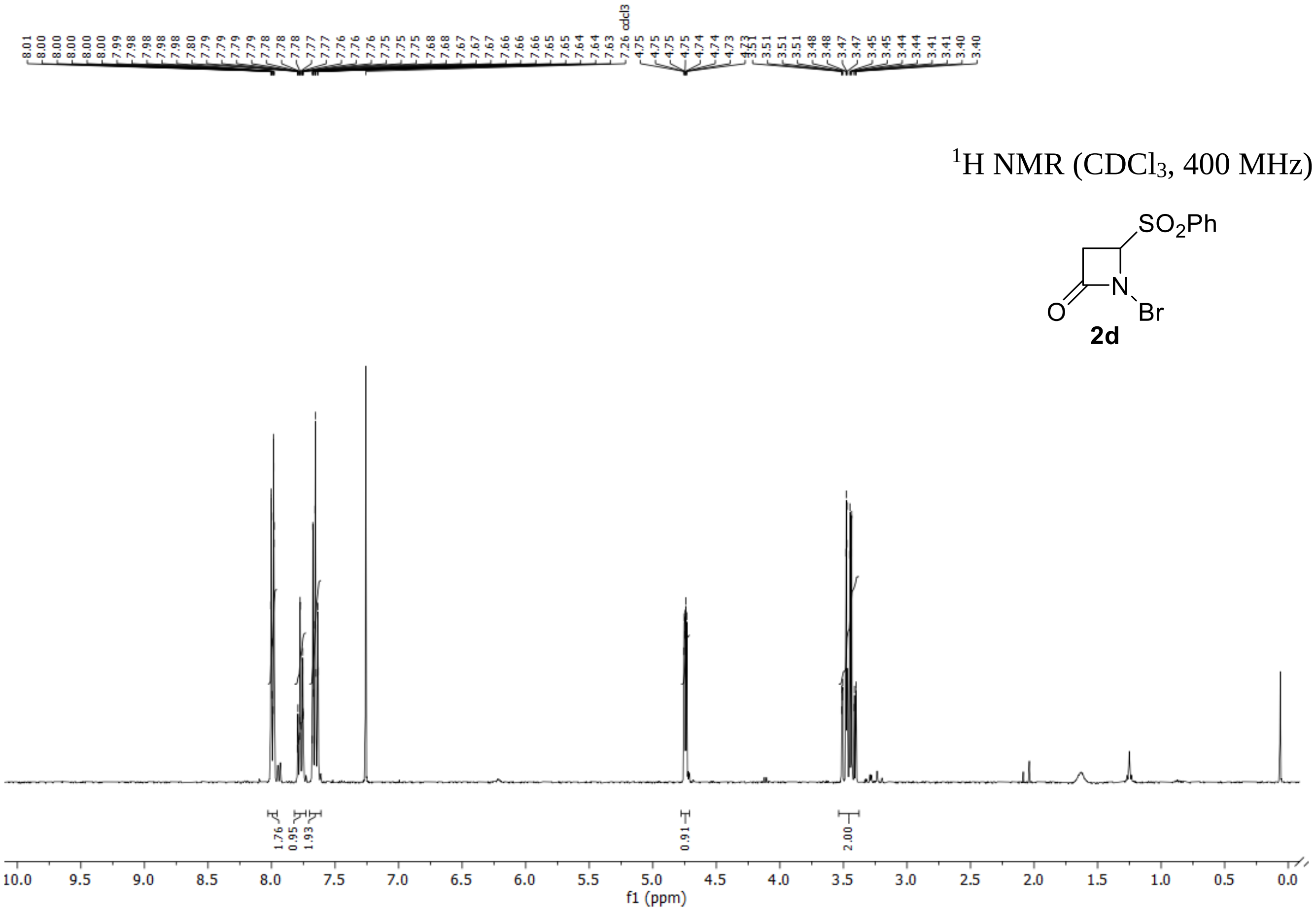

^{1}H NMR (CDCl$_3$, 400 MHz)
SO$_2$Ph
N
Br
O
2d
1.76
0.95
1.93
0.91
2.00
f1 (ppm)

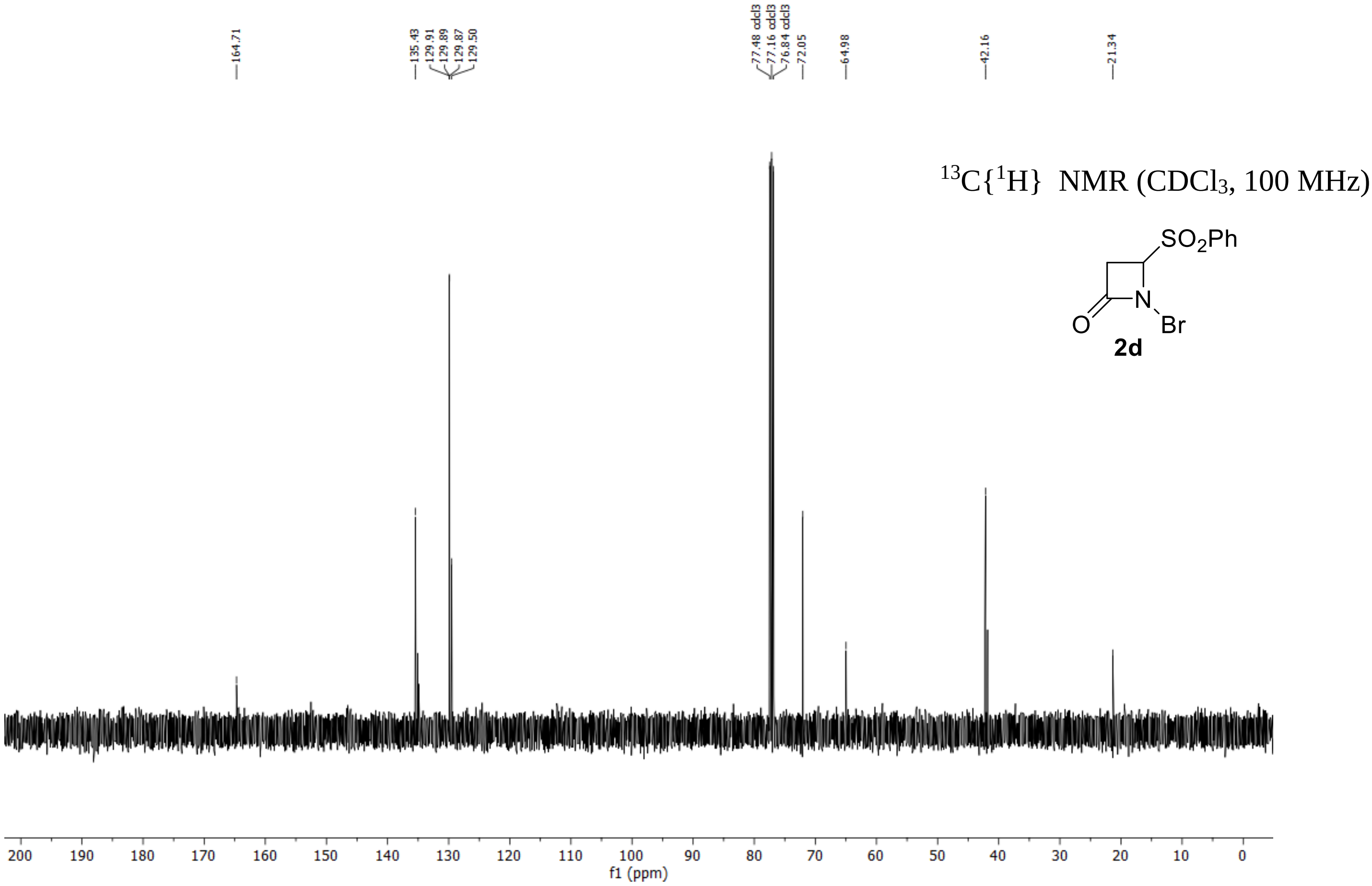

164.71
135.43
129.91
129.89
129.87
129.50
77.48 cdcl3
77.16 cdcl3
76.84 cdcl3
72.05
64.98
42.16
21.34
^{13}C{^{1}H} NMR (CDCl$_3$, 100 MHz)
SO$_2$Ph
N
Br
O
2d
f1 (ppm)

^{1}H NMR ($CDCl_3$, 400 MHz)

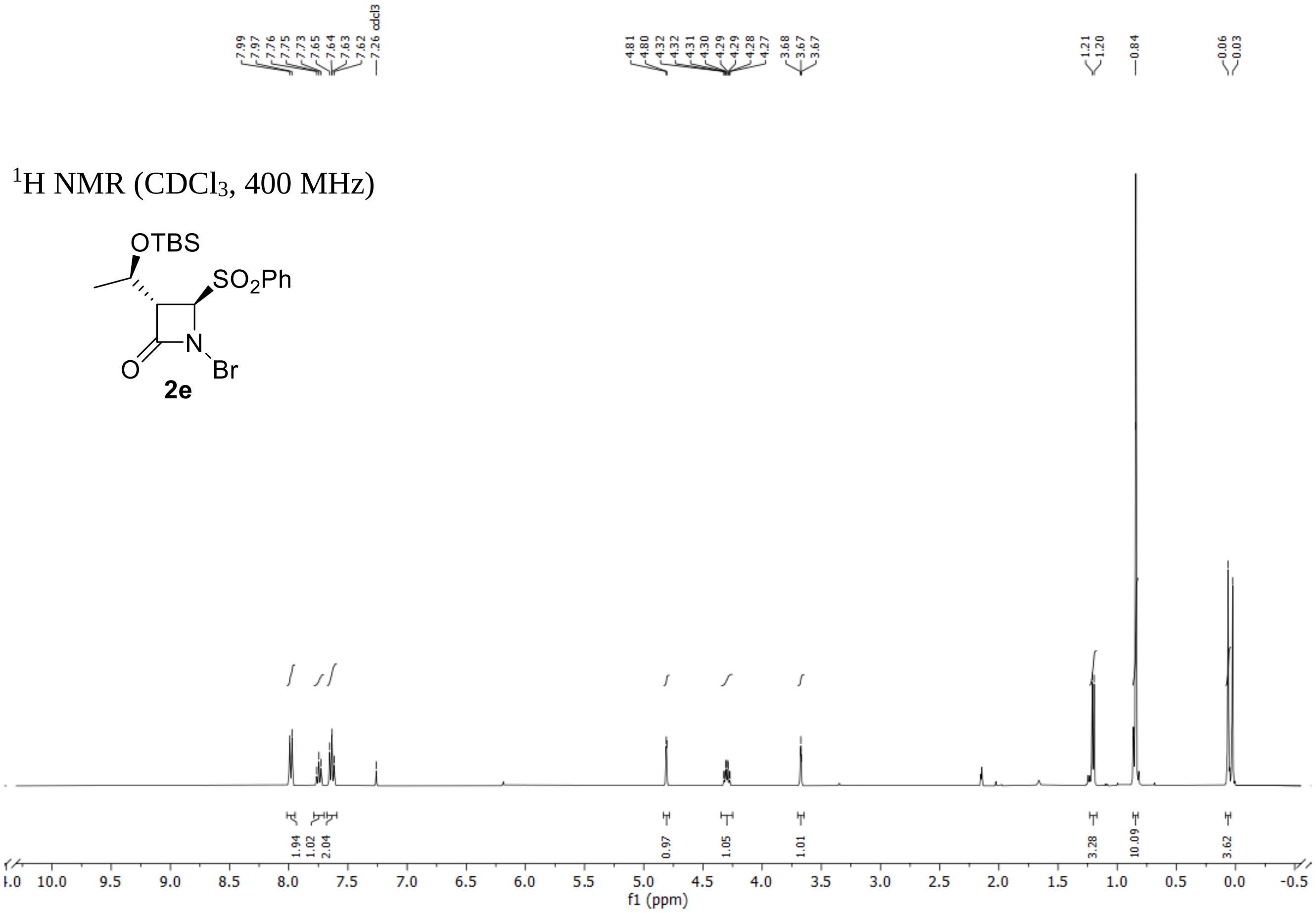

2e

^{13}C{^{1}H} NMR ($CDCl_3$, 100 MHz)

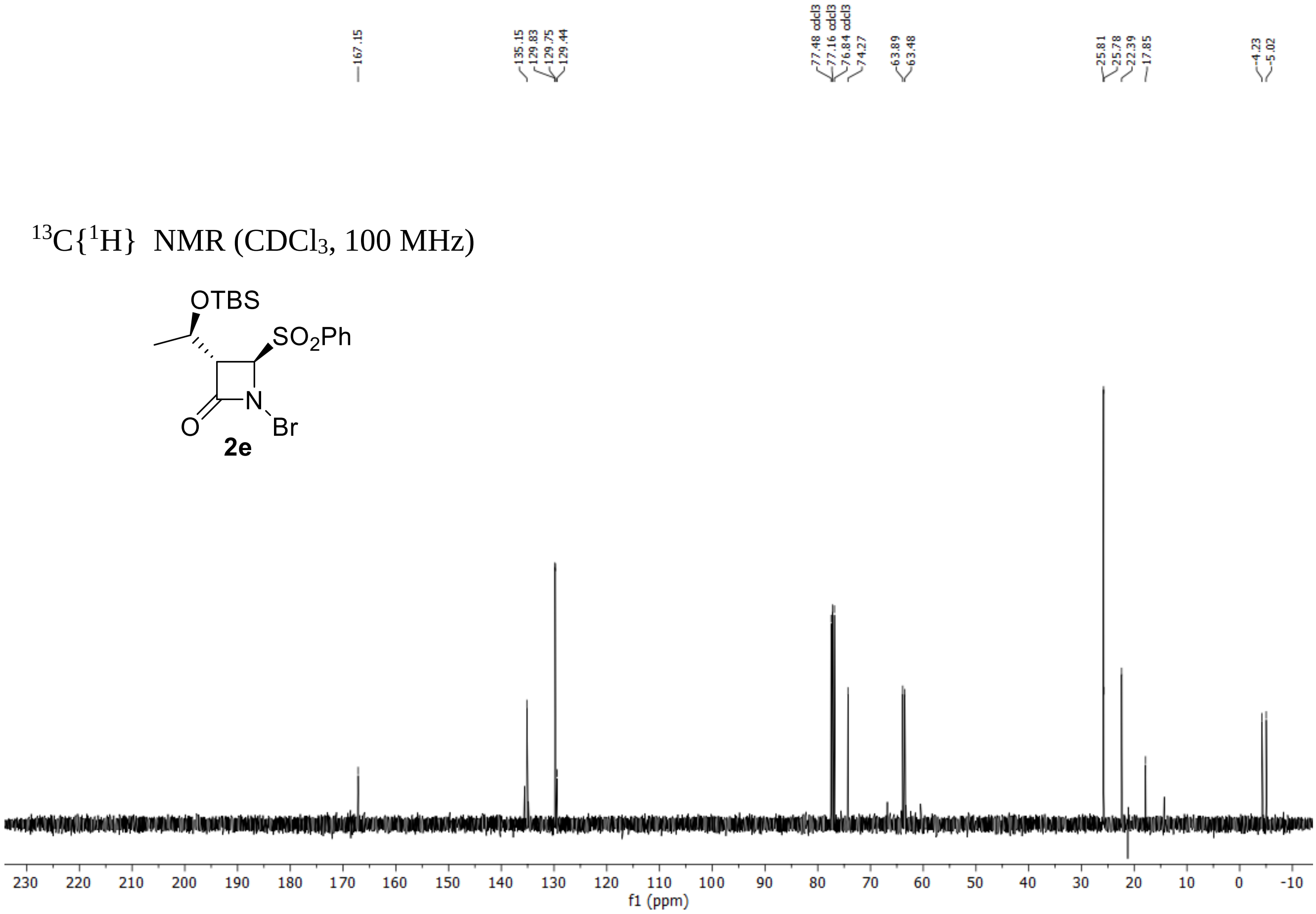

2e

^{1}H NMR analysis of crude mixture

($CDCl_3$, 400 MHz)

2f

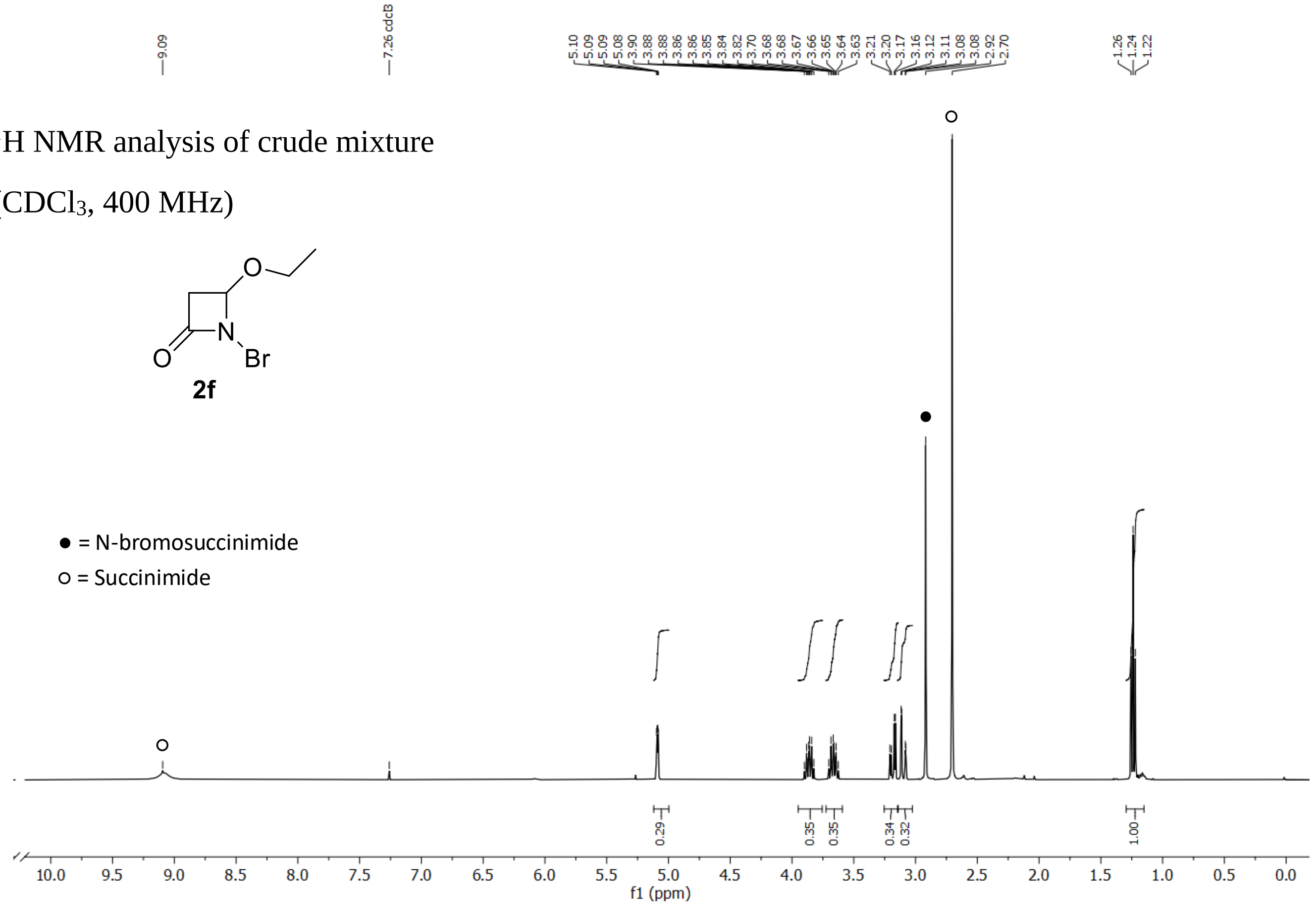

^{13}C{^{1}H}NMR analysis of crude mixture

($CDCl_3$, 100 MHz)

2f

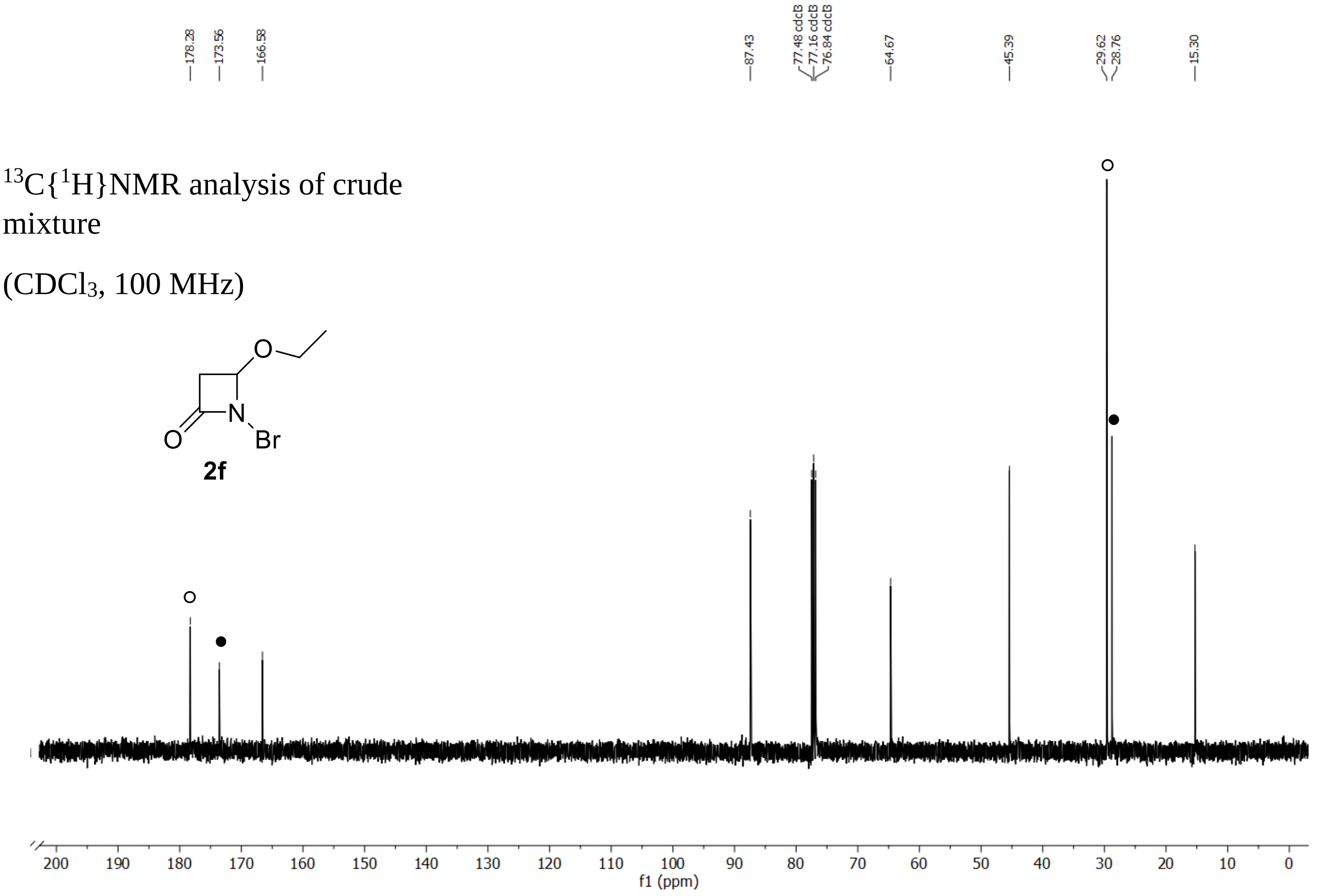

^{1}H NMR ($CDCl_3$, 400 MHz)

2g

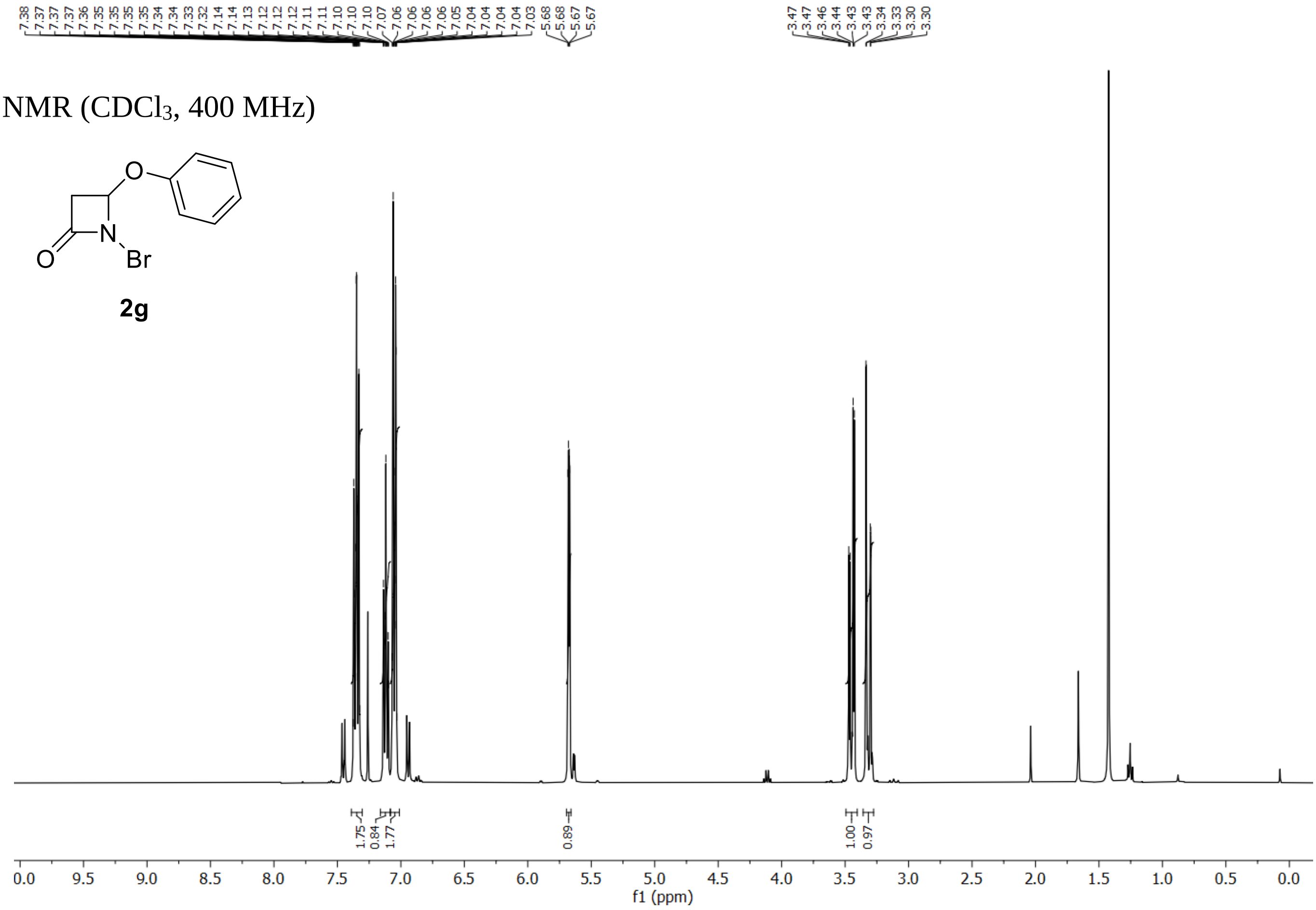

$^{13}C\{^{1}H\}$ NMR ($CDCl_3$, 100 MHz)

2g

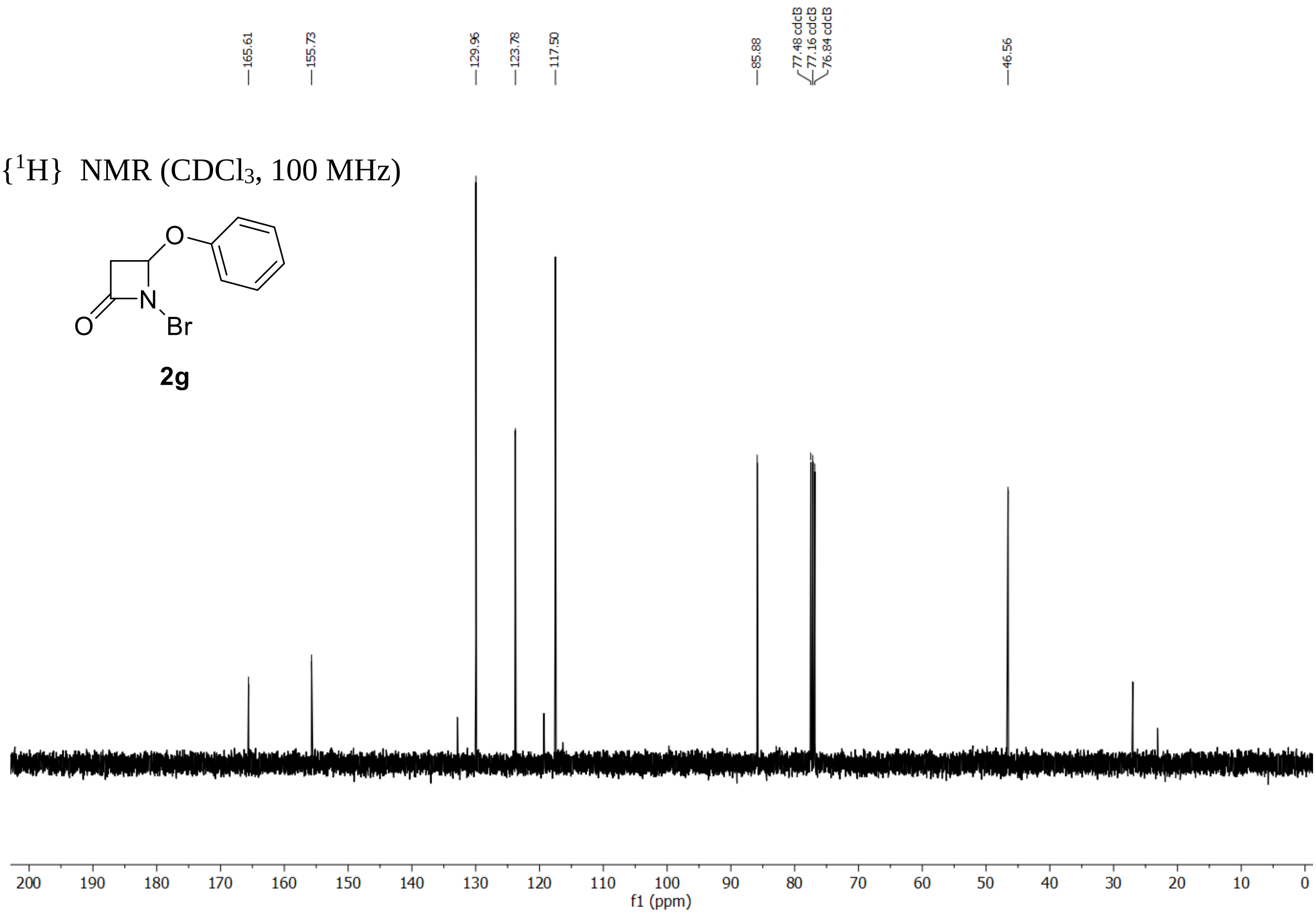

^{1}H NMR ($CDCl_3$, 400 MHz)

2h

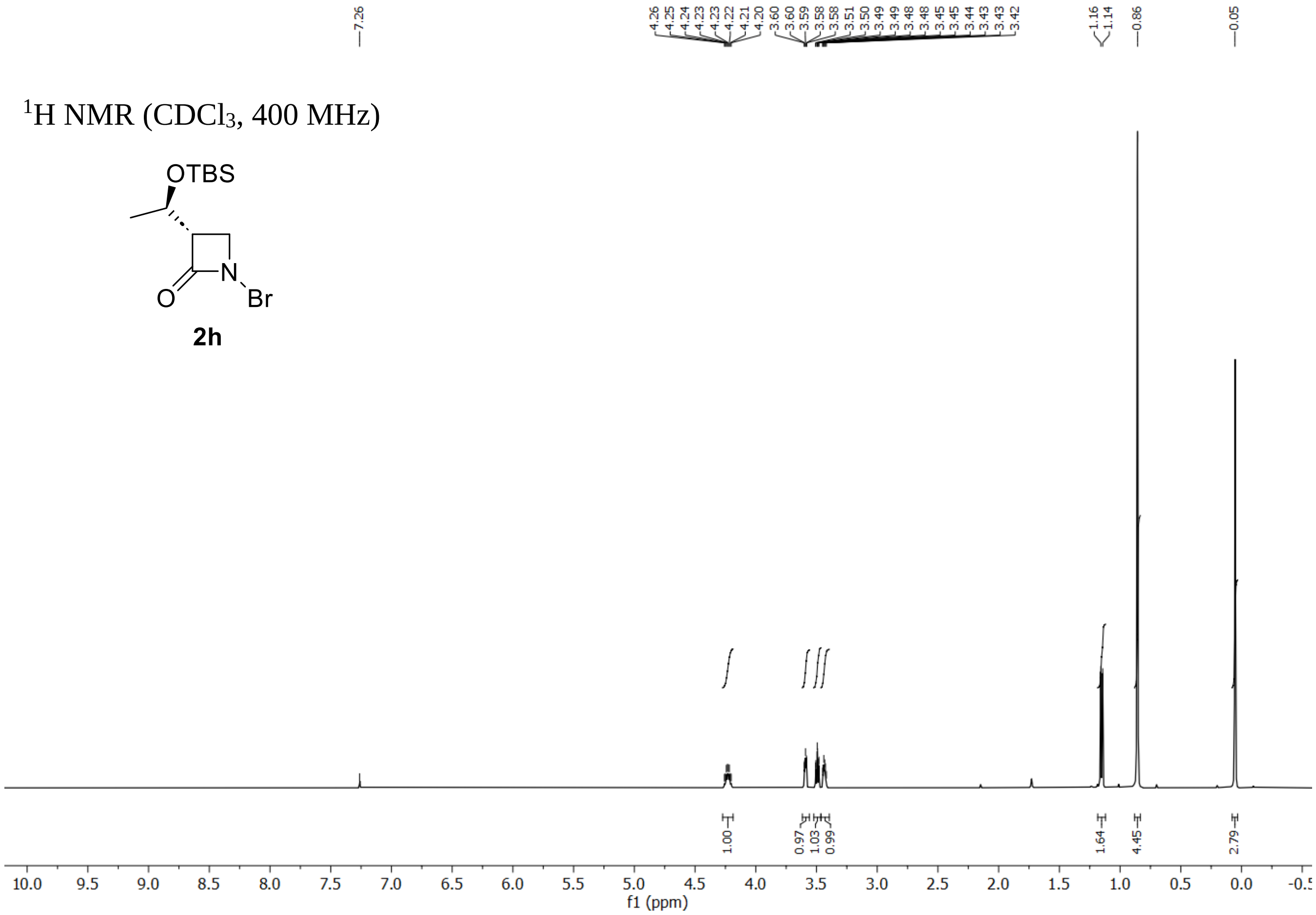

$^{13}C\{^{1}H\}$ NMR ($CDCl_3$, 100 MHz)

2h

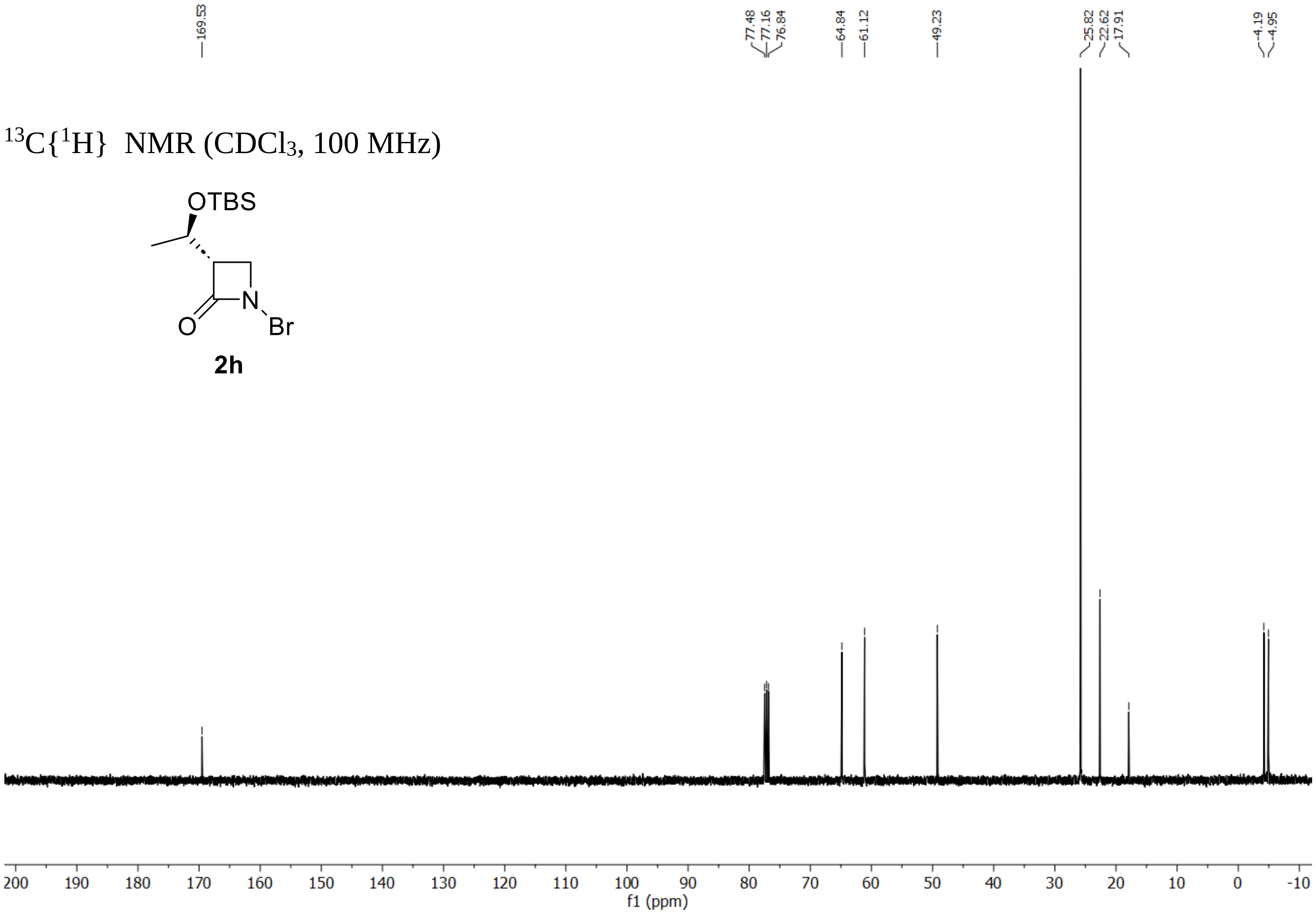

^{1}H NMR ($CDCl_3$, 400 MHz)

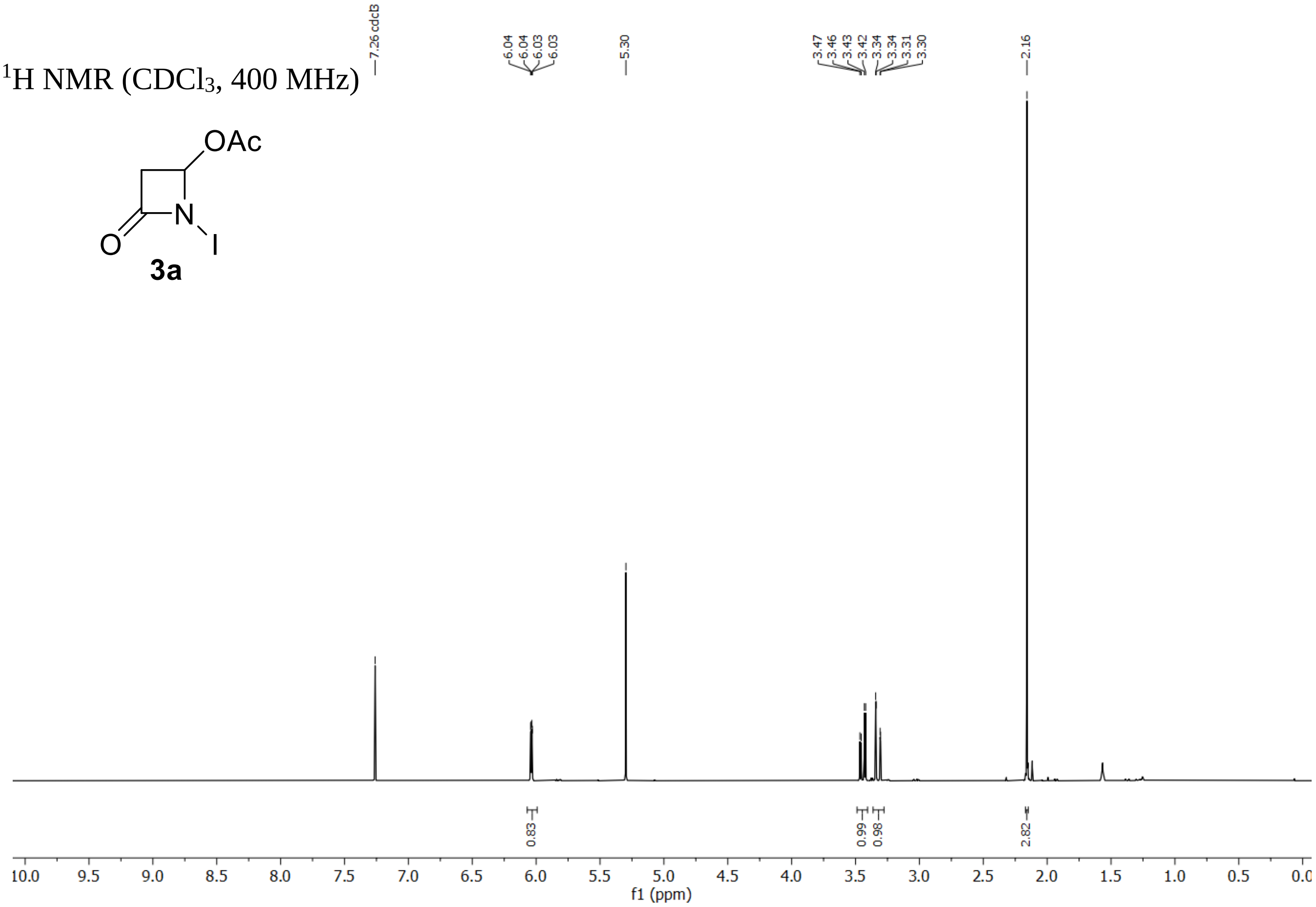

^{13}C{^{1}H} NMR ($CDCl_3$, 100 MHz)

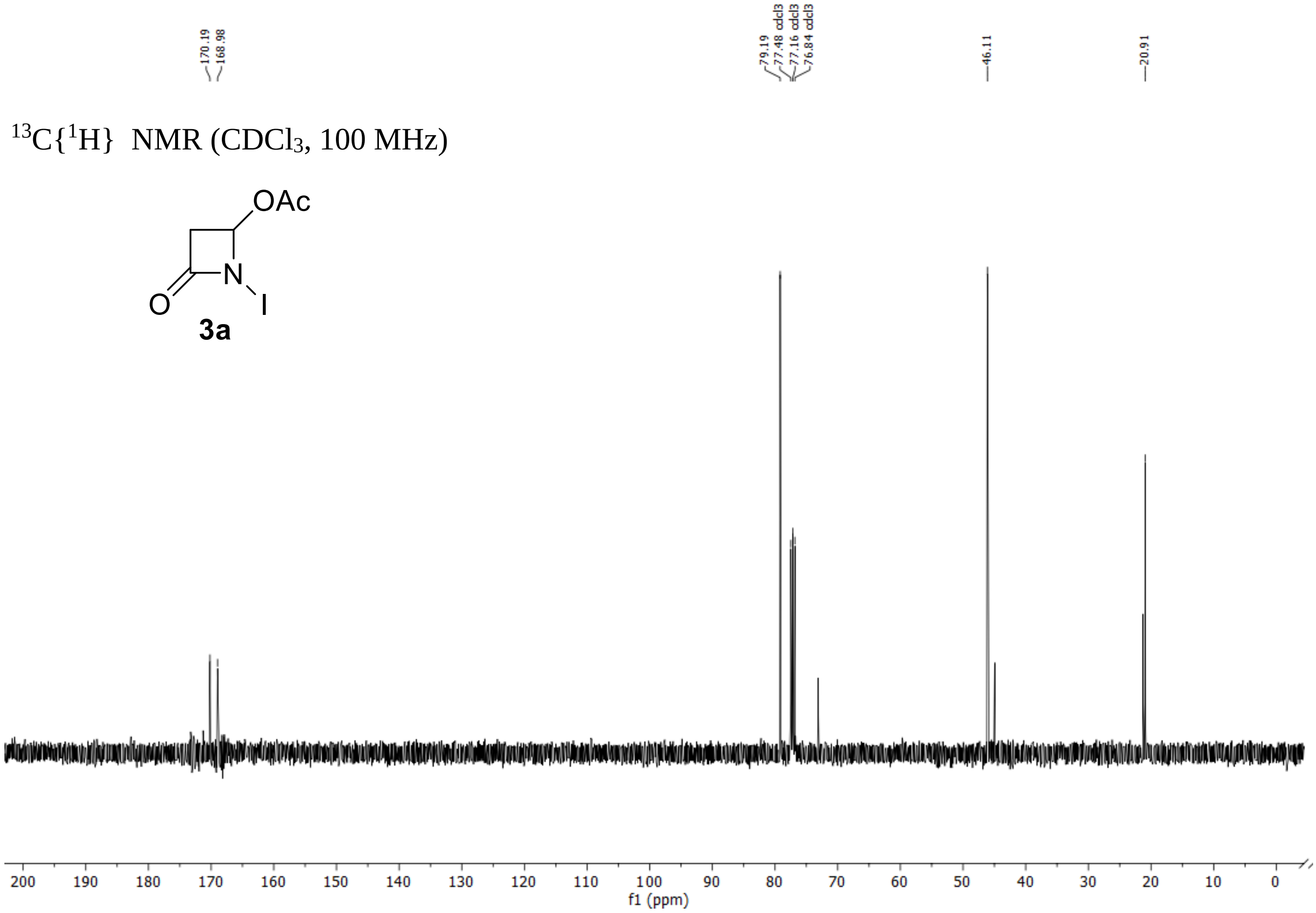

^{1}H NMR ($CDCl_3$, 400 MHz)

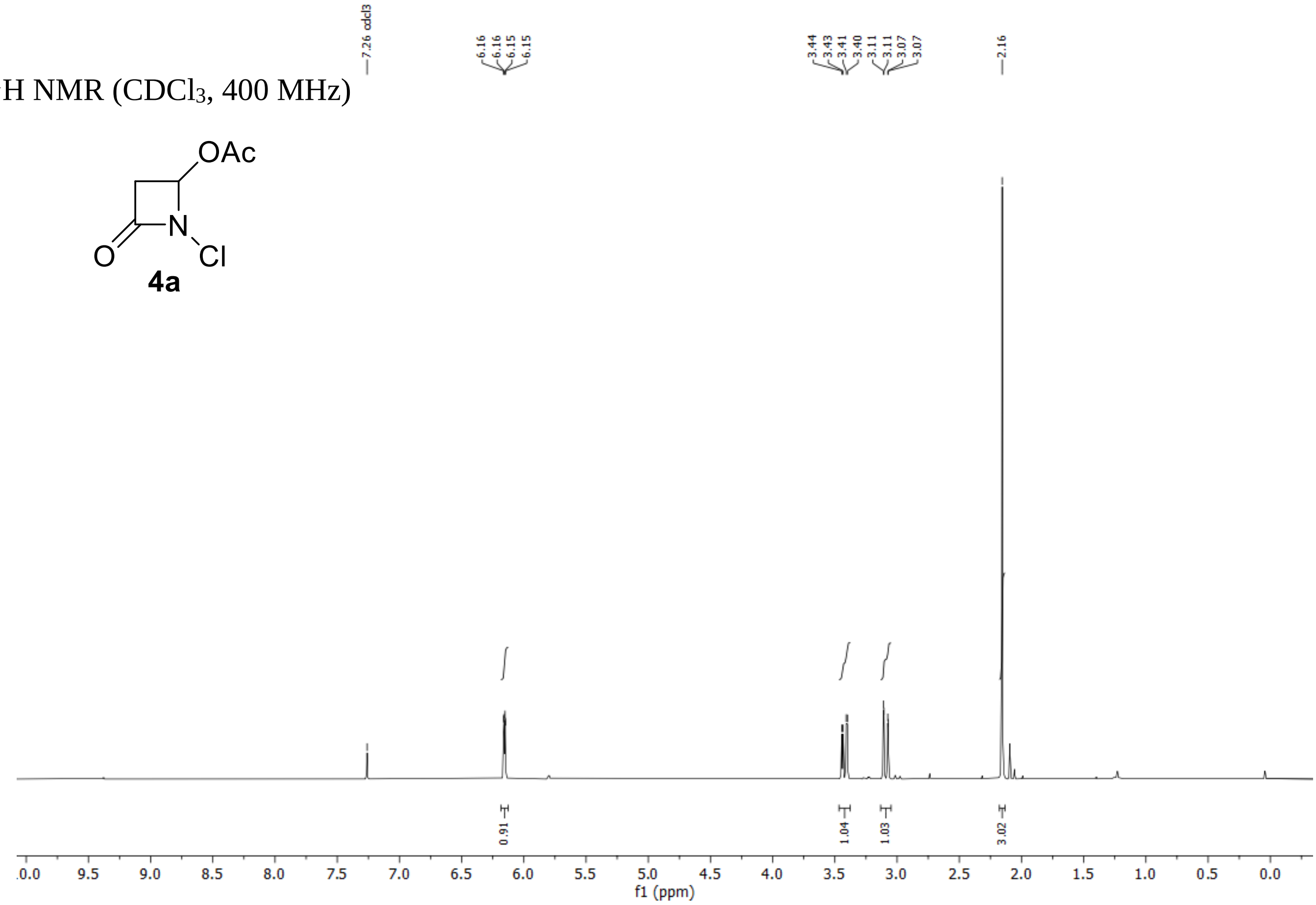

$^{13}C\{^{1}H\}$ NMR ($CDCl_3$, 100 MHz)

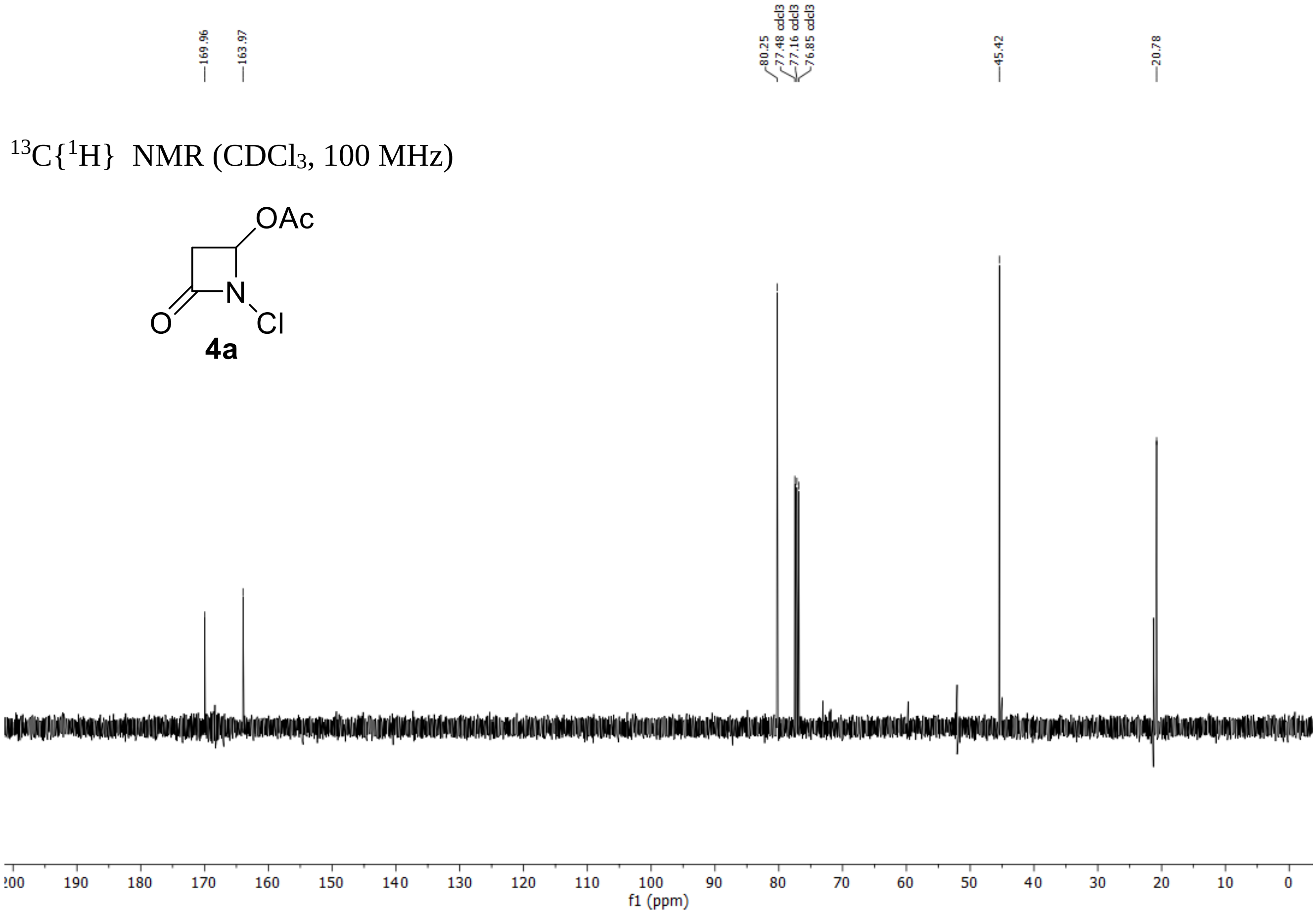

^{1}H NMR ($CDCl_3$, 400 MHz)

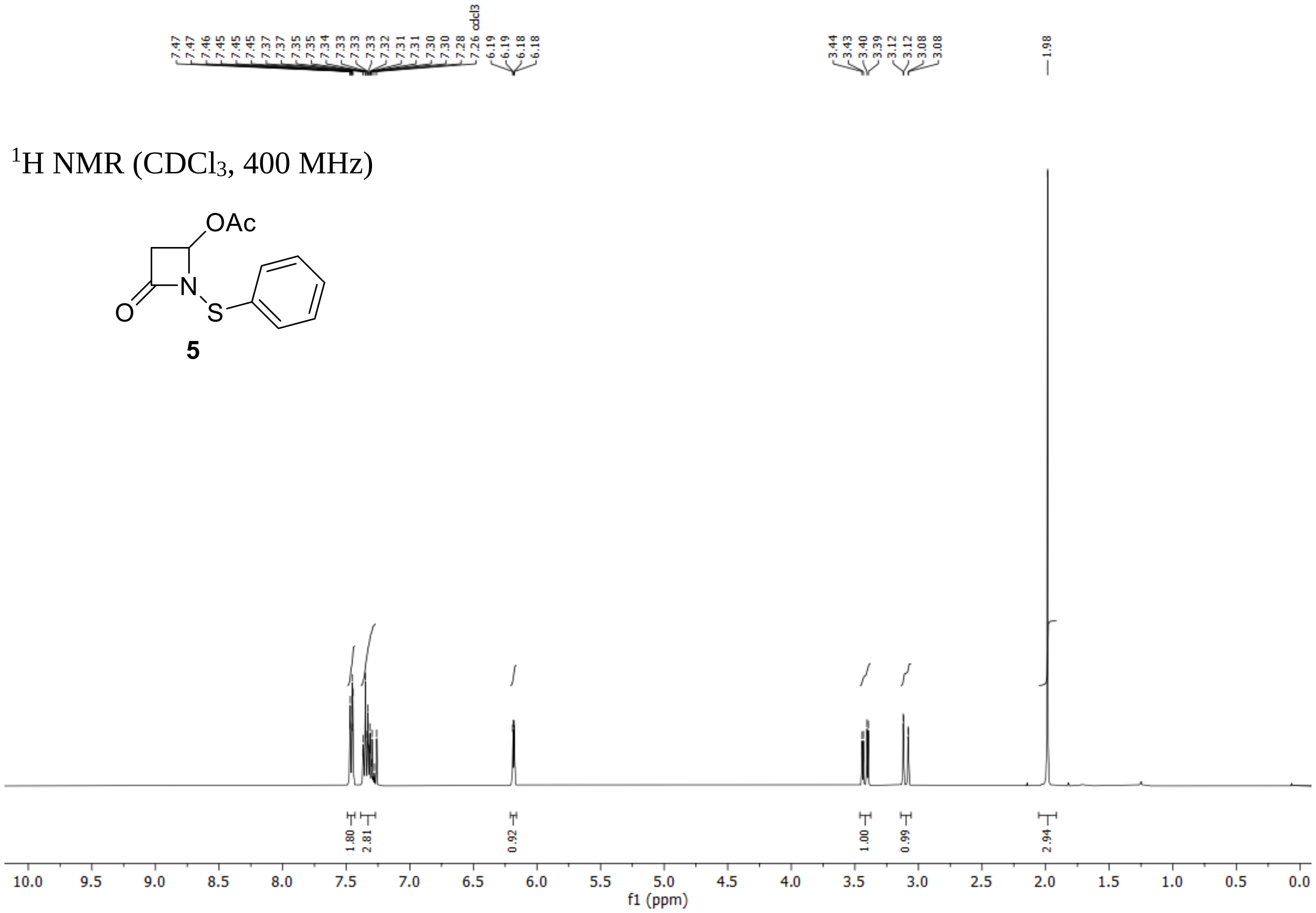

5

^{1}H NMR ($CDCl_3$, 400 MHz)

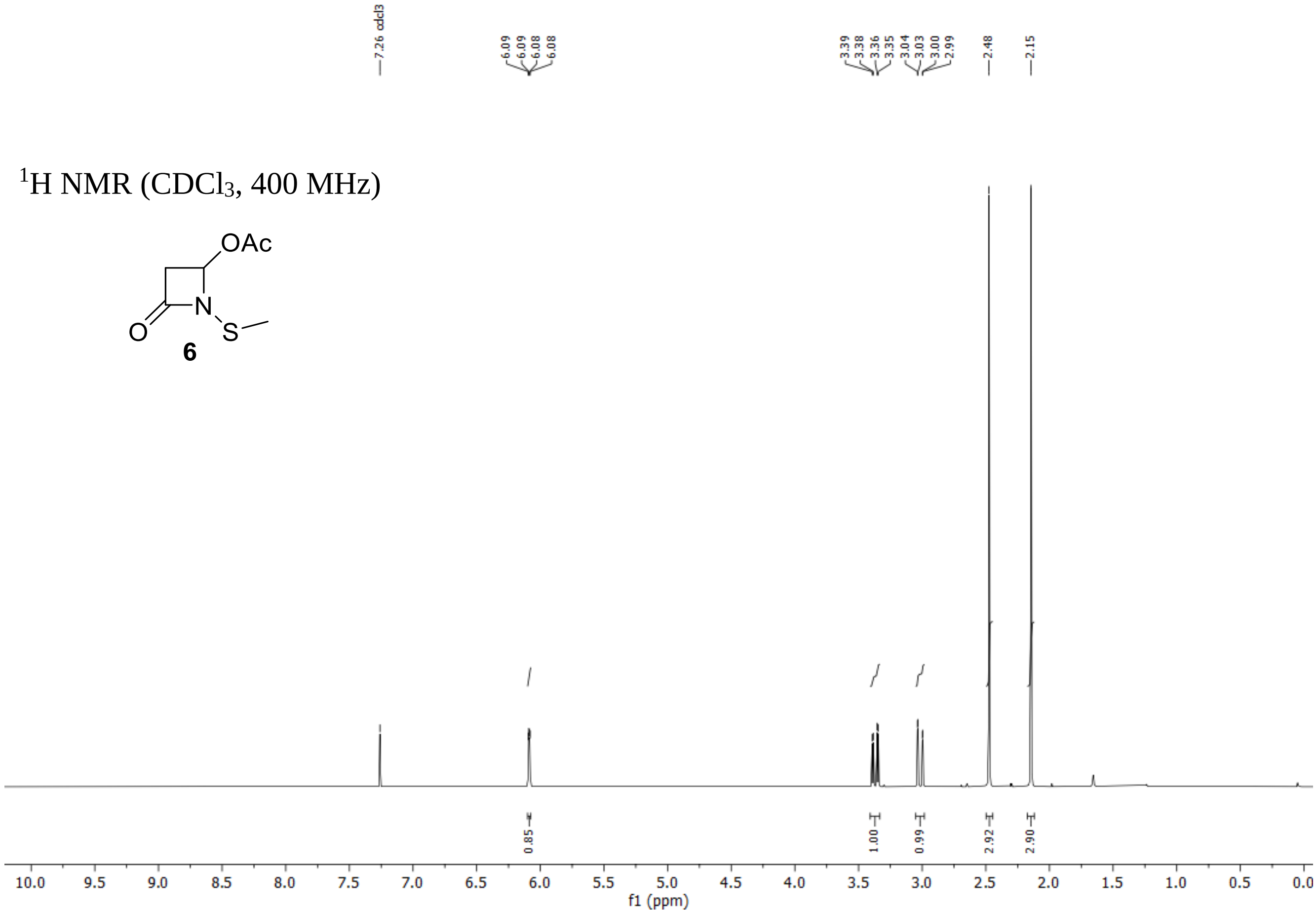

6

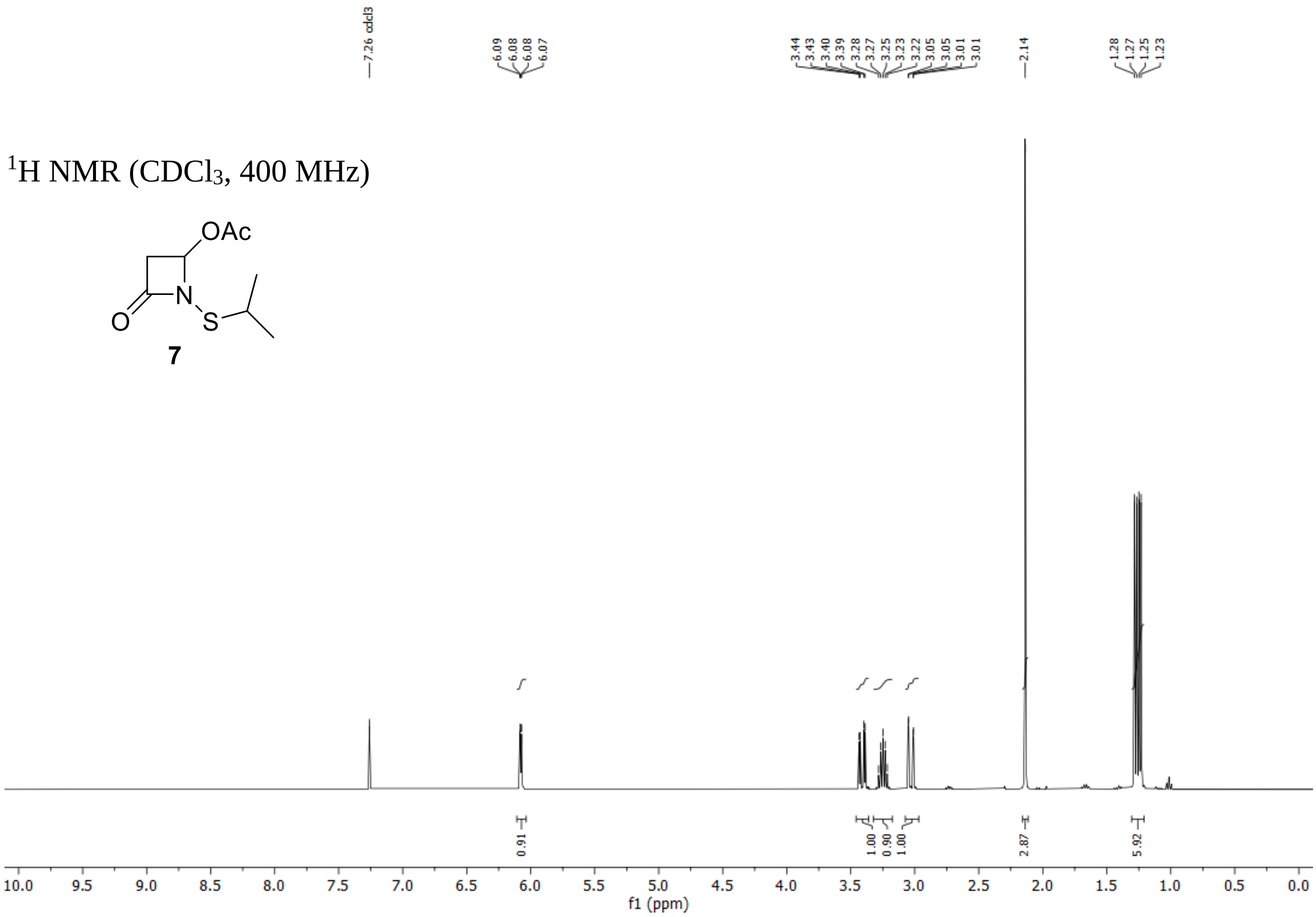
^{1}H NMR ($CDCl_3$, 400 MHz)
OAc
O
N
S
7
7.26 cdcl3
6.09
6.08
6.08
6.07
3.44
3.43
3.40
3.39
3.28
3.27
3.25
3.23
3.22
3.05
3.05
3.01
3.01
2.14
1.28
1.27
1.25
1.23
0.91
1.00
0.90
1.00
2.87
5.92
10.0 9.5 9.0 8.5 8.0 7.5 7.0 6.5 6.0 5.5 5.0 4.5 4.0 3.5 3.0 2.5 2.0 1.5 1.0 0.5 0.0
f1 (ppm)

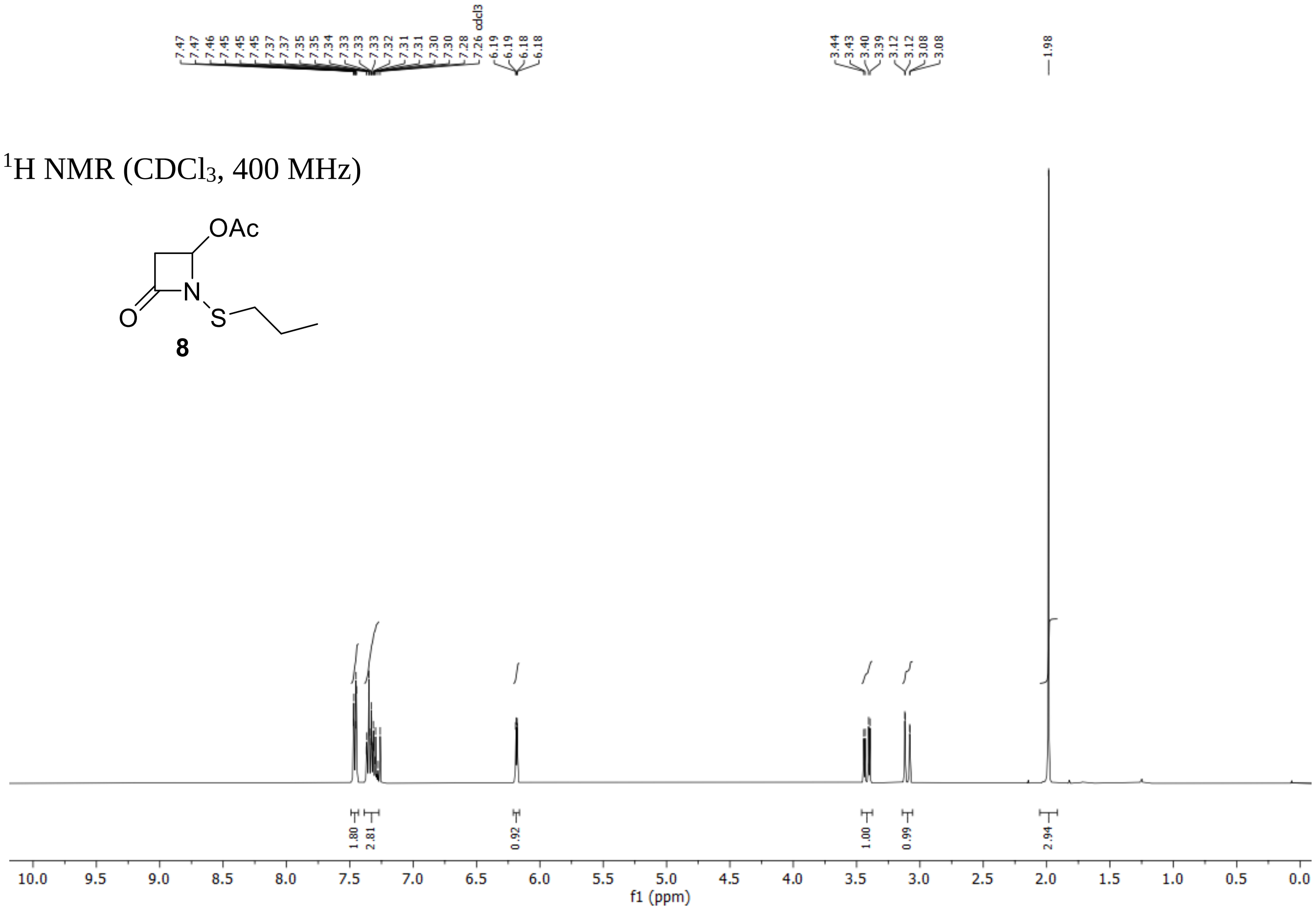
^{1}H NMR ($CDCl_3$, 400 MHz)
OAc
O
N
S
8
7.47
7.47
7.46
7.45
7.45
7.45
7.37
7.37
7.35
7.35
7.34
7.33
7.33
7.33
7.32
7.31
7.31
7.30
7.28
7.26 cdcl3
6.19
6.19
6.18
6.18
3.44
3.43
3.40
3.39
3.12
3.12
3.08
3.08
1.98
1.80
2.81
0.92
1.00
0.99
2.94
10.0 9.5 9.0 8.5 8.0 7.5 7.0 6.5 6.0 5.5 5.0 4.5 4.0 3.5 3.0 2.5 2.0 1.5 1.0 0.5 0.0
f1 (ppm)

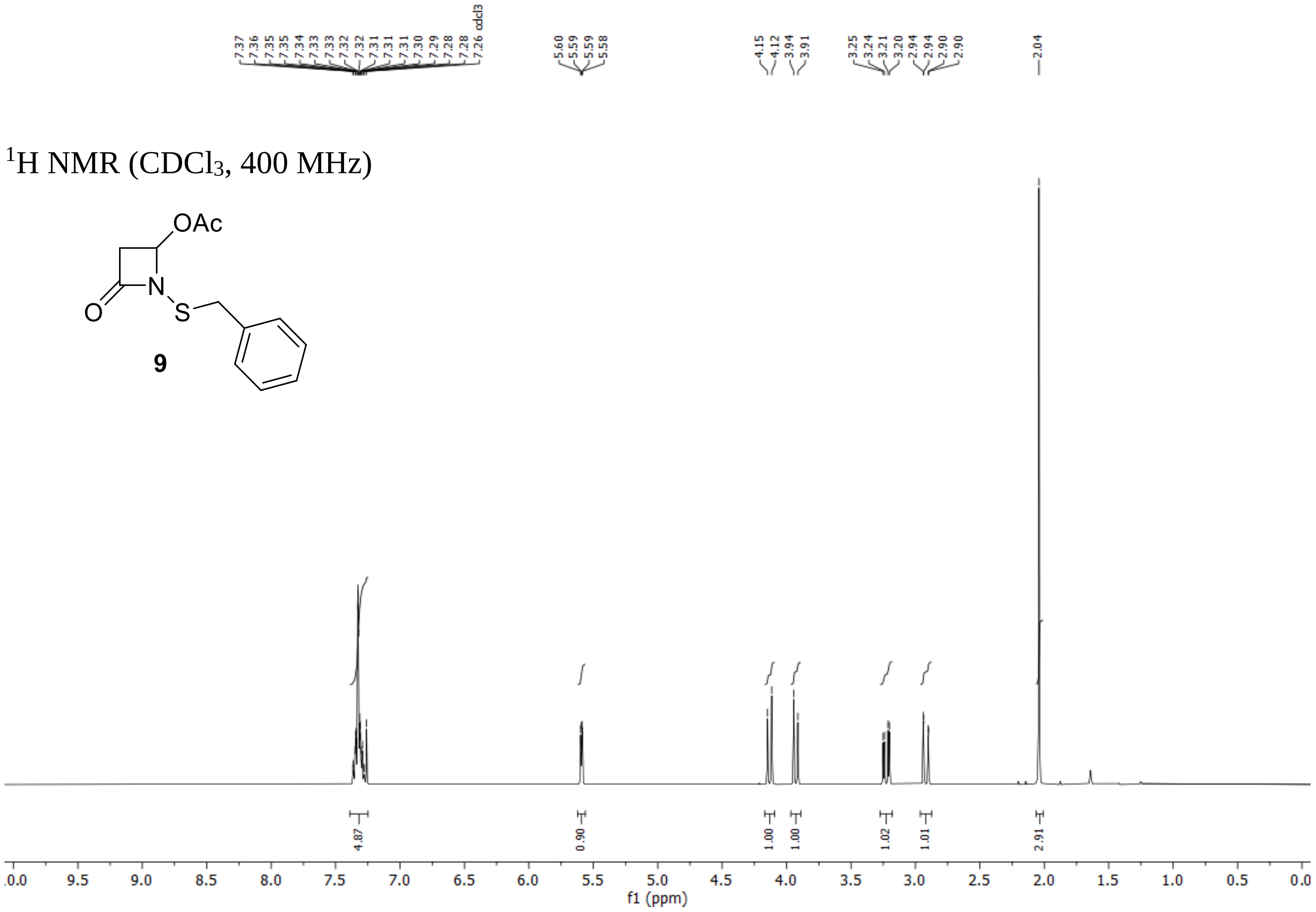

^{1}H NMR (CDCl$_3$, 400 MHz)
OAc
N
S
O
9
f1 (ppm)

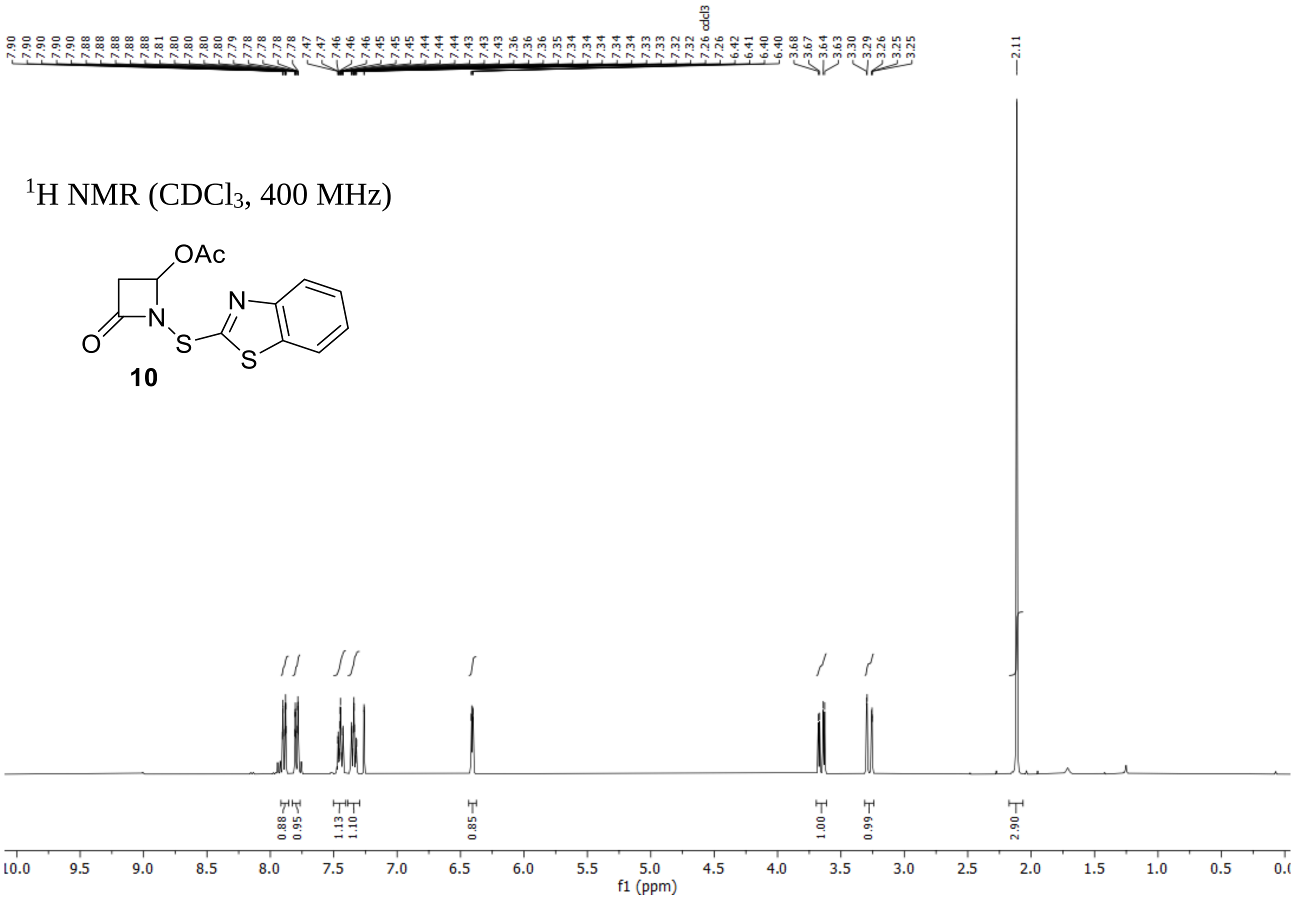

^{1}H NMR (CDCl$_3$, 400 MHz)
OAc
N
S
O
10
f1 (ppm)

^{1}H NMR ($CDCl_3$, 400 MHz)

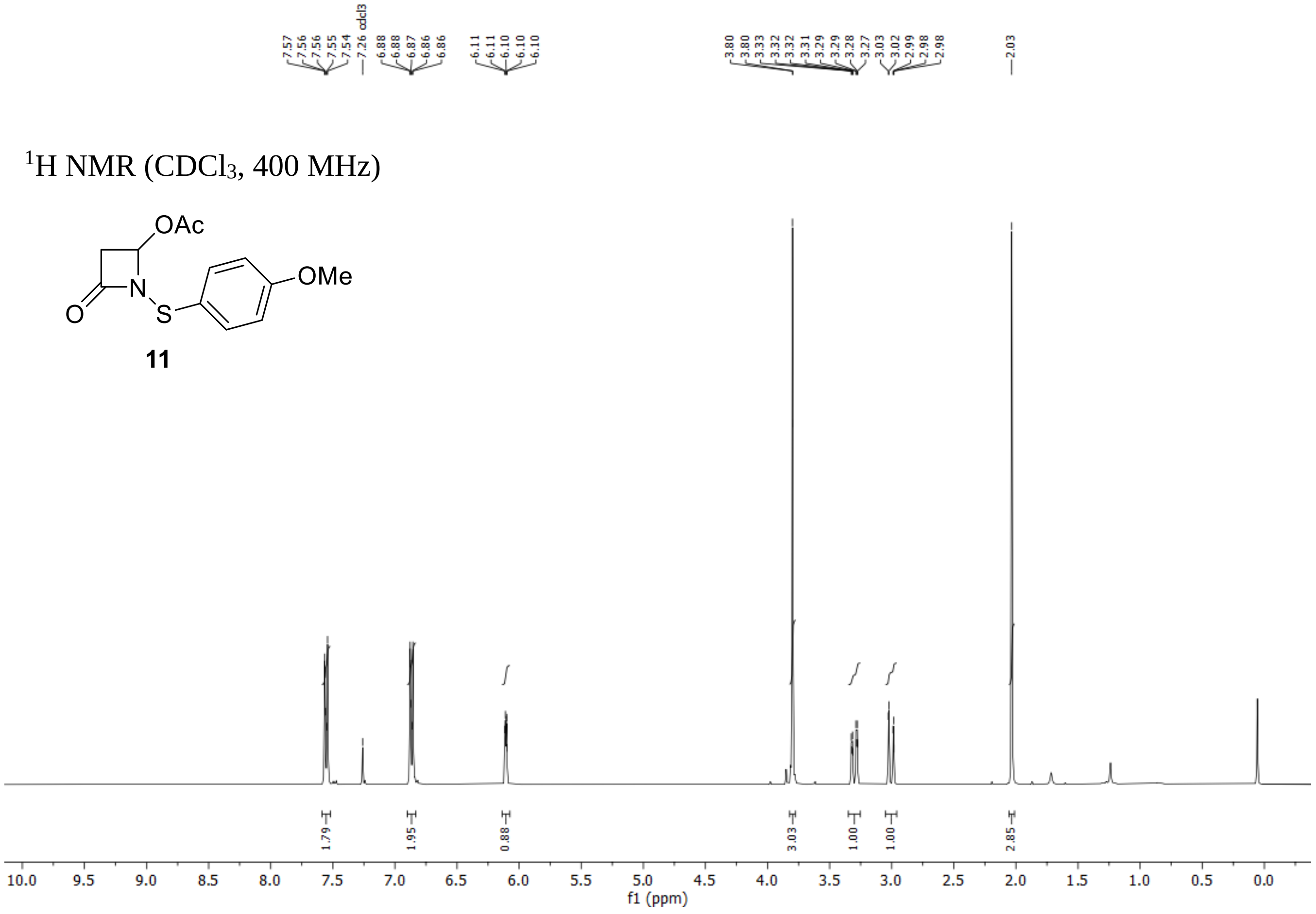

^{1}H NMR ($CDCl_3$, 400 MHz)

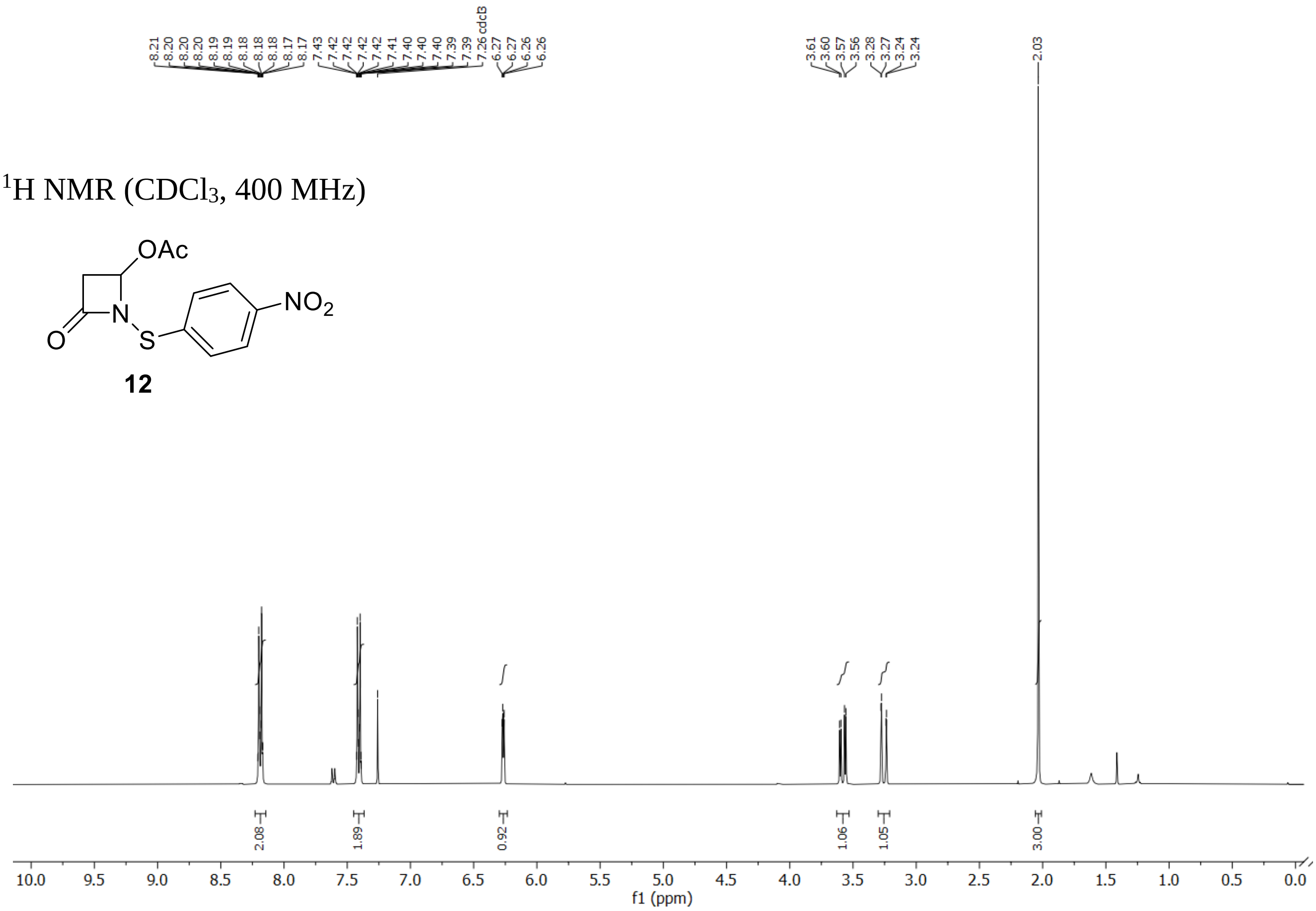

12

^{13}C{^{1}H} NMR ($CDCl_3$, 100 MHz)

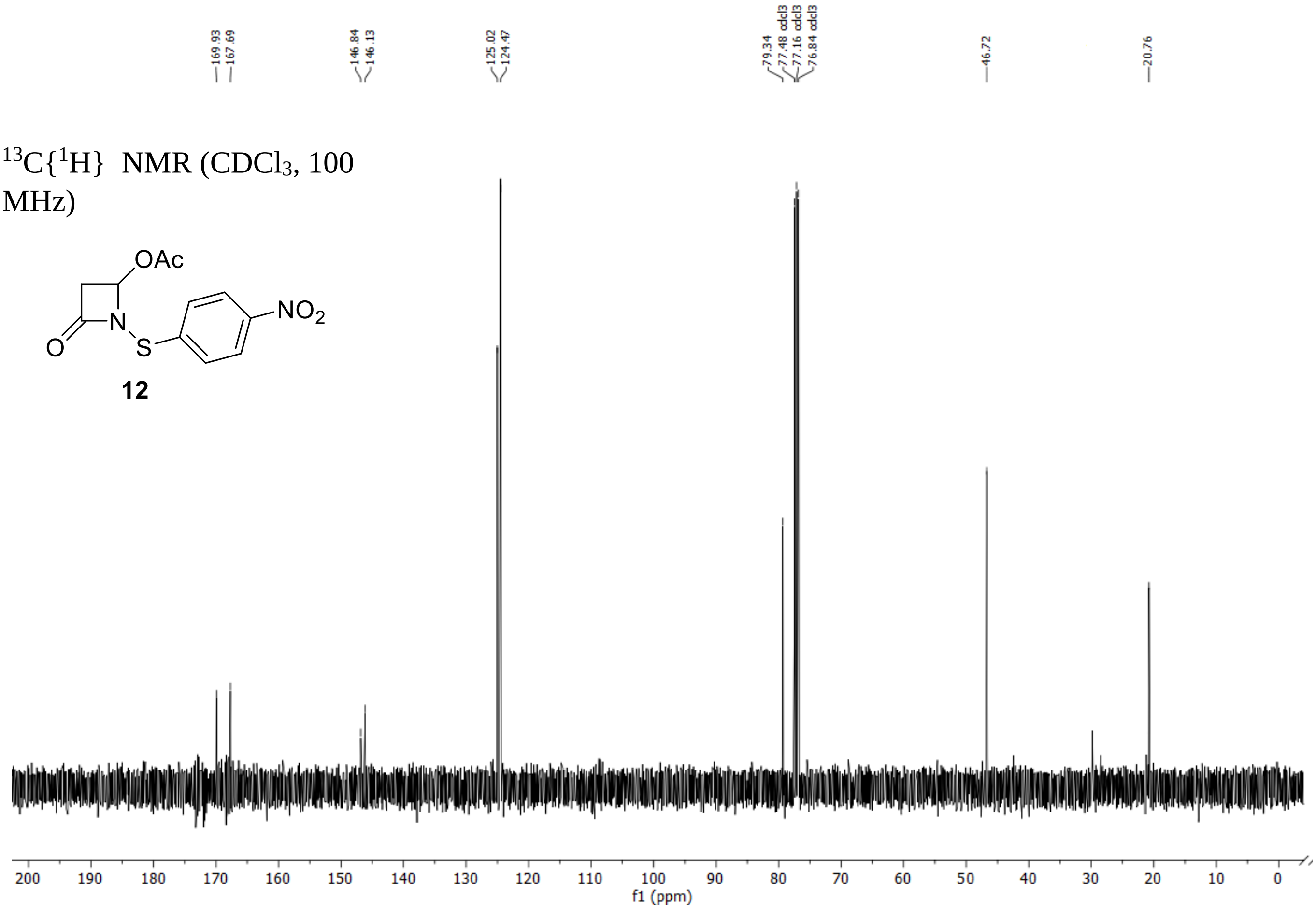

12

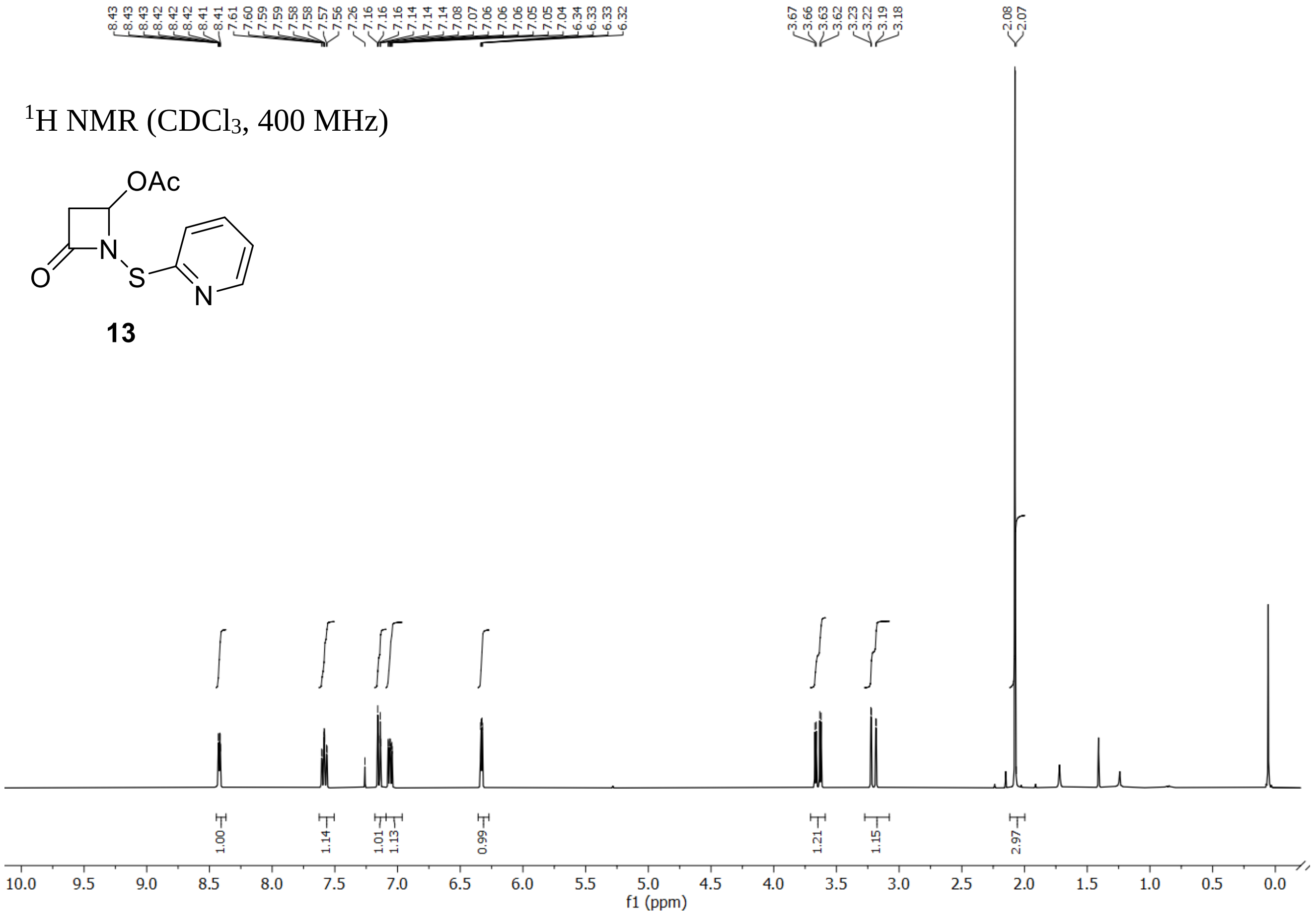

^{1}H NMR ($CDCl_3$, 400 MHz)

13

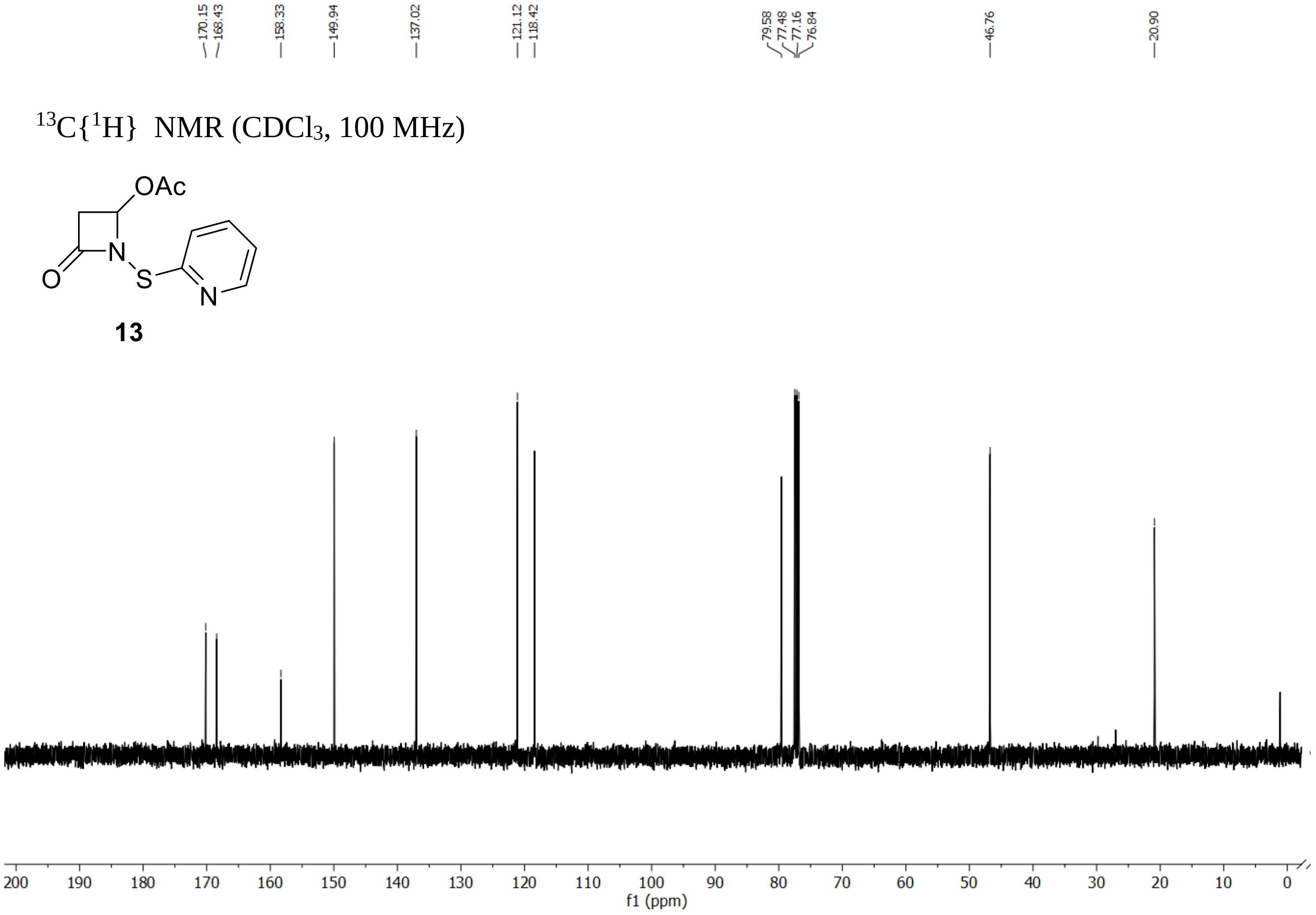

$^{13}C\{^{1}H\}$ NMR ($CDCl_3$, 100 MHz)

13

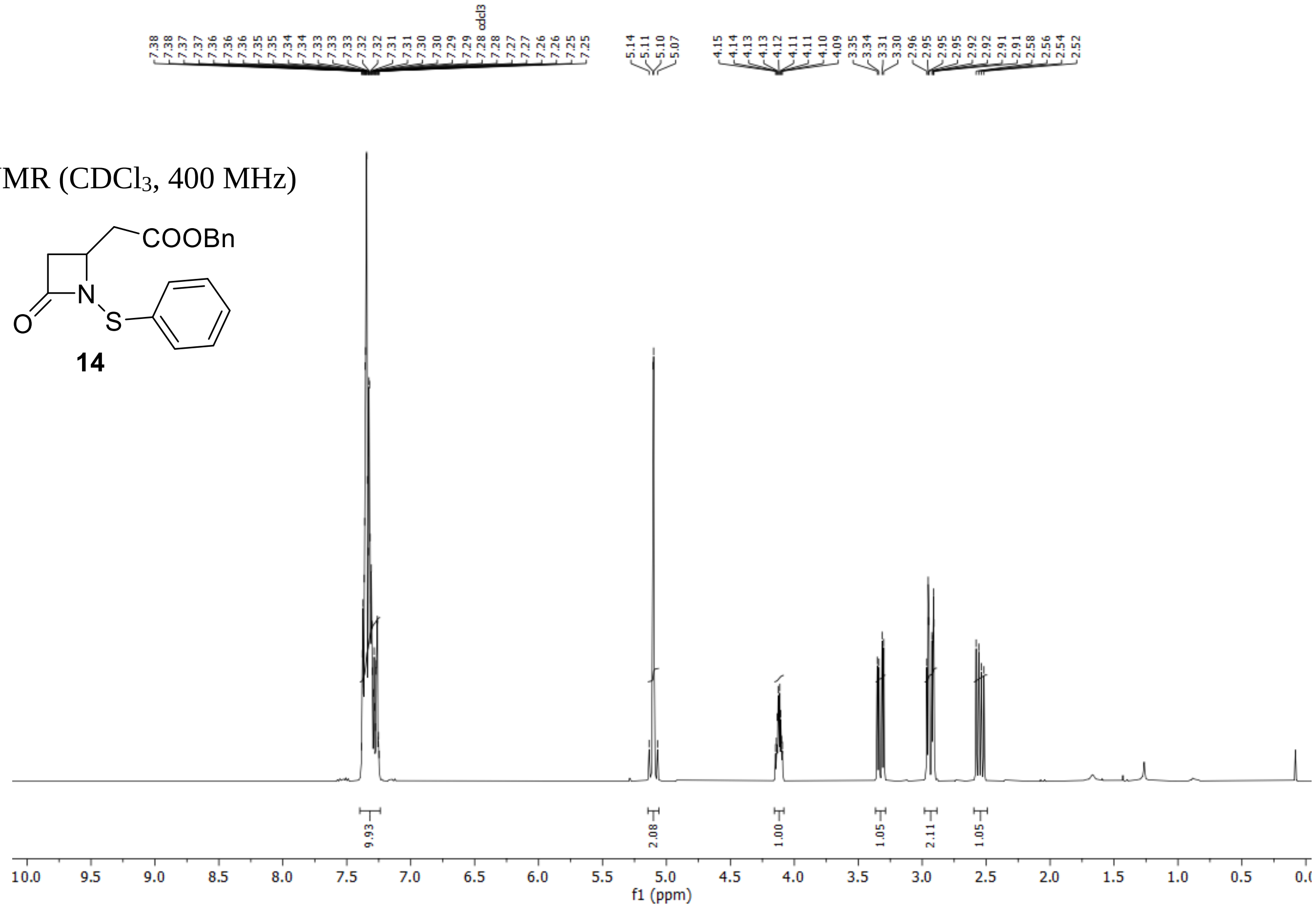

^{1}H NMR ($CDCl_3$, 400 MHz)

14

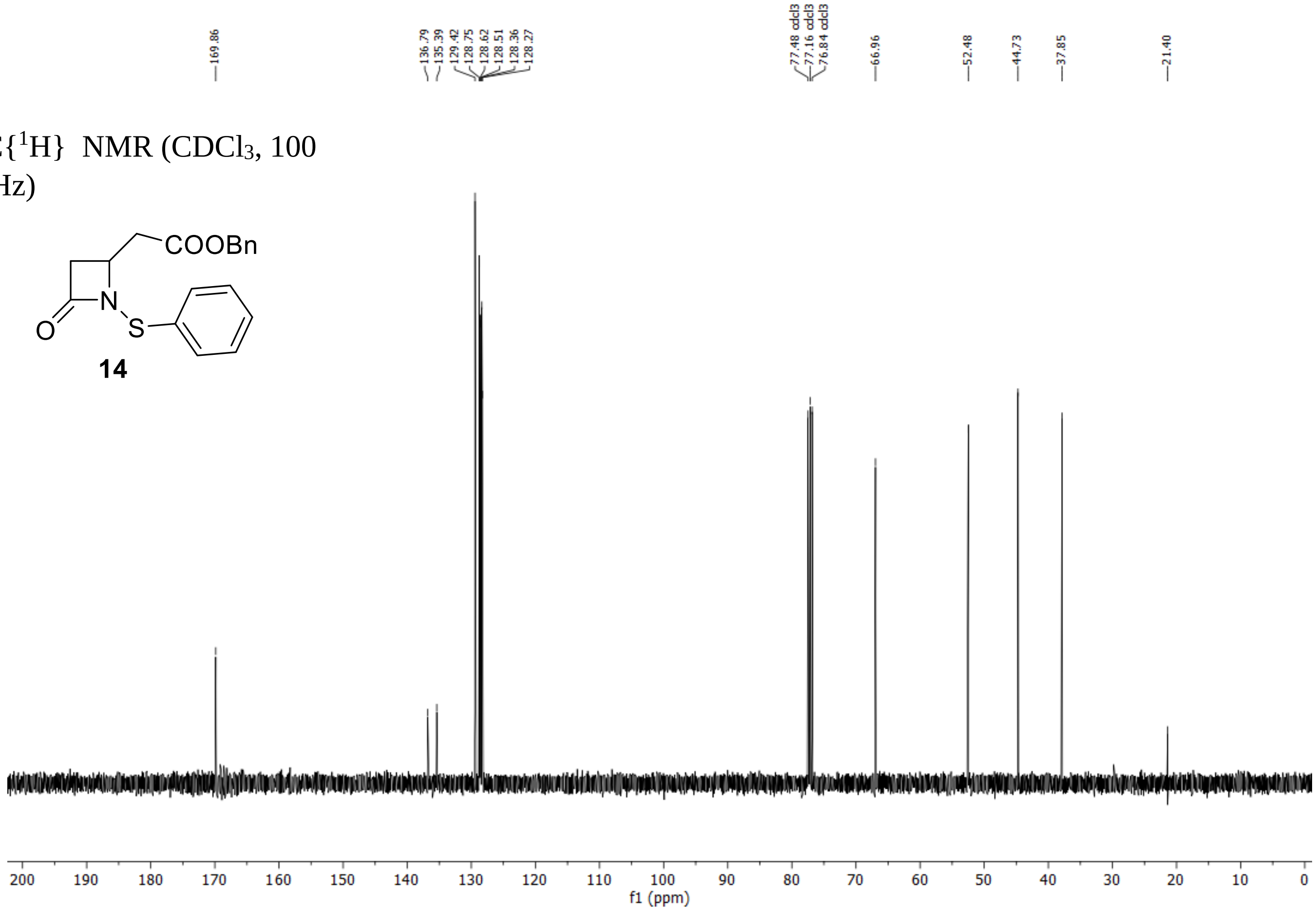

$^{13}C\{^{1}H\}$ NMR ($CDCl_3$, 100 MHz)

14

^{1}H NMR ($CDCl_3$, 400 MHz)

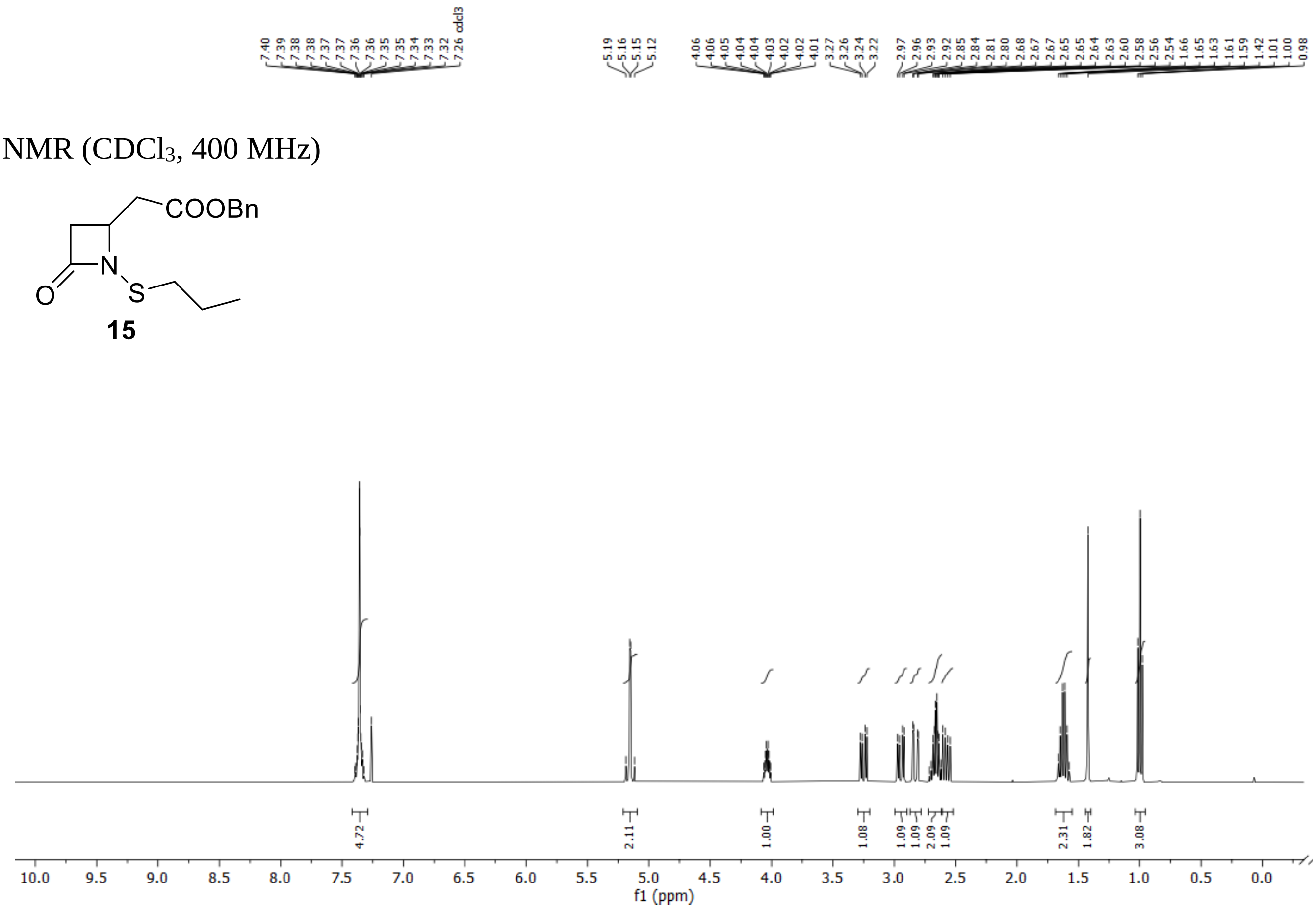

15

$^{13}C\{^{1}H\}$ NMR ($CDCl_3$, 100 MHz)

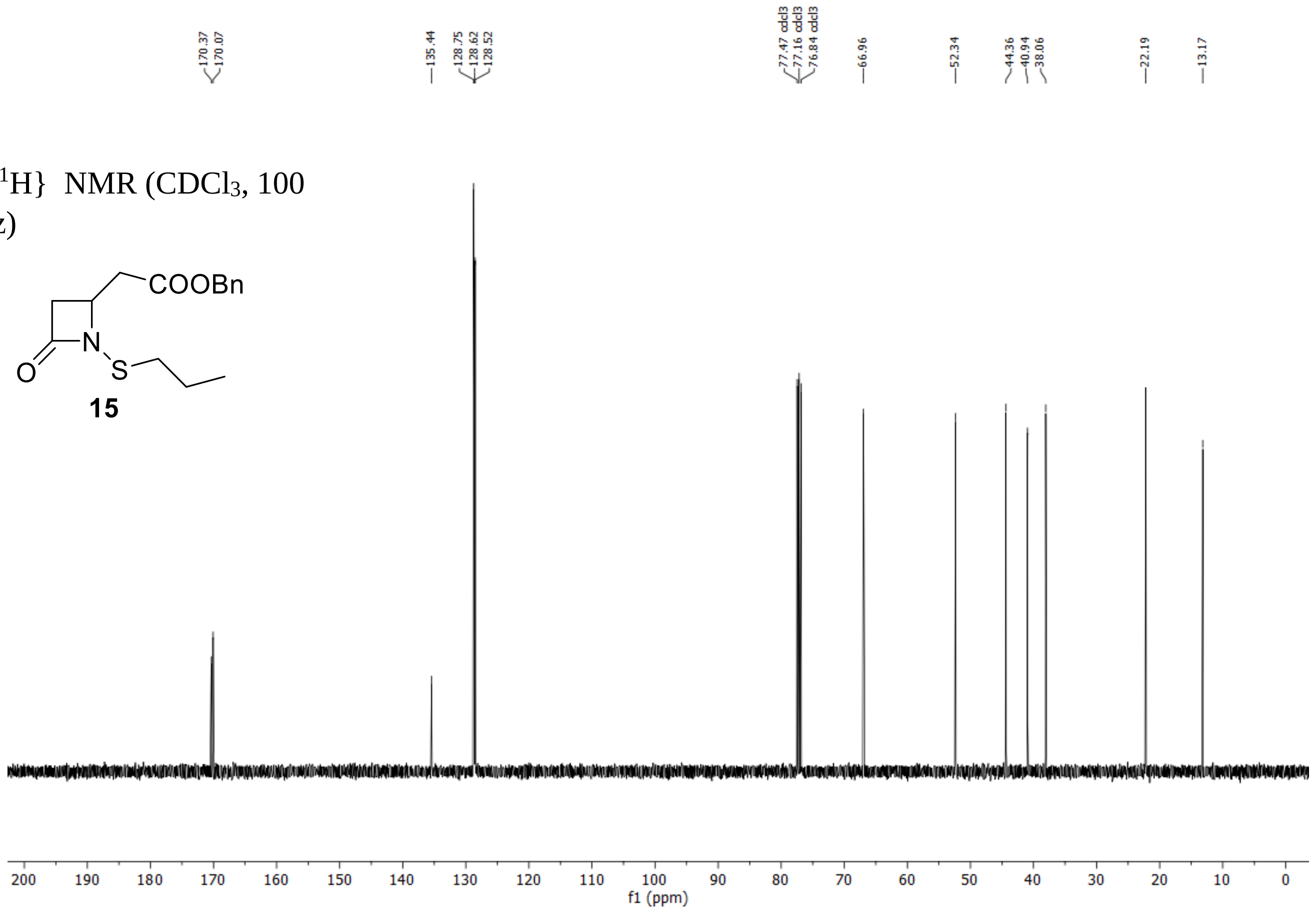

15

^{1}H NMR ($CDCl_3$, 400 MHz)

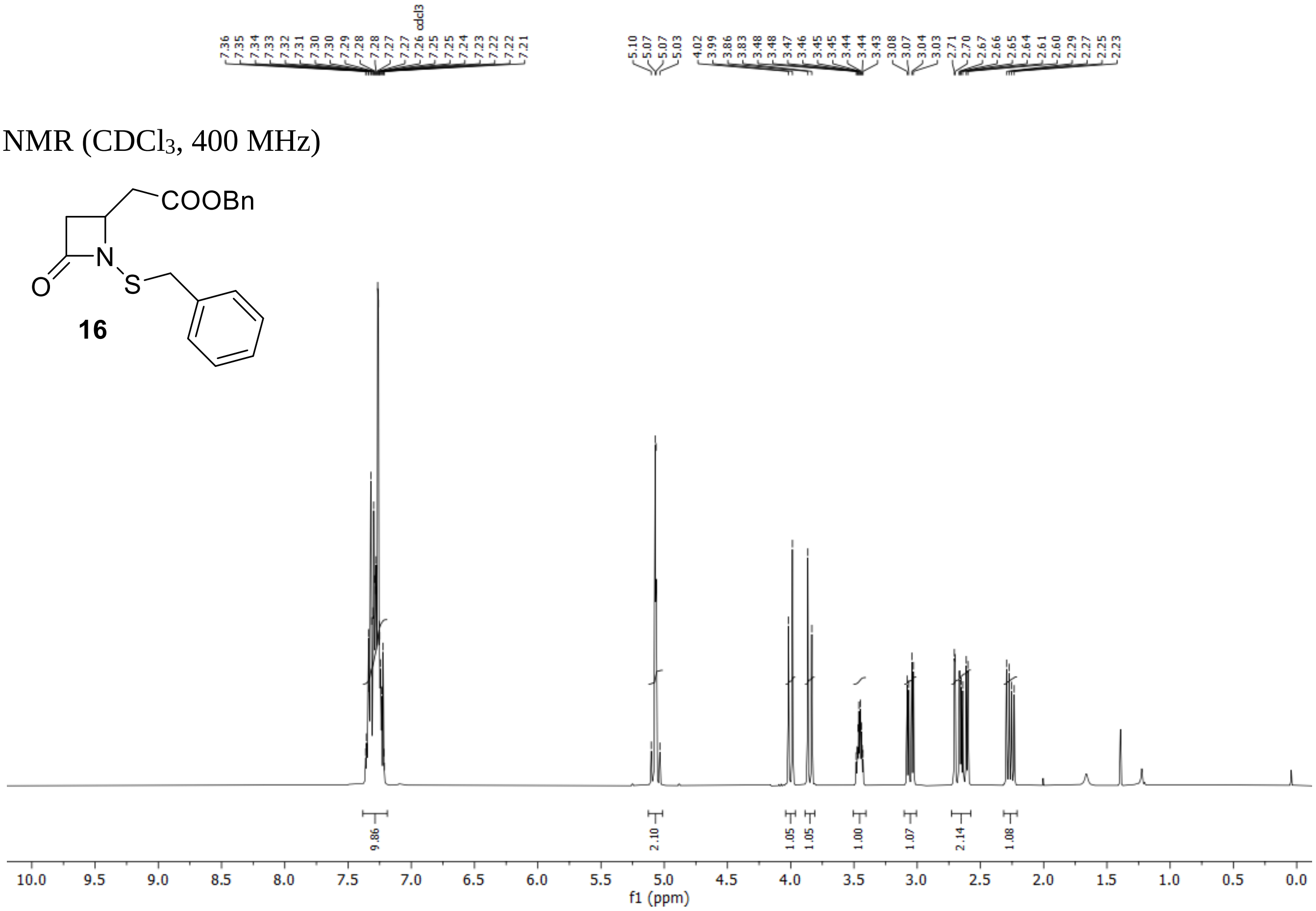

16

$^{13}C\{^{1}H\}$ NMR ($CDCl_3$, 100 MHz)

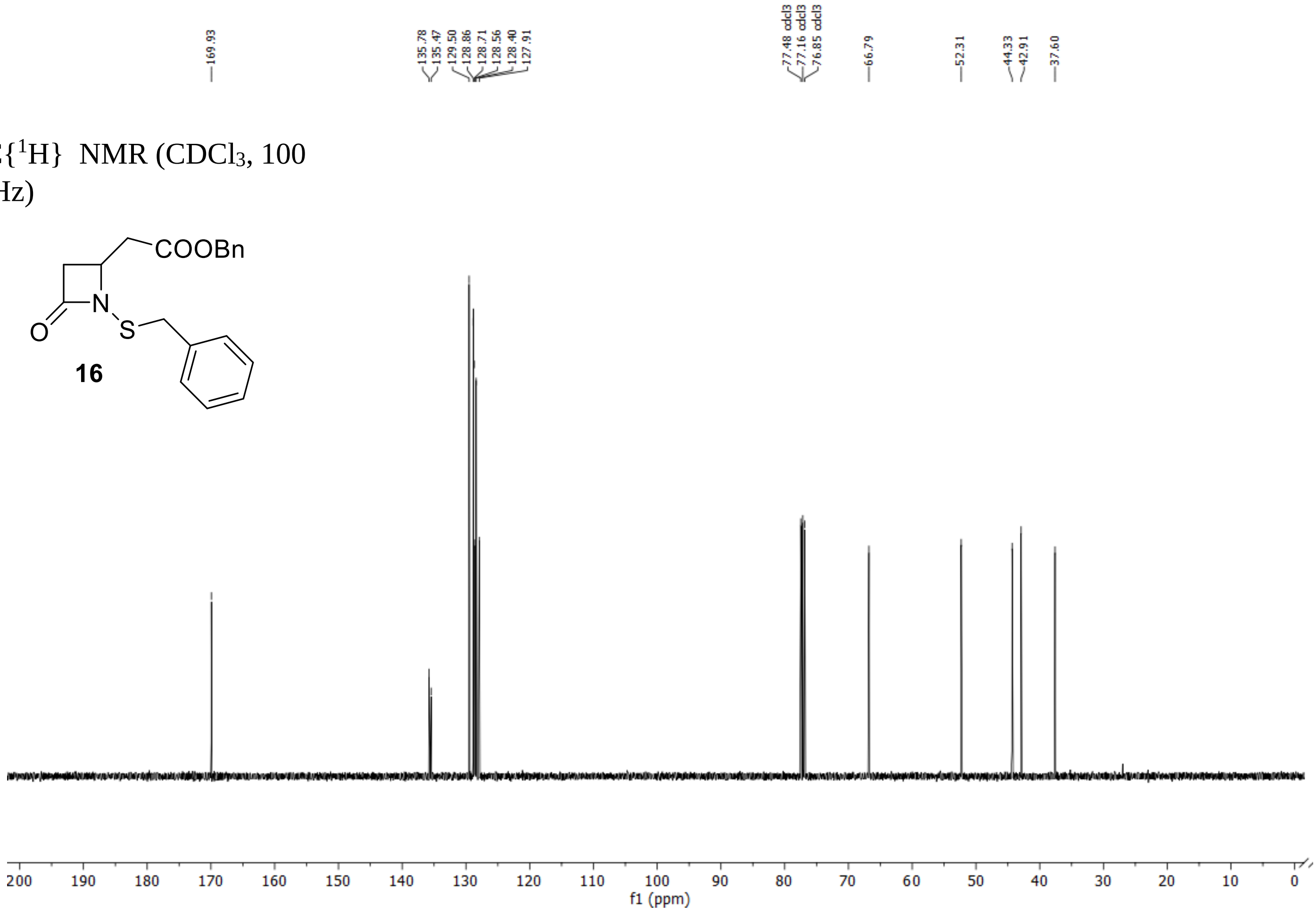

16

^{1}H NMR ($CDCl_3$, 400 MHz)

17

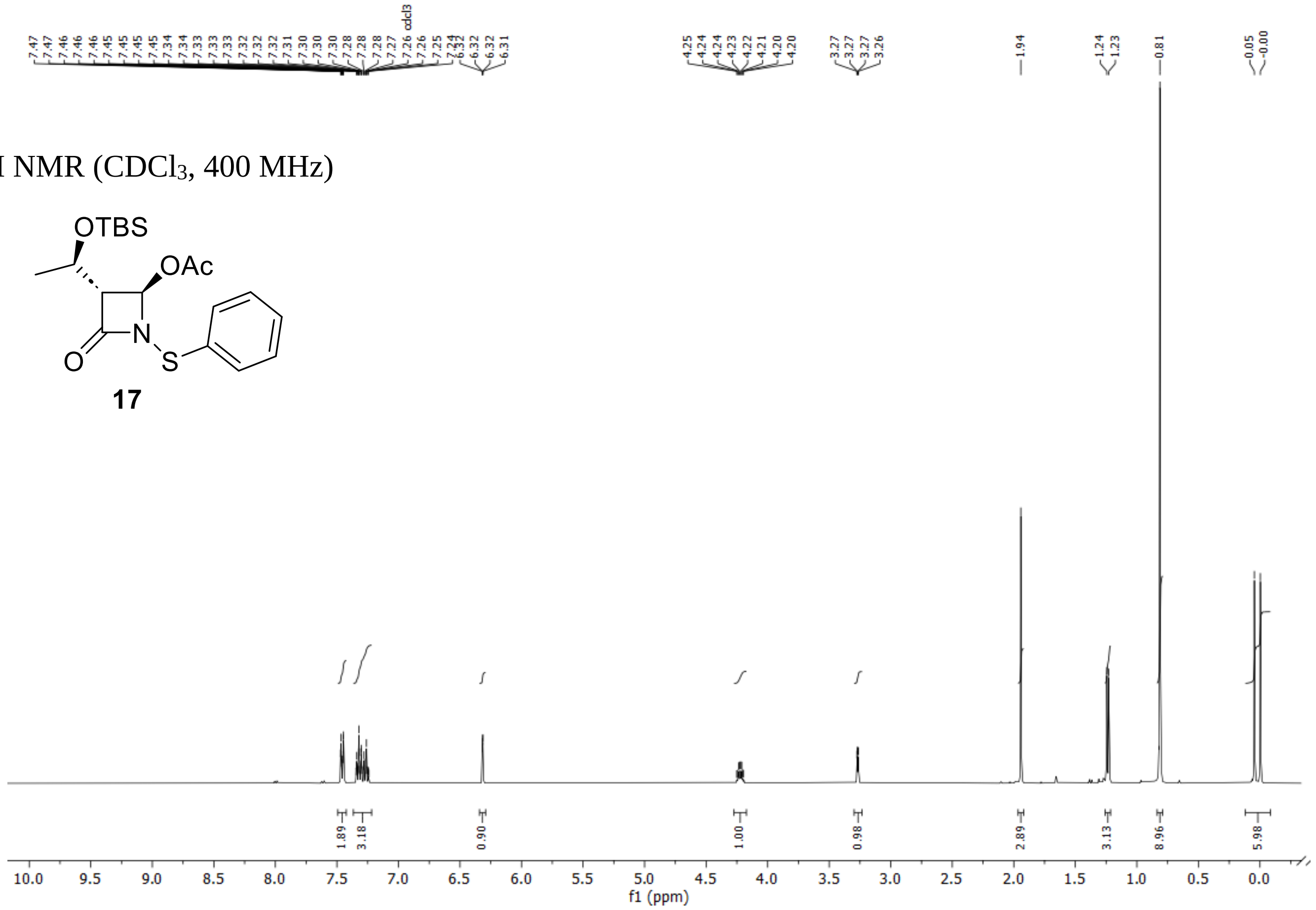

^{1}H NMR ($CDCl_3$, 400 MHz)

18

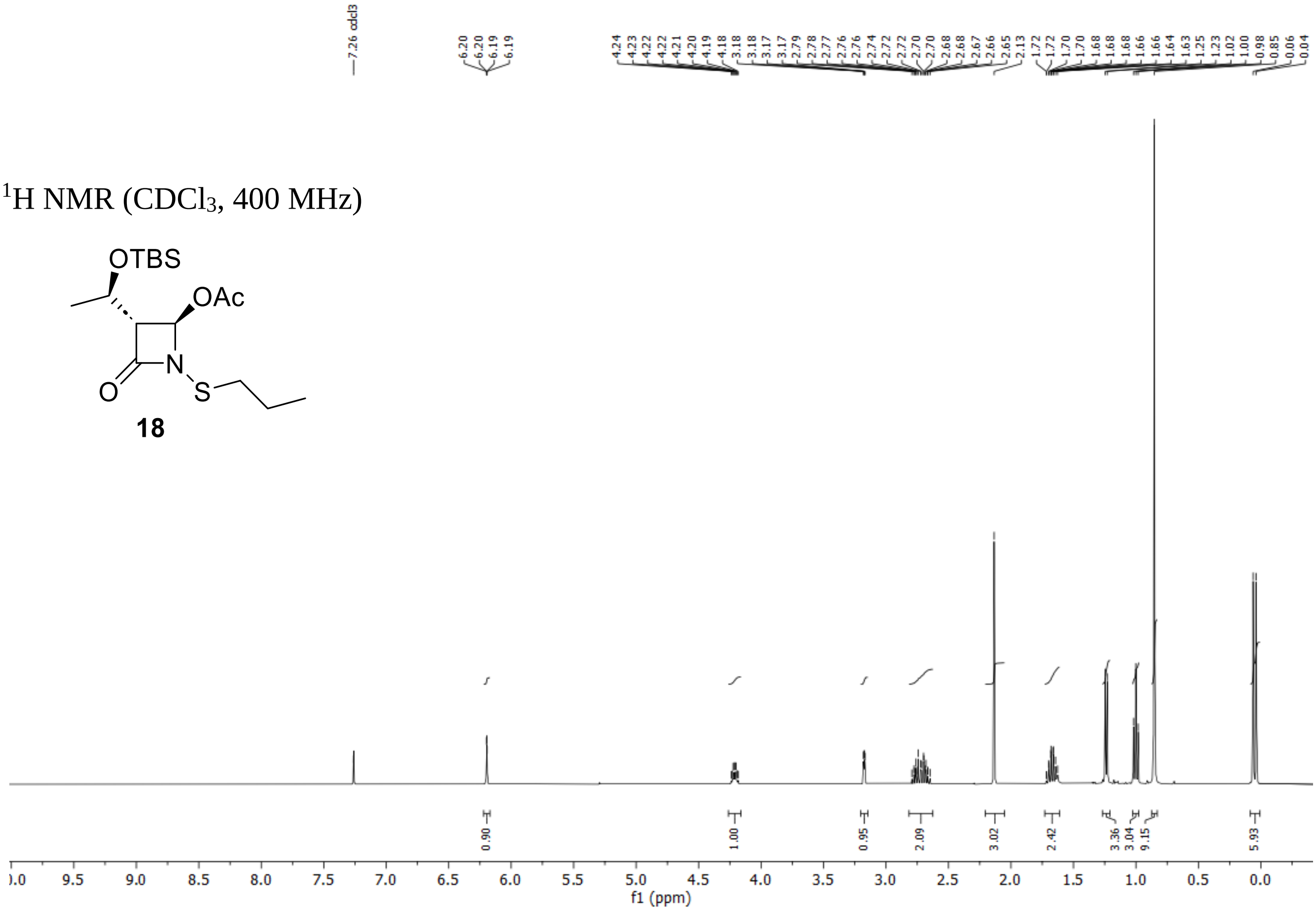

^{1}H NMR ($CDCl_3$, 400 MHz)

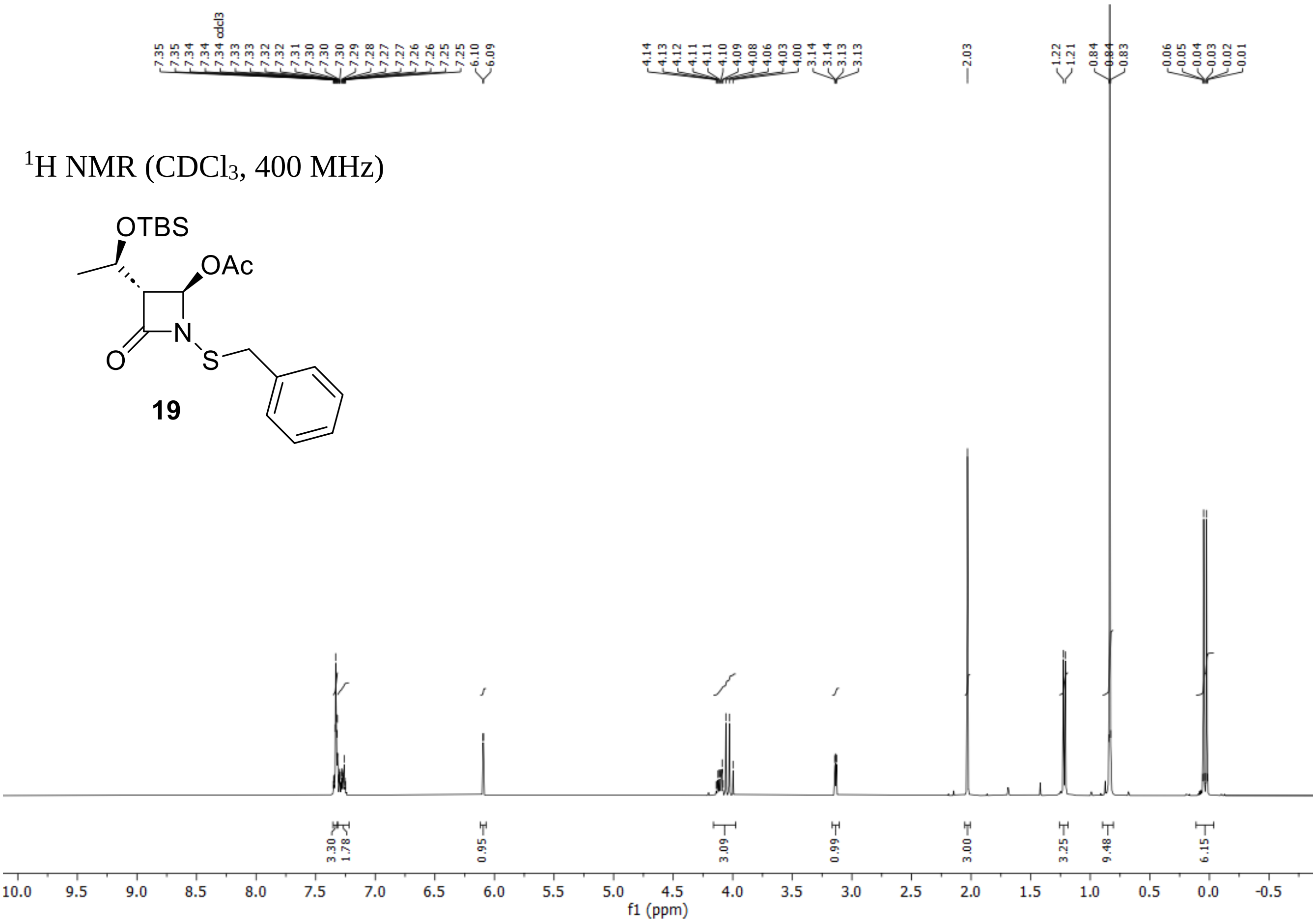

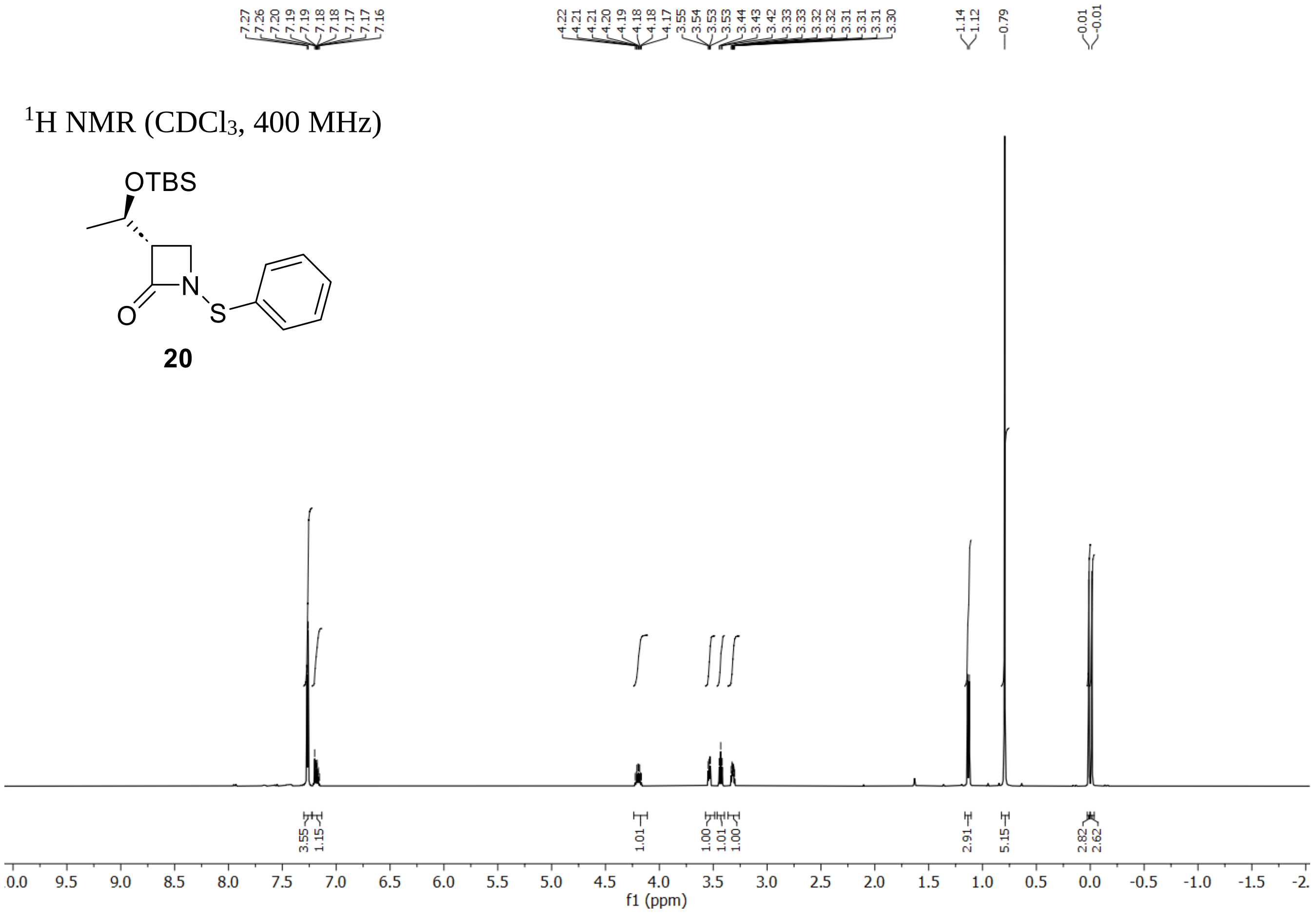

7.27
7.26
7.20
7.19
7.19
7.18
7.18
7.17
7.17
7.16
4.22
4.21
4.21
4.20
4.19
4.18
4.18
4.17
3.55
3.54
3.53
3.53
3.44
3.43
3.42
3.33
3.33
3.32
3.32
3.31
3.31
3.31
3.30
1.14
1.12
0.79
0.01
-0.01
^{1}H NMR ($CDCl_3$, 400 MHz)
OTBS
N
S
O
20
3.55
1.15
1.01
1.00
1.01
1.00
2.91
5.15
2.82
2.62
f1 (ppm)

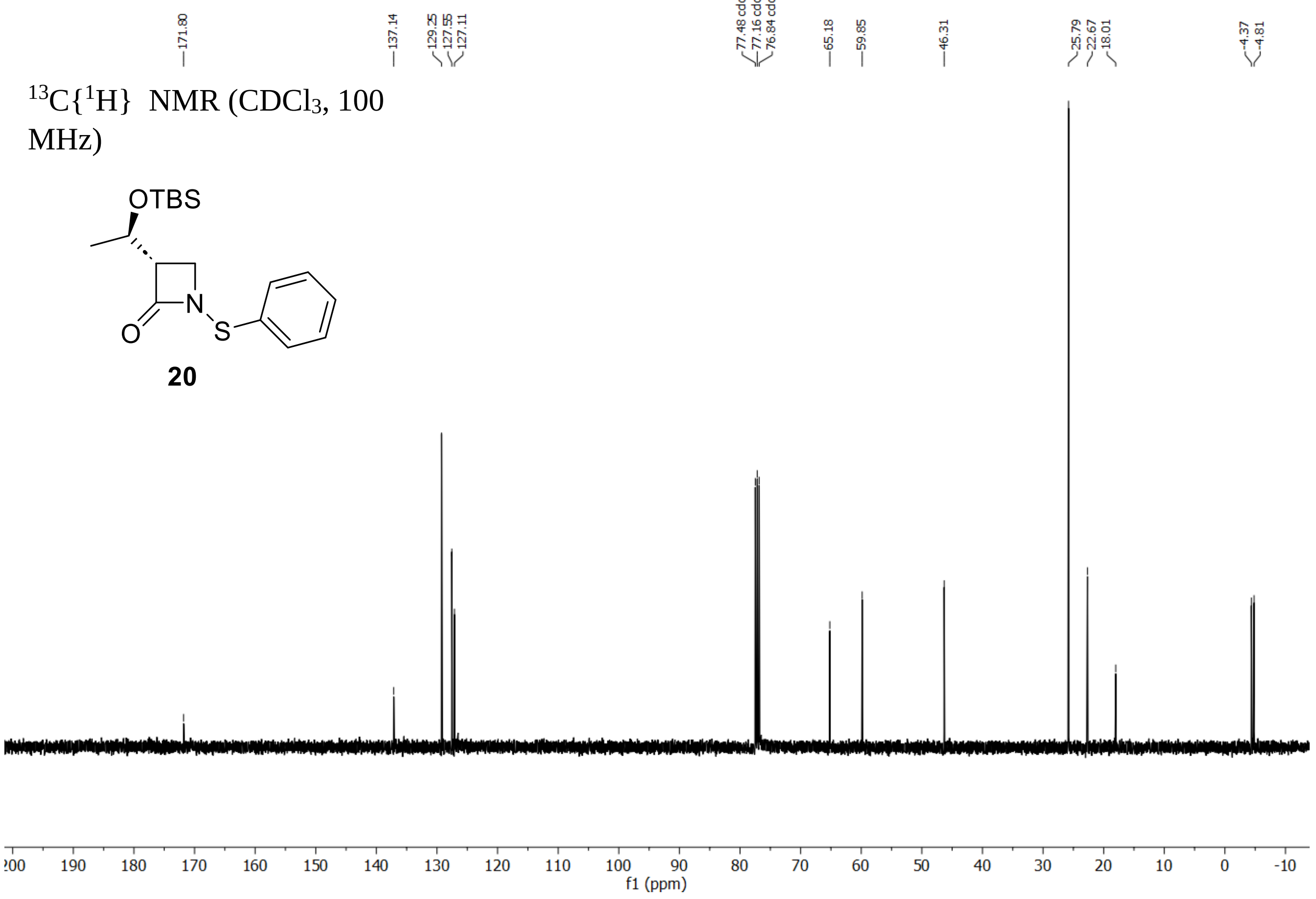

171.80
137.14
129.25
127.55
127.11
77.48 cdcl3
77.16 cdcl3
76.84 cdcl3
65.18
59.85
46.31
25.79
22.67
18.01
-4.37
-4.81
^{13}C{^{1}H} NMR ($CDCl_3$, 100 MHz)
OTBS
N
S
O
20
f1 (ppm)

^{1}H NMR ($CDCl_3$, 400 MHz)

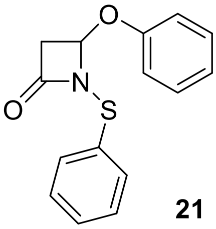

21

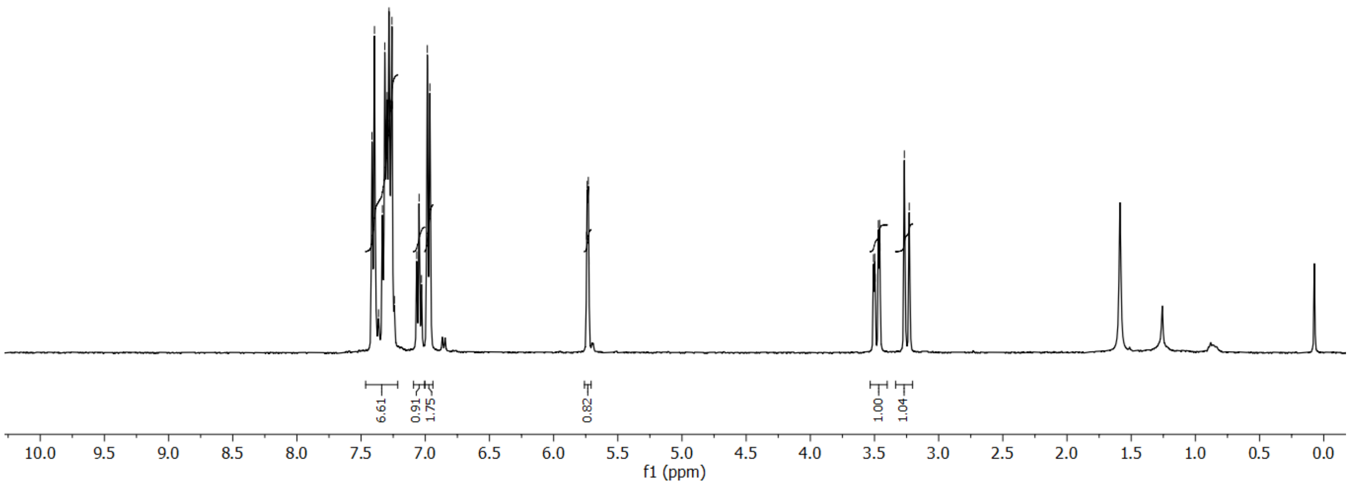

$^{13}C\{^{1}H\}$ NMR ($CDCl_3$, 100 MHz)

21

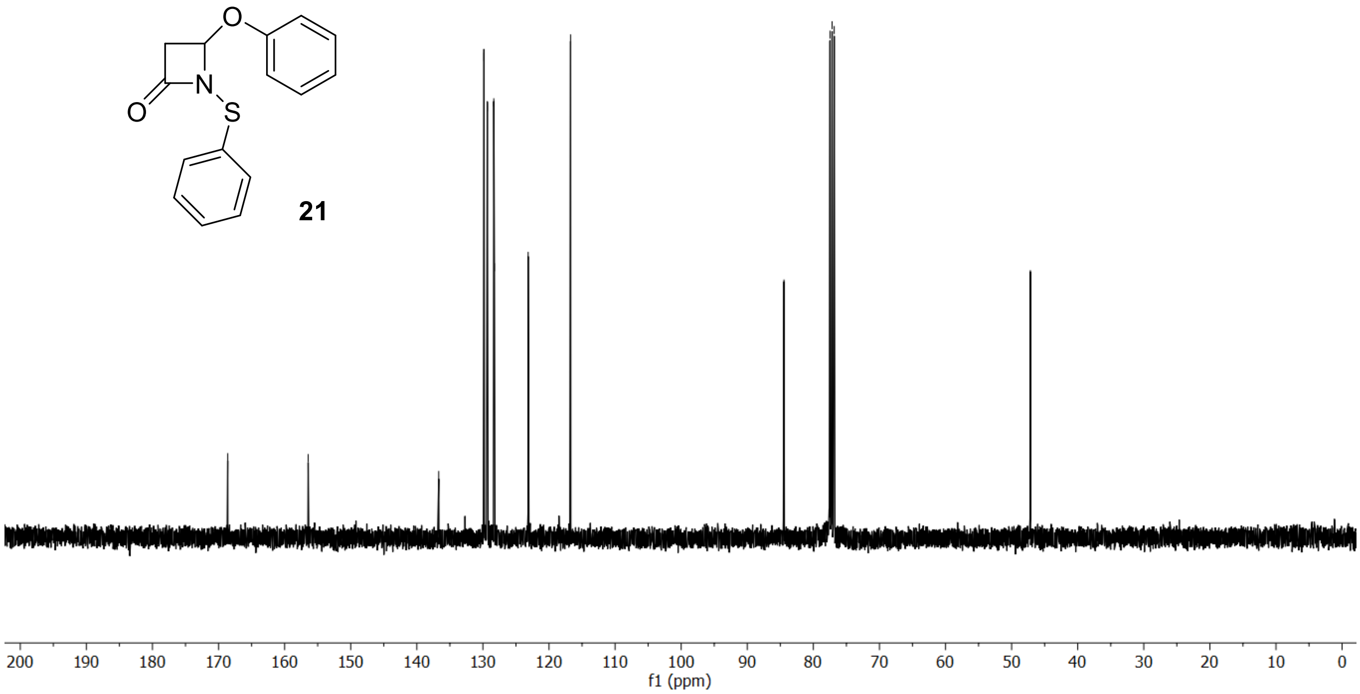

HPLC-MS spectra of N-thio β-lactams (Compounds 5-21)

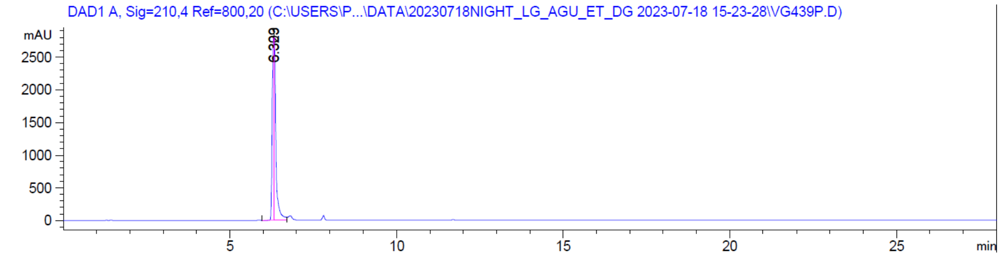

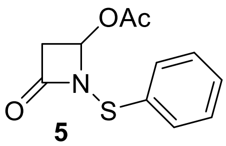

5

MW: 237,27 g/mol

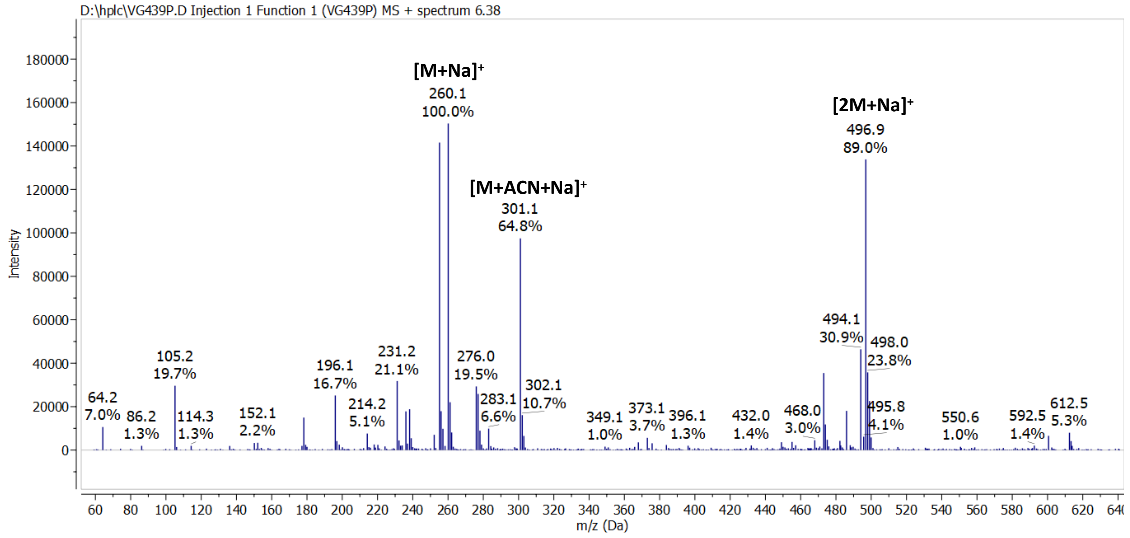

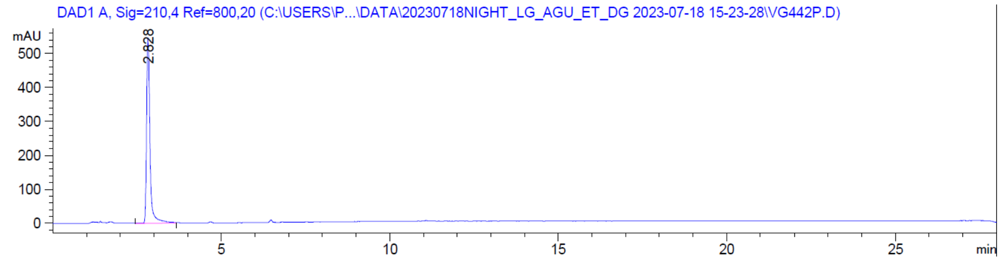

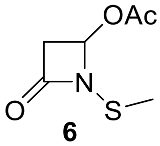

6

MW: 175,20 g/mol

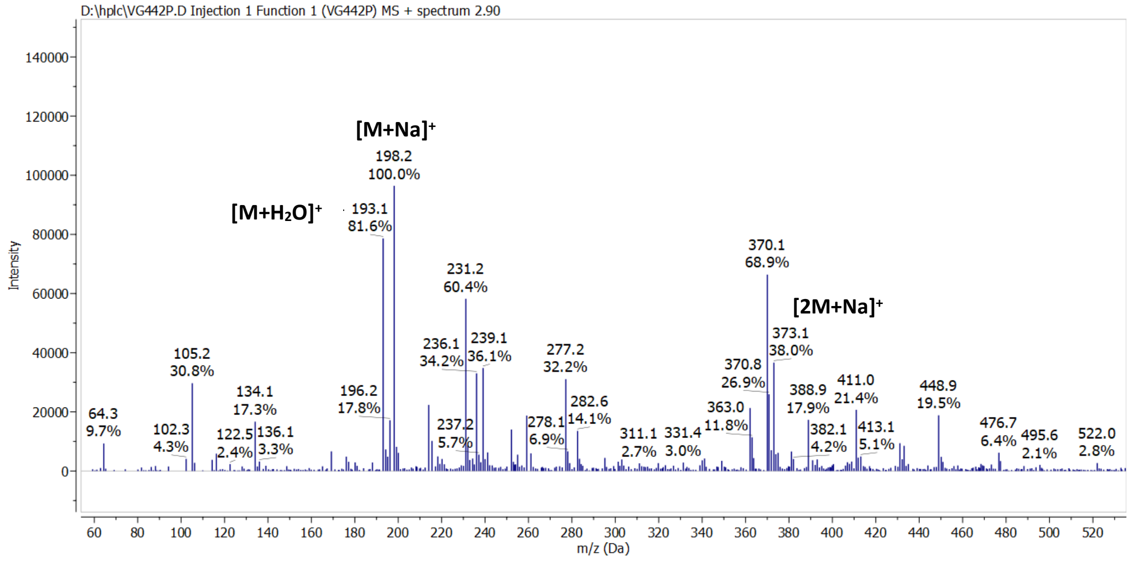

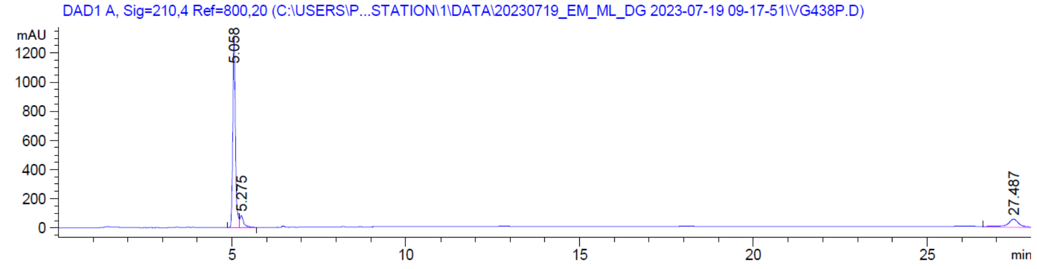

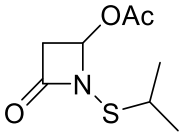

7

MW: 203,26 g/mol

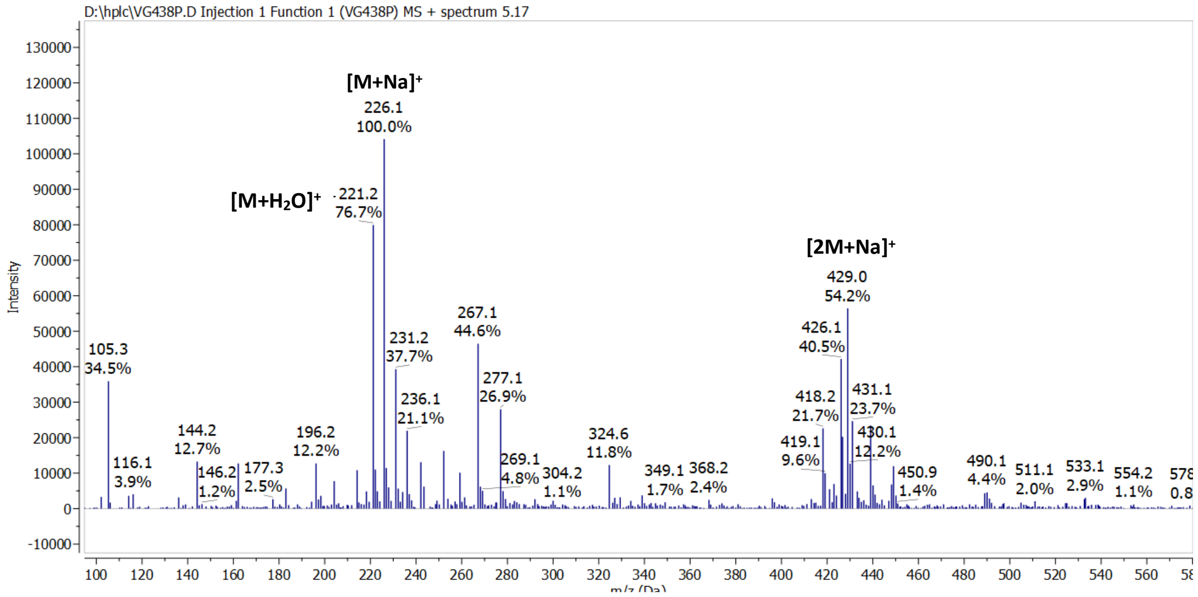

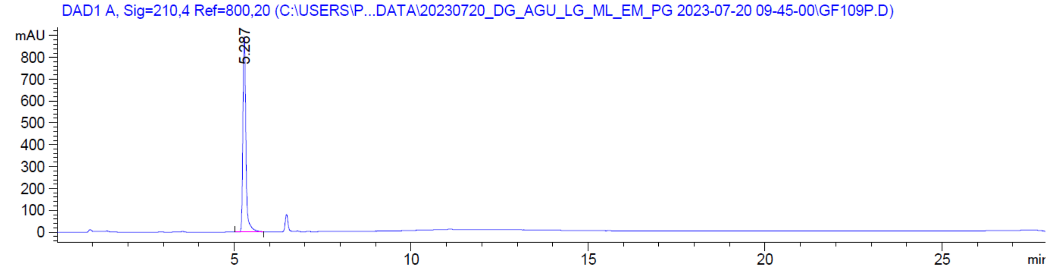

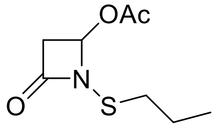

13

MW: 203,26 g/mol

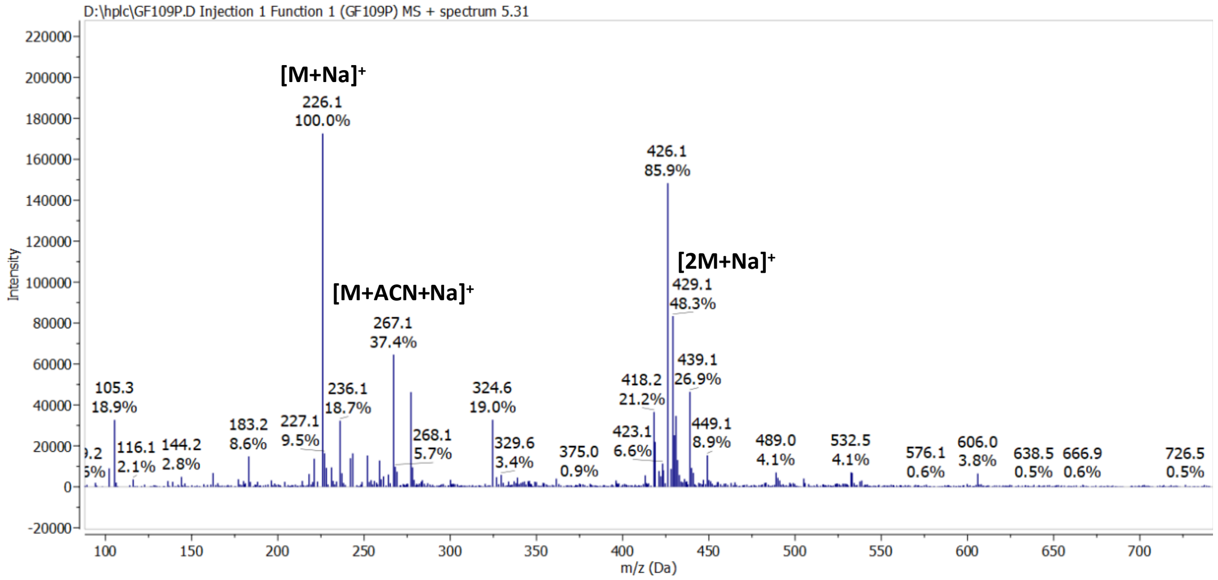

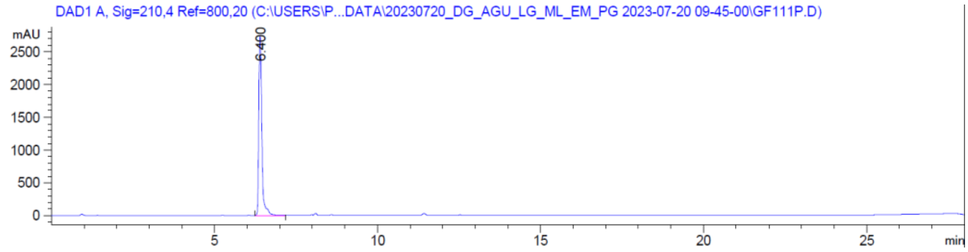

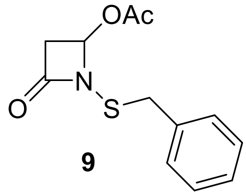

9

MW: 251,30 g/mol

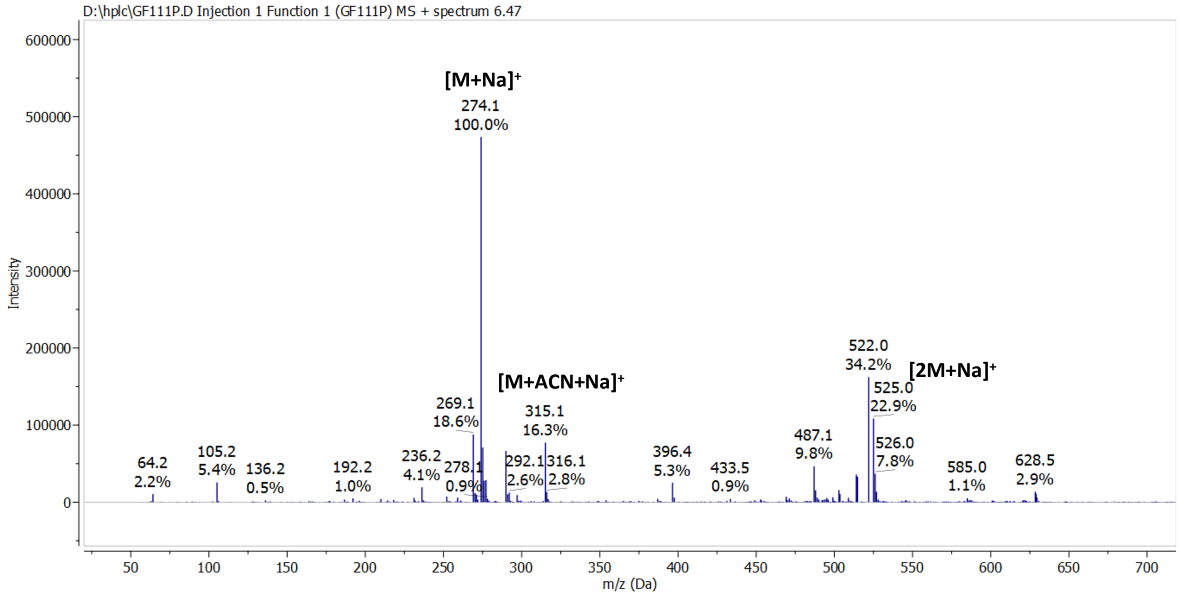

10

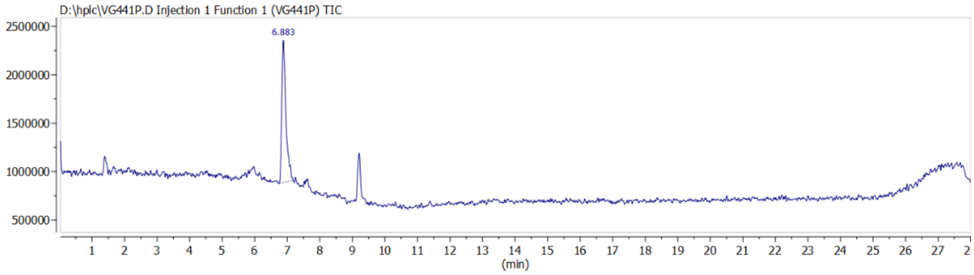

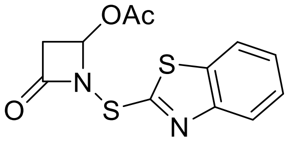

10

MW: 294,34 g/mol

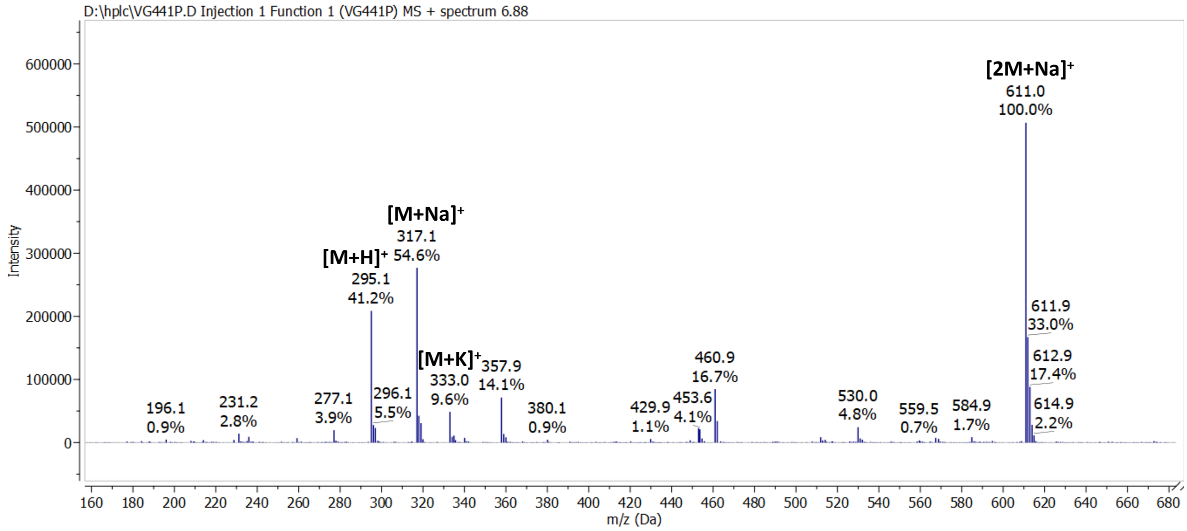

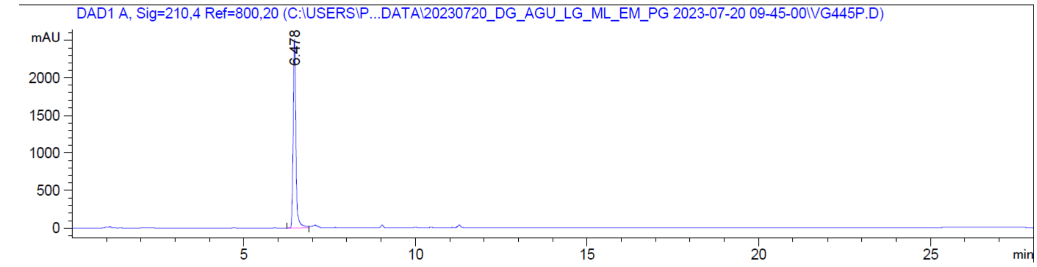

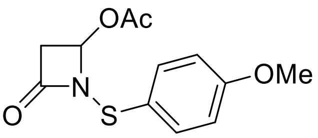

11

MW: 267,30 g/mol

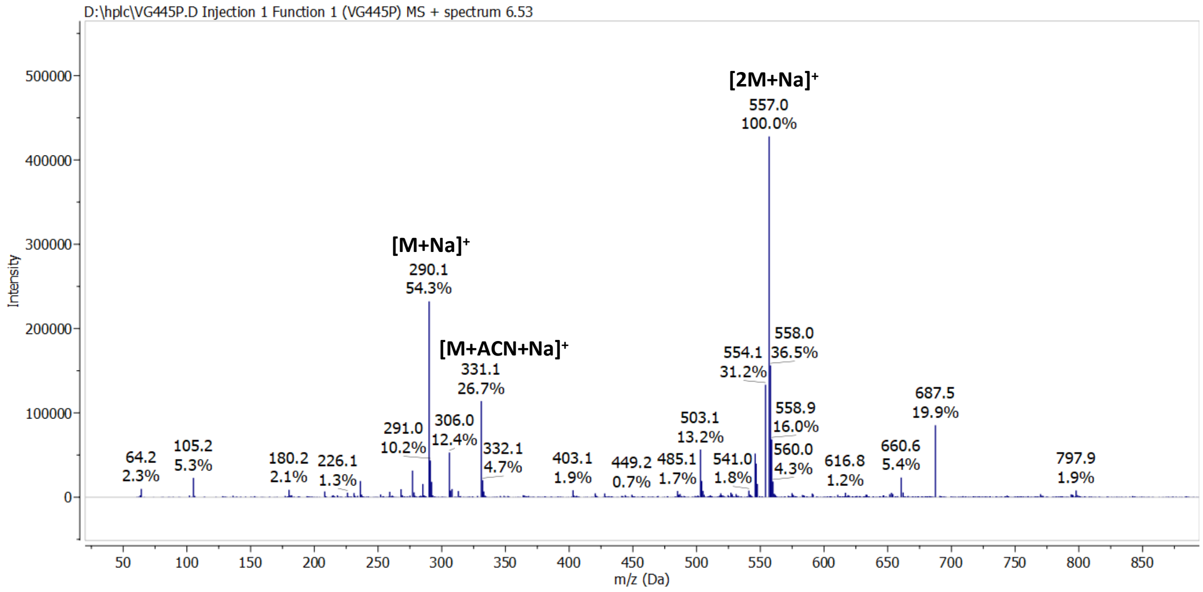

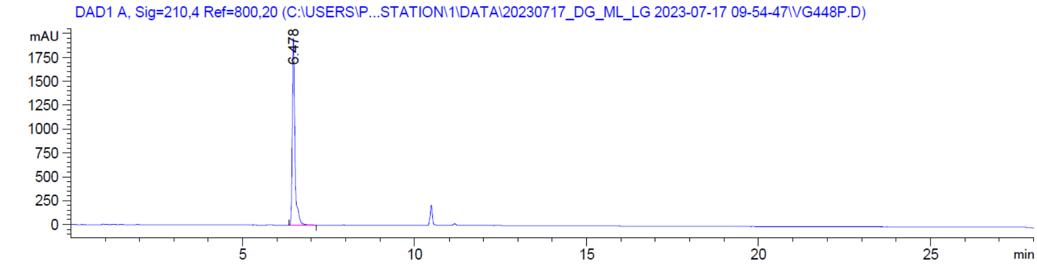

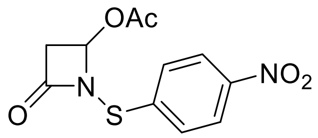

12

MW: 282,27 g/mol

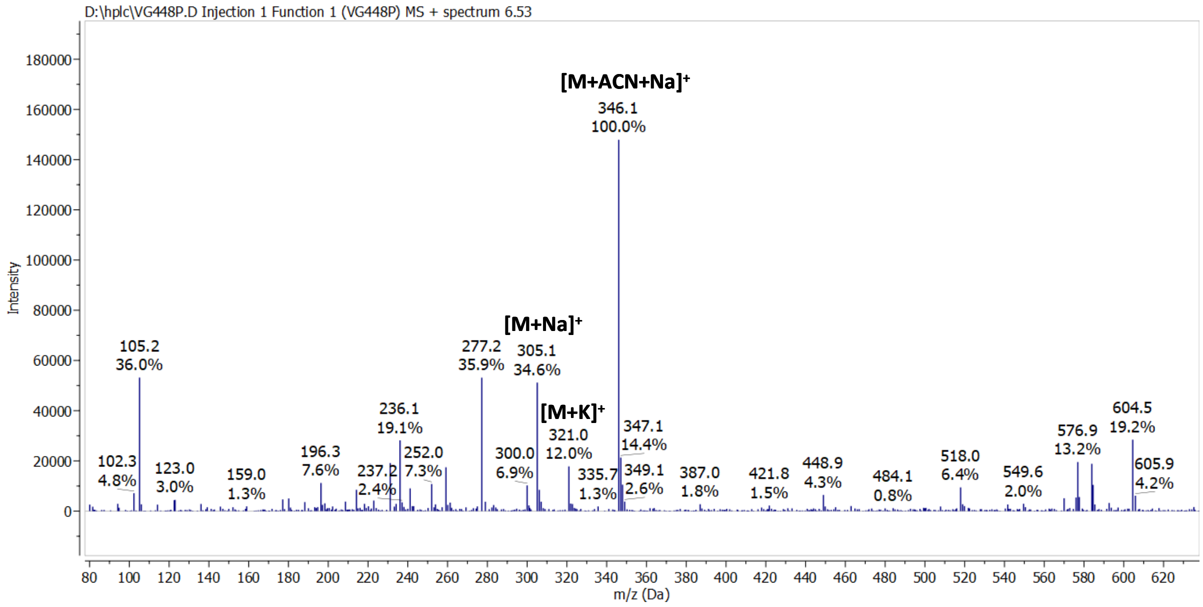

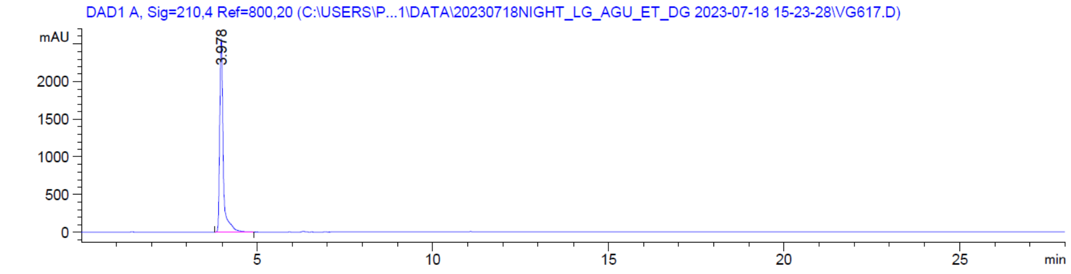

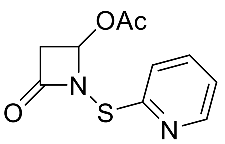

13

MW: 238,26 g/mol

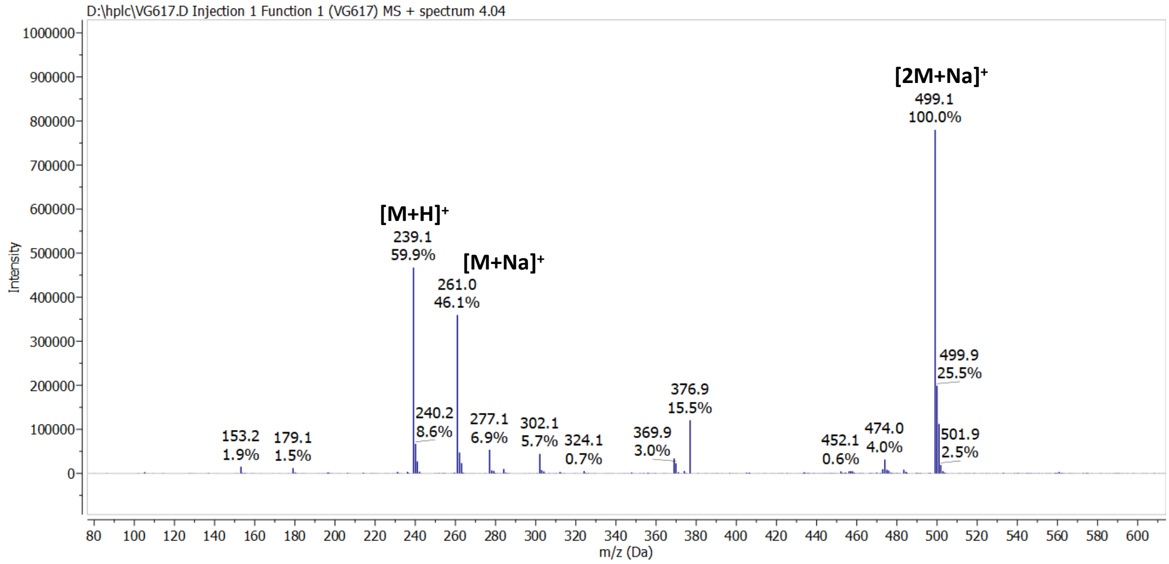

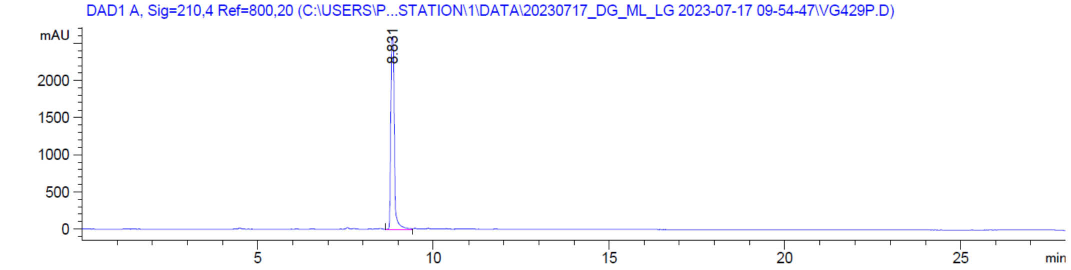

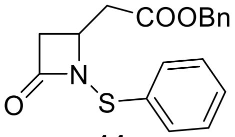

14

MW: 327,40 g/mol

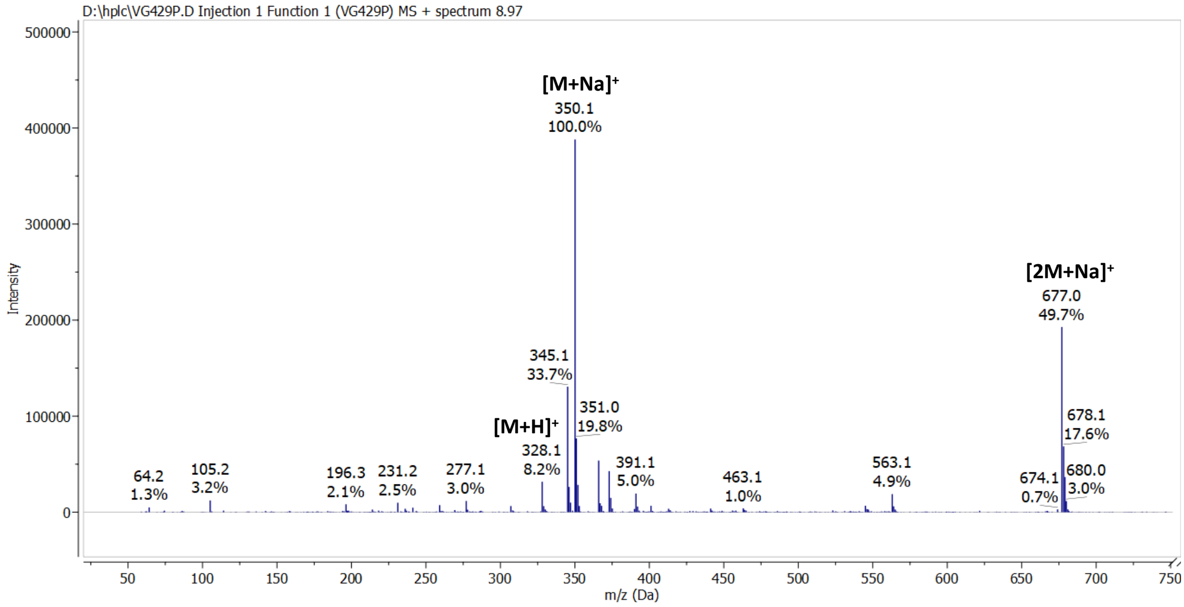

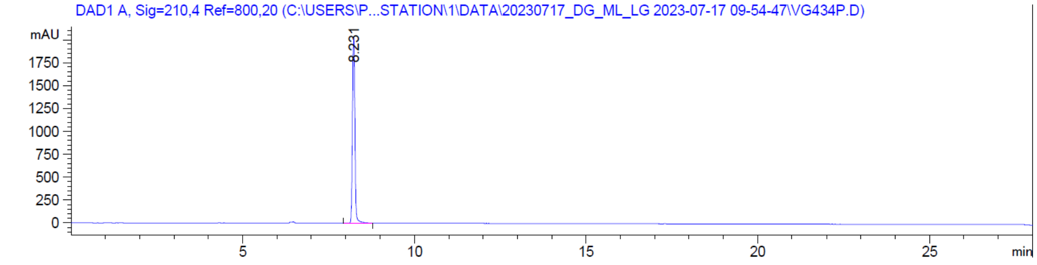

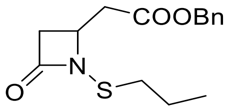

15

MW: 293,38 g/mol

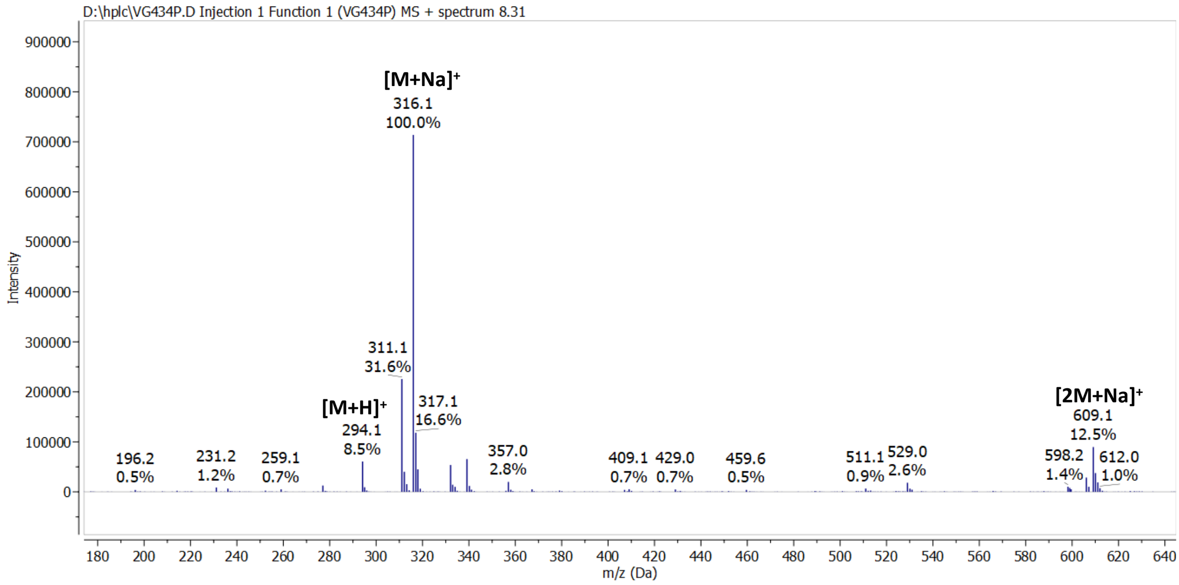

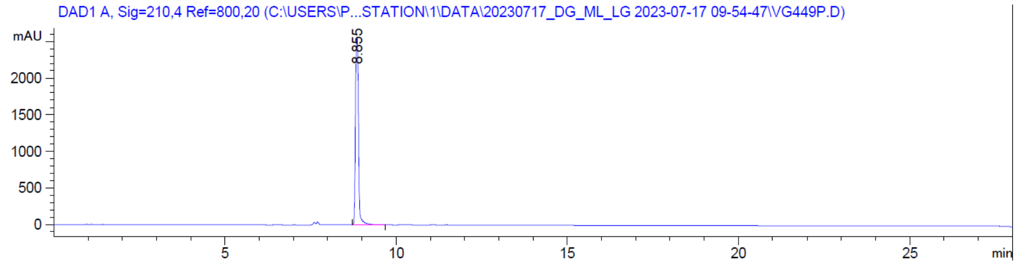

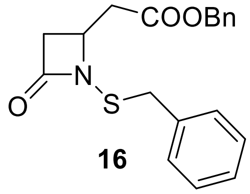

16

MW: 341,43 g/mol

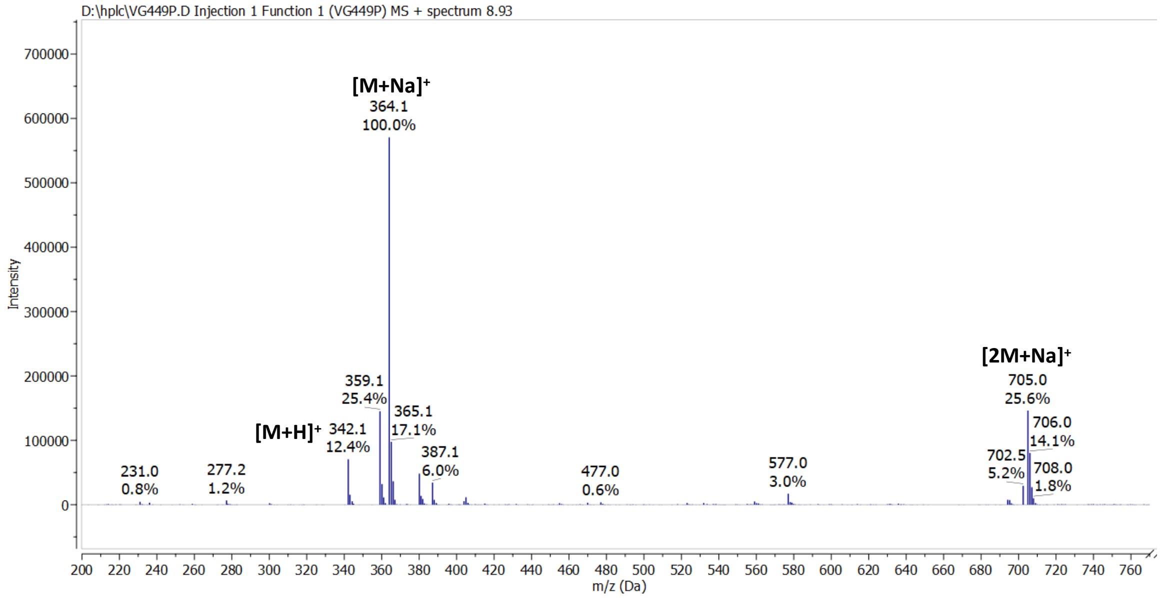

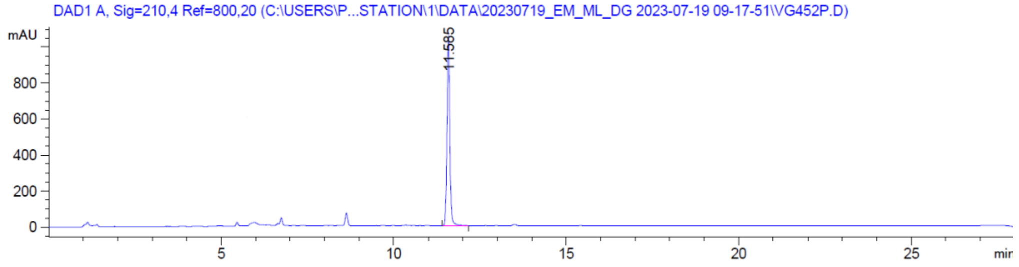

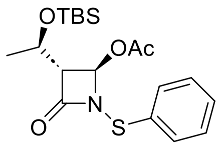

17

MW: 395,59 g/mol

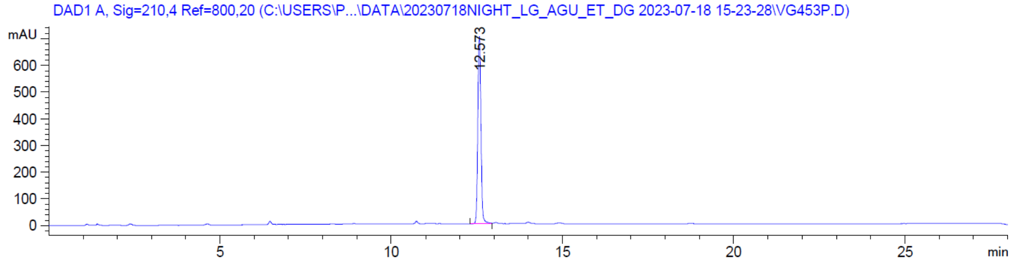

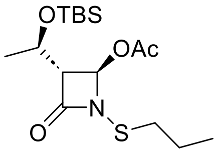

18

MW: 361,57 g/mol

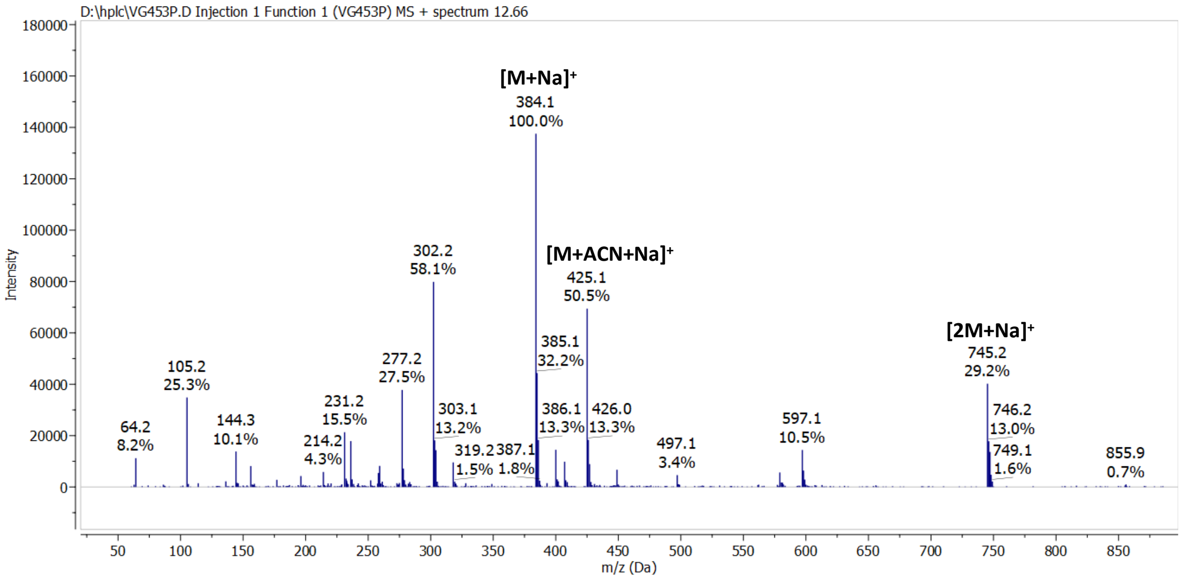

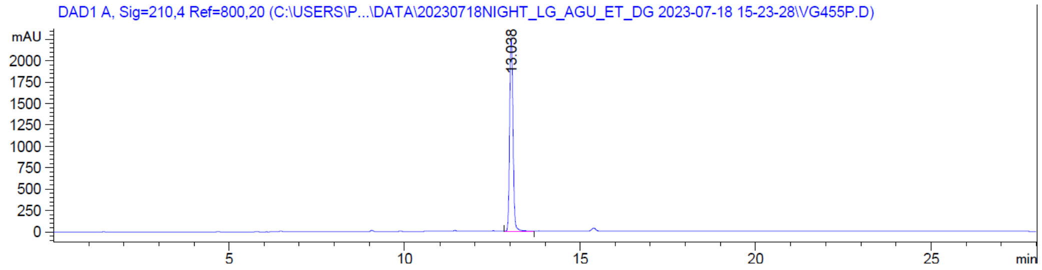

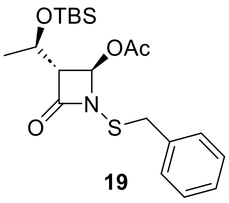

19

MW: 409,62 g/mol

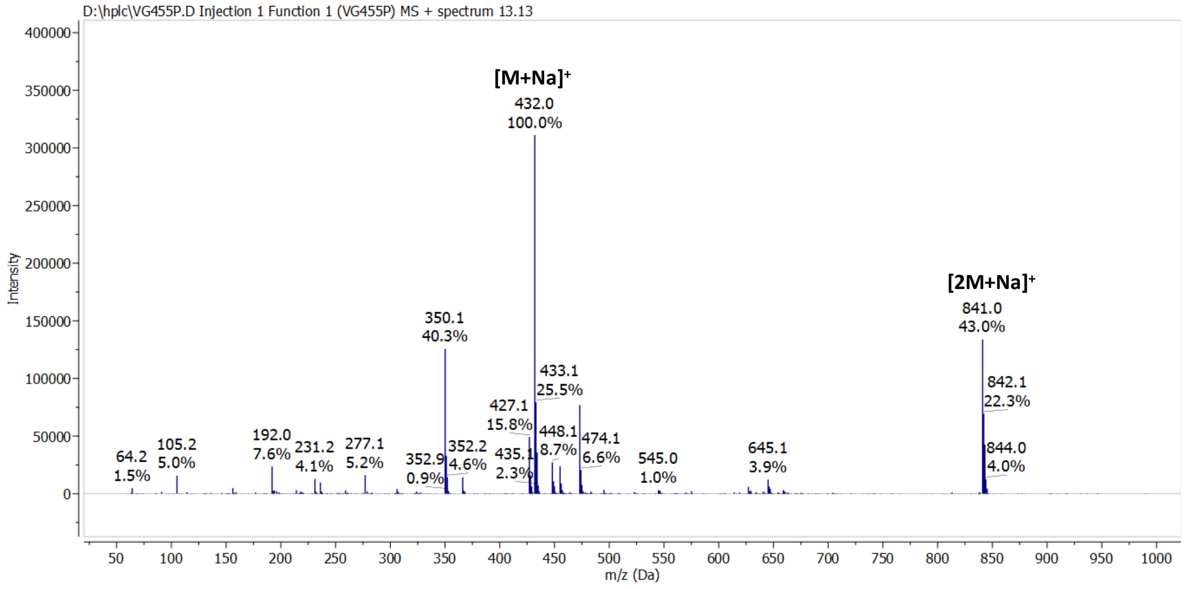

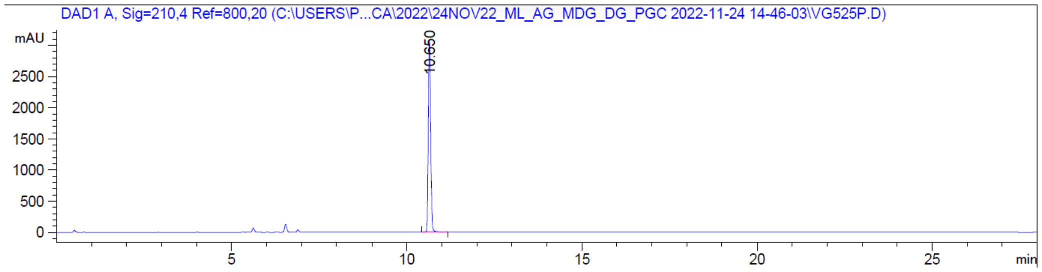

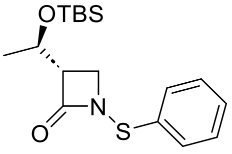

20

MW: 337,55 g/mol

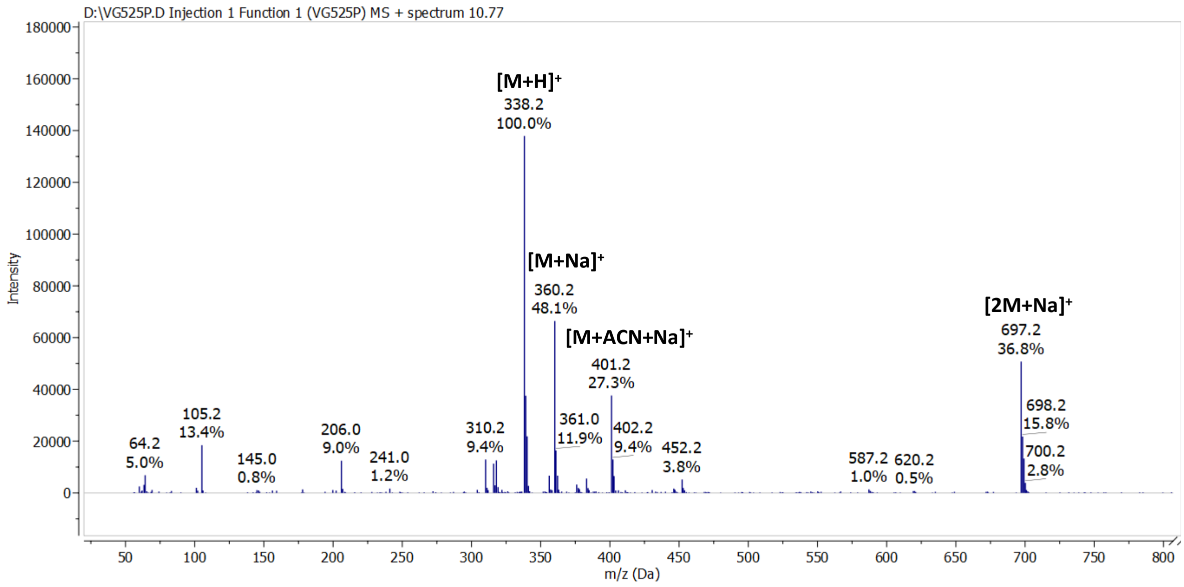

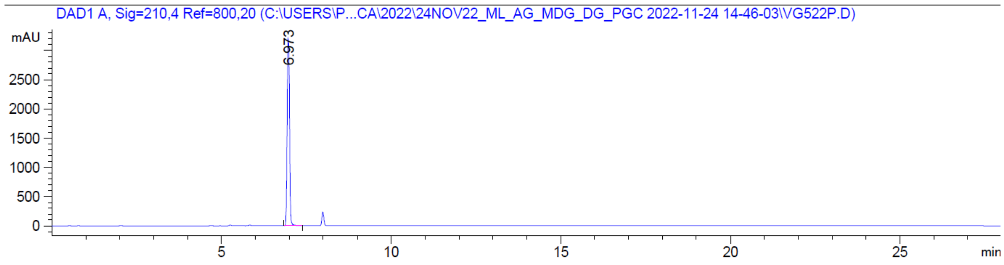

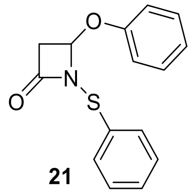

21

MW: 271,33 g/mol

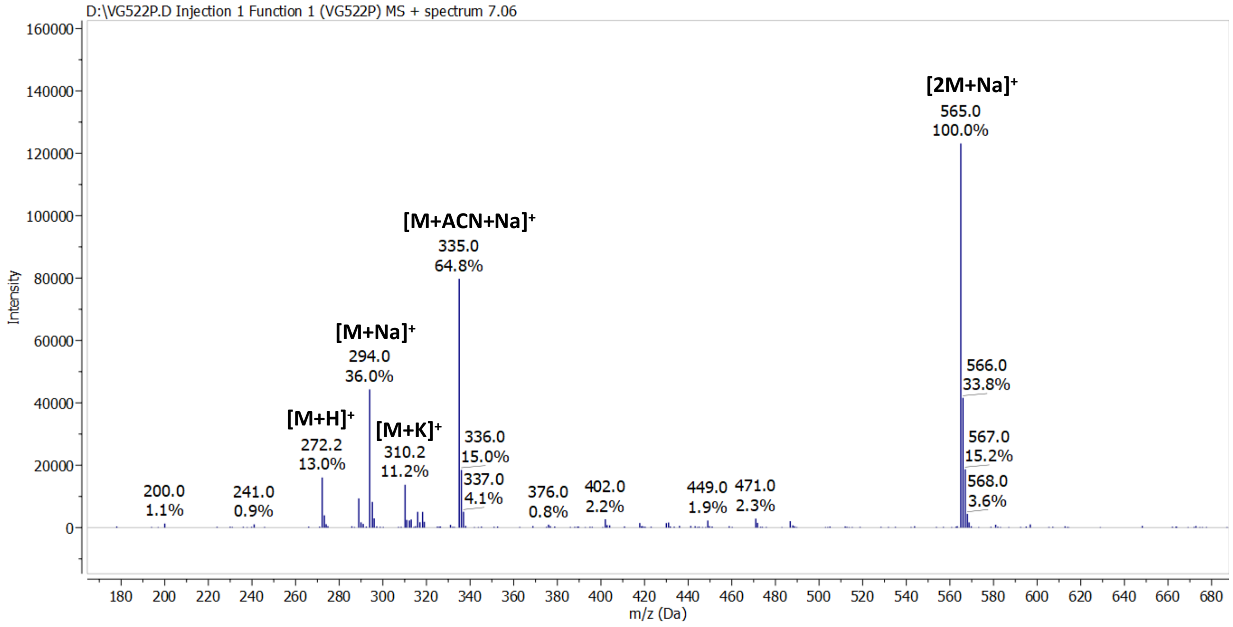

References

[1] P. Galletti, C. E. A. Cocuzza, M. Pori, A. Quintavalla, R. Musumeci, D. Giacomini, *ChemMedChem* **2011**, *6*, 1919-1927

[2] P. Galletti, R. Soldati, M. Pori, M. Durso, A. Tolomelli, L. Gentilucci, S.D. Dattoli, M. Baiula, S. Spampinato, D. Giacomini. Targeting integrins αvβ3 and α5β1 with new β-lactam derivatives. *Eur J Med Chem.* **2014**, 83, 284-93. doi: 10.1016/j.ejmech.2014.06.041.

[3] T. N. Beck, D. Lloyd, R. Kuskovsky, J. Minah, K.Arora, B. J. Plotkin, J. M. Green, H. I. Boshoff, C. Barry III, J. Deschamps, M. I. Konaklieva. Non-transpeptidase binding arylthioether β-lactams active against Mycobacterium tuberculosis and Moraxella catarrhalis. *Bioorg Med Chem*, **2015**, 23, 632 – 647

[4] C. Crauste, M. Froeyen, J. Anné, P. Herdewijn Asymmetric Synthesis of New β-Lactam Lipopeptides as Bacterial Signal Peptidase I Inhibitors. *Eur J Org Chem*, **2011**, 3437–3449

[5] G. Martelli, T.B. Pessatti, E.M. Steiner, M. Cirillo, C. Caso, F. Bisognin, M. Landreh, P.D. Del Monte, D. Giacomini, R. Schnell. *N*-thio-β-lactams targeting L,D-transpeptidase-2, with activity against drug-resistant strains of Mycobacterium tuberculosis. *Cell Chem. Biol.* **2021**, *28*, 1321-1332, DOI: 10.1016/j.chembiol.2021.03.008.
